# PI3Ka-Akt1-mediated Prdm4 induction in adipose tissue increases energy expenditure, inhibits weight gain, and improves insulin resistance in diet-induced obese mice

**DOI:** 10.1038/s41419-018-0904-3

**Published:** 2018-08-29

**Authors:** No-Joon Song, Seo-Hyuk Chang, Suji Kim, Vanja Panic, Byung-Hyun Jang, Ui Jeong Yun, Jin Hee Choi, Zhen Li, Ki-Moon Park, Jung-Hoon Yoon, Sunghwan Kim, Jae Hyuk Yoo, Jing Ling, Kirk Thomas, Claudio J. Villanueva, Dean Y. Li, Jee-Yin Ahn, Jin-Mo Ku, Kye Won Park

**Affiliations:** 1 Department of Food Science and Biotechnology, Sungkyunkwan University, Suwon, 16419 Korea; 2 Department of Biochemistry, University of Utah School of Medicine, 15N Medical Drive East Room 4100, Salt Lake City, UT 84112 USA; 3 New Drug Development Center, Daegu-Gyeongbuk Medical Innovation Foundation, Daegu, 41061 Korea; 4 Department of Medicine, Program in Molecular Medicine, University of Utah, 15 North 2030 East, Salt Lake City, UT 84112 USA; 5 Department of Molecular Cell Biology, Samsung Biomedical Research Institute, Sungkyunkwan University School of Medicine, Suwon, 16419 Korea; 6 Biomaterials Research and Development Team, Bio-Center, Gyeonggido Business Science Accelerator, Suwon, 16229 Korea

## Abstract

Stimulation of white adipose tissue (WAT) browning is considered as a potential approach to treat obesity and metabolic diseases. Our previous studies have shown that phytochemical butein can stimulate WAT browning through induction of Prdm4 in adipocytes. Here, we investigated the effects of butein on diet-induced obesity and its underlying molecular mechanism. Treatment with butein prevented weight gains and improved metabolic profiles in diet-induced obese mice. Butein treatment groups also displayed higher body temperature, increased energy expenditure, and enhanced expression of thermogenic genes in adipose tissue. Butein also suppressed body weight gains and improved glucose and insulin tolerance in mice housed at thermoneutrality (30 °C). These effects were associated with adipose-selective induction of Prdm4, suggesting the role of Prdm4 in butein-mediated anti-obese effects. To directly assess the in vivo role of Prdm4, we generated aP2-Prdm4 transgenic mouse lines overexpressing Prdm4 in adipose tissues. Adipose-specific transgenic expression of Prdm4 recapitulated the butein’s actions in stimulating energy expenditure, cold tolerance, and thermogenic gene expression, resulting in prevention of obesity and improvement of metabolism. Mechanistically, direct inhibition of PI3Kα activity followed by selective suppression of its downstream Akt1 mirrored butein’s effect on Ucp1 expression and oxygen consumption. In addition, effects of butein were completely abolished in Akt1 KO mouse embryonic fibroblasts. Together, these studies demonstrate the role of butein in obesity and metabolic diseases, further highlighting that adipose PI3Kα–Akt1–Prdm4 axis is a regulator of energy expenditure.

## Introduction

Increased calorie intake with less energy expenditure has led to an epidemic of obesity with subsequent development of various metabolic diseases, including diabetes, hypertension, cardiovascular diseases, and increased cancer risk^1–3^. Surgical and medical strategies for restricting appetite and increasing energy expenditure are continuously being developed to treat obesity and its related diseases^4^. However, new molecular targets and safe alternatives are still lacking.

Adipocytes play central roles in energy homeostasis of vertebrates^5,6^. White adipose tissue (WAT) stores excess energy and circulates adipokines, whereas brown adipose tissue (BAT) generates heat from oxidation of stored energy through the action of uncoupling protein 1 (Ucp1)^7^. Recent studies have revealed the existence of BAT in adult humans and the association between BAT activity and lower body mass in different populations, bringing new attention to brown fat as a therapeutic target for treating metabolic diseases^8–12^. Brown adipocytes with high expression of Ucp1 have been found in interscapular depots of rodents^6^. Other thermogenic cells as clusters of adipocytes have been found in WAT. These cells are referred to as beige adipocytes, brite (brown in white), or brown-like adipocytes^13–15^. Both brown and beige adipocytes are characterized by high mitochondrial contents and are believed to exhibit similar functions in energy metabolism^6,13,16^. However, the identification of beige-specific cell surface markers and different origins of these adipocytes indicate that beige cells are unique adipocytes, different from classical WAT or BAT^17,18^.

It has been shown that WAT browning in mice can suppress obesity and metabolic diseases. Transgenic expression of Prdm16 or Ucp1 in fat tissues can promote the generation of brown-like adipocytes in WAT (WAT browning), conferring resistance to obesity with improved glucose tolerance^19–21^. Stimulation of β3-adrenergic receptor or exercise can convert WAT to brown-like adipocytes through induction of Pgc-1α and Ucp1^22–24^. Subsequent studies have shown that exercise-induced production of β-aminoisobutyric acid can promote WAT browning in mice^25^. Similarly, it has been shown that small molecules such as berberine derived from a Chinese medicinal plant, salsalate derived from salicylic acid, and bexarotene (a retinoid X receptor agonist) can activate thermogenesis, resulting in increased energy expenditure in mice^26–28^. These observations raise the possibility that pharmacological induction of thermogenic adipocytes might serve as a new therapeutic strategy to combat obesity and its related metabolic diseases.

Previously, we have shown that phytochemical butein can stimulate the generation of thermogenic adipocytes through induction of Prdm4^29,30^. PRDI-BF1 and RIZ homology domain (Prdm) containing proteins are characterized by the presence of a PR domain, shared homology with the catalytic suppressor of variegation 3–9, Enhancer of zeste and Trithorax domain and a variable number of Zn-finger repeats^31^. Prdm4 is identified as a binding protein of p75 neurotrophin receptor and may provide a downstream transducer for the effects of nerve growth factor^32,33^. Mice homozygous for a deletion at the Prdm4 locus develop normally and adult mice are fertile and healthy^34^, suggesting the functional redundancy of Prdm4 during development. Here, we showed that butein reduced body weight and improved glucose tolerance through increasing energy expenditure and Prdm4 induction. Adipose-specific expression of Prdm4 enhanced thermogenesis and prevented obesity and metabolic diseases. Further mechanism studies showed that butein induced Ucp1 and WAT browning through targeting PI3Kα-Akt1-mediated signaling. Together, these data highlight the potential of using butein to treat obesity and its related diseases.

## Results

### Butein stimulates thermogenic gene expression in lean mice

We have previously shown that small molecule butein can stimulate Ucp1 expression and thermogenic programming in white adipocytes^30^. To examine the in vivo effect of butein, we intraperitoneally (i.p.) injected wild-type lean mice with butein (15 mg/kg per day) or vehicle control for 2 weeks and performed gene expression analysis. Expression levels of thermogenic adipocyte markers *Ucp1*, *Prdm16*, *Cox8b*, and *Cidea* were significantly induced in inguinal WAT (iWAT) compared with those in the control group (Supplementary Figure S1). Conversely, white adipocyte-selective genes *resistin* (*Retn*) and *nicotinamide N-methyltransferase* (*Nmmt*) along with pan-adipocyte markers *Pparγ* and *aP2* were repressed by butein. Similar induction of brown adipocyte gene expression by butein was also observed in epididymal WAT (eWAT) and BAT (Supplementary Figure S1). These data suggest that butein can induce *Ucp1* and thermogenic gene expression in mice.

### Butein treatment prevents weight gain and improves glucose tolerance in HFD-fed obese mice

**Fig. 1:**
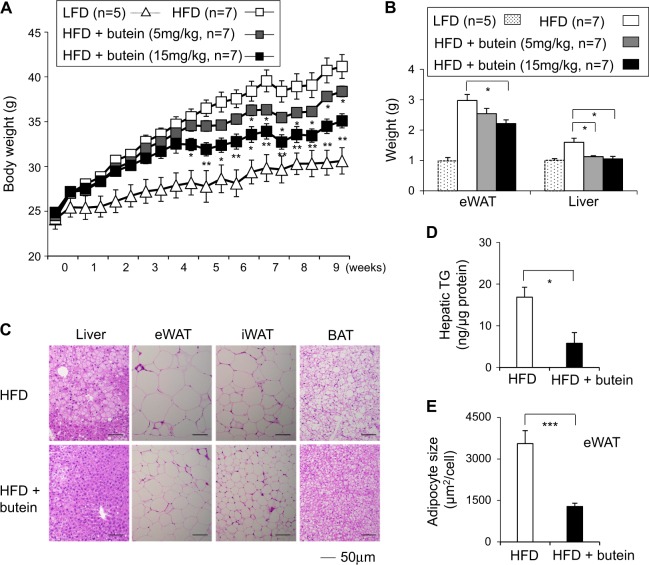
Butein prevents obesity in HFD-fed obese mice. **a** Body weight gain of vehicle control- or butein-treated mice. Male C57BL/6J mice were fed with a low-fat diet (LFD, 10% fat) or high-fat diet (HFD, 60% fat) and treated with vehicle control or butein (5 mg/kg and 15 mg/kg per day) for 8 weeks. Mice were weighed twice per week. **b** Differences in epididymal fat (eWAT) and liver weight in groups given daily intraperitoneal injection of 5 mg/kg or 15 mg/kg butein. **c** Representative hematoxylin and eosin (H&E) staining for sections of liver, epididymal fat (eWAT), inguinal fat (iWAT), and brown adipose tissue (BAT) from HFD-fed mice. Scale bar, 50 µm. **d** Quantification of liver triacylglycerols (TG) levels per protein contents (µg) in HFD-fed mice (*n* = 5) treated with vehicle control or butein for 8 weeks. **e** Adipocyte sizes from butein-treated eWAT were smaller than those of control HFD-fed mice. Data represent mean ± s.e.m. Statistically significant differences between control- and butein-treated mice were determined by Student’s *t*-test. *P < 0.05; **P < 0.005; ***P < 0.0005

**Fig. 2:**
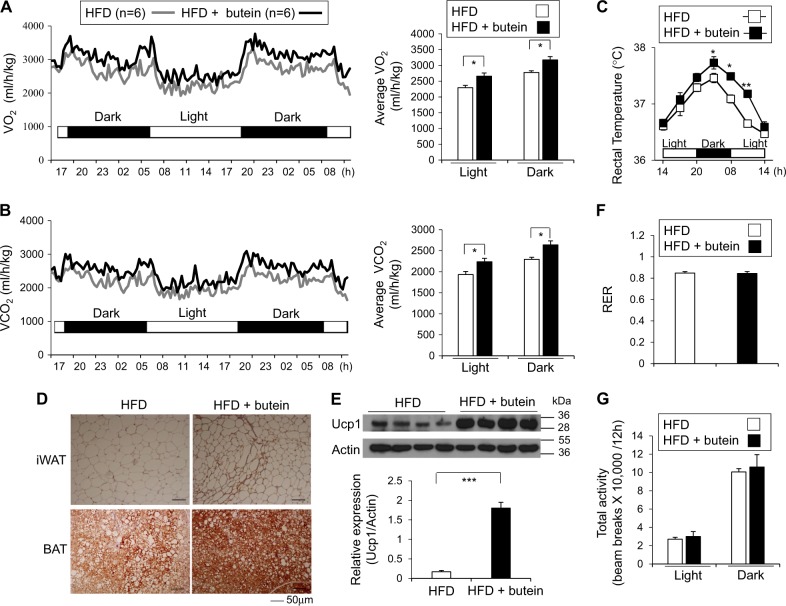
Butein increases energy expenditure in HFD-fed obese mice. **a, b** Energy expenditure was measured by oxygen consumption and carbon dioxide production. **a** O_2_ consumption and **b** CO_2_ production rates of control (*n* = 6) and butein-injected mice (15 mg/kg per day, *n* = 6) were measured by indirect calorimetry using CLAMS after 3 weeks on HFD (*n* = 6 per group). Energy expenditure was measured before body weights started to diverge. Bar graph (right panel) represents the average of O_2_ consumption or CO_2_ production in each group. **c** Rectal temperature for 24 h was measured in mice treated with control or butein. **d, e** Increased Ucp1 protein expression in adipose tissue from butein-treated mice. **d** Representatives of Ucp1 staining in iWAT and BAT of control or butein-treated mice for 8 weeks are shown. Scale bar, 50 µm. **e** Expression levels of Ucp1 protein in iWAT of control (*n* = 4) and butein-injected mice (15 mg/kg per day, *n* = 4) were determined by western blotting and quantified. **f** Respiratory exchange ratio (RER), **g** total physical activities of control- and butein-treated mice for 3 weeks (*n* = 6 per group). Data represent mean ± s.e.m. and statistically significant differences between control- andr butein-treated mice were determined by Student’s *t*-test. Statistically significant differences were determined by two-way ANOVA for the rectal temperature. *P < 0.05; **P < 0.005; ***P < 0.0005

Given the effect of butein (15 mg/kg per day) on thermogenic gene expression in lean mice, we investigated the anti-obese effect of butein in diet-induced obese mice. We injected butein (5 mg/kg per day or 15 mg/kg per day) into mice fed with high-fat diet (HFD) for 8 weeks. Butein treatment significantly reduced body weight gains of mice fed with HFD compared with vehicle control injection (Fig. 1a). Liver and eWAT from butein-treated HFD mice (15 mg/kg per day) weighed less than those from control mice (Fig. 1b). Histological observation and lipid analysis further revealed reduced triglyceride accumulation in the liver and reduced adipocyte size in adipose tissue in butein-treated HFD mice (Figs. 1c-e). Abdominal adipose tissue (1642 mm^3^) in butein-treated HFD mice was significantly lesser than that of HFD control group (2482 mm^3^). Butein also decreased levels of fasting serum cholesterol and fatty acids (Supplementary Figure S2). However, serum alanine aminotransferase (ALT) or aspartate transaminase (AST) levels were not significantly different (Supplementary Figure S2). Insulin resistance was improved by butein treatment based on glucose tolerance and insulin tolerance tests (Supplementary Figure S3). We measured energy expenditure in HFD-fed mice treated with control or butein for 3 weeks when body weights were not significantly different between the groups. Metabolic analysis showed increased O_2_ consumption and CO_2_ production in HFD mice treated with butein compared with vehicle control-treated HFD mice (Figs. 2a, b). Consistently, diurnal rectal temperature in butein-treated group was significantly higher with greater differences during night time than that in the control group (Fig. 2c). Brown-like histological conversion by butein treatment was further corroborated by increased protein expression levels of Ucp1 in iWAT and BAT (Figs. 2d, e). Other thermogenic genes, such as *Prdm16*, *Cox8b*, and *Cidea*, were also induced by butein in iWAT. Expression levels of pan-adipocyte and white adipocyte-selective mRNA in iWAT were suppressed by butein (Supplementary Figure S4). Similar trends toward brown-like adipogenic induction were also observed in eWAT and BAT (Supplementary Figure S4). Food intake, respiratory exchange ratio (RER), and physical activity were similar between the control and butein-treated groups (Supplementary Figure S5, and Figs. 2f, g). These data showed that increased energy expenditure could be the primary cause of reduced obesity in mice fed HFD. In parallel experiments, butein treatment exhibited a trend to reduce body weight gains compared with control treatments in low-fat diet (LFD) fed mice, but failed to reach statistical significance (Supplementary Figure S6). Therefore, butein can be a potential tool to prevent the prevalence of obesity and its associated metabolic diseases.

The mouse has a higher surface area to volume ratio than humans, resulting in a significantly greater thermal challenge under a given ambient temperature exposure^35^. To better mimic the thermal conditions experienced by humans^36^, we investigated the effects of butein in HFD-fed mice housed at 30 °C. Butein treatments significantly prevented HFD-induced body weight gains compared with the control group (Supplementary Figure S7). Epididymal fat pads and liver weights were lower in butein-treated mice. Similar to the effects in mice housed at 23 °C, butein treatments significantly improved glucose tolerance and insulin sensitivity in HFD-fed mice housed at thermoneutral conditions (Supplementary Figure S7).

### Adipose-specific expression of butein-responsive gene Prdm4 prevents weight gains and fat mass in HFD-fed obese mice

Previous data showed a critical role of Prdm4 in butein-mediated thermogenic induction in adipocytes. To evaluate this finding in mice, we measured expression levels of Prdm4 in various tissues from mice treated with butein for 3 weeks. *Prdm4* expression was significantly increased in iWAT and eWAT from butein-treated mice. *Prdm4* expression in liver was only marginally increased in butein-treated group. However, its expression was not altered in other tissues (Supplementary Figure S8). These data suggest that butein-induced *Prdm4* in adipose tissue might take part in the stimulation of energy expenditure.

**Fig. 3:**
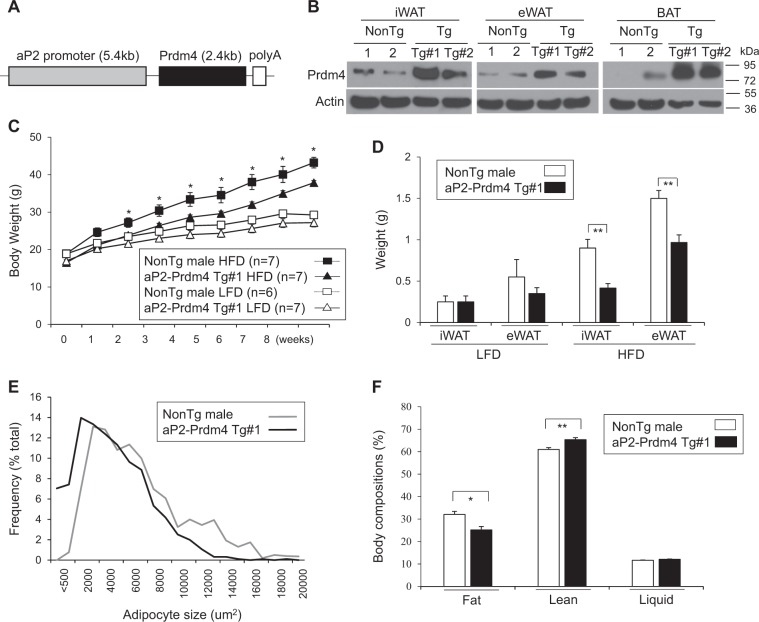
Prdm4 expression in adipose tissue (aP2-Prdm4) protect from obesity and fat expansion in HFD feeding. **a** A schematic transgenic construct used to generate adipose tissue-specific Prdm4 transgenic mice. **b** Representative Prdm4 and actin expression in iWAT, eWAT, and BAT from male littermate of non-transgenic (NonTg) and two lines of aP2-Prdm4 transgenic (Tg#1 and Tg#2) mice. **c** Body weight gains of Prdm4 Tg and NonTg male mice. Male NonTg or Prdm4 Tg mice were fed with a low-fat diet (LFD, 10% fat) or high-fat diet (HFD, 60% fat) for 8 weeks. (HFD: *n* = 7 (NonTg), *n* = 7 (Tg); LFD: *n* = 6 (NonTg), *n* = 7 (Tg)). **d** Differences in epididymal fat (eWAT) and inguinal fat (iWAT) weight gains in NonTg and Prdm4 Tg male mice. **e** Adipocyte size of epididymal fat (eWAT) from Prdm4 Tg and NonTg HFD-fed male mice. **f** Body composition of mice after 8 weeks of HFD-fed NonTg and Prdm4 Tg male mice. Data represent mean ± s.e.m. and statistically significant differences between control NonTg and Prdm4 Tg mice were determined by Student’s *t*-test. *P < 0.05; **P < 0.005

Having observed selective induction of Prdm4 by butein in adipose tissue, we hypothesized that adipose Prdm4 might play a role in thermogenesis and obesity. To test this possibility, we created transgenic mice in which fat-specific aP2 gene promoter could direct Prdm4 expression in adipose tissues. It has been shown that the 5.4 kb aP2 promoter can direct transgenic expression of Prdm4 in fat tissue^37,38^. Transgenic expression of Ucp1 or Prdm16 in fat tissue can limit weight gain and decrease fat mass^37,39^. Similarly, aP2-driven Prdm4 transgene was selectively expressed in BAT and WAT without noticeable overexpression in other tissues (Figs. 3a, b, and Supplementary Figure S9). Prdm4 transgenic (Prdm4 Tg) mice exhibited about 3- to 6-folds overexpression in BAT and WAT compared with its levels in control non-transgenic littermates (NonTg) (Fig. 3b). Prdm4 Tg male mice (aP2-Prdm4 Tg#1) displayed significantly reduced body weight when they were fed HFD (Fig. 3c). Inguinal and epididymal depots were smaller in Tg mice and their adipocytes were less hypertrophic compared with those of HFD-fed NonTg mice (Figs. 3d-f). Similar anti-obese effects were observed in second transgenic lines (Tg#2) (Supplementary Figure S10), further showing the protective role of adipose Prdm4 in HFD-induced obesity.

### Adipose-specific induction of Prdm4 increases energy expenditure and cold tolerance

**Fig. 4:**
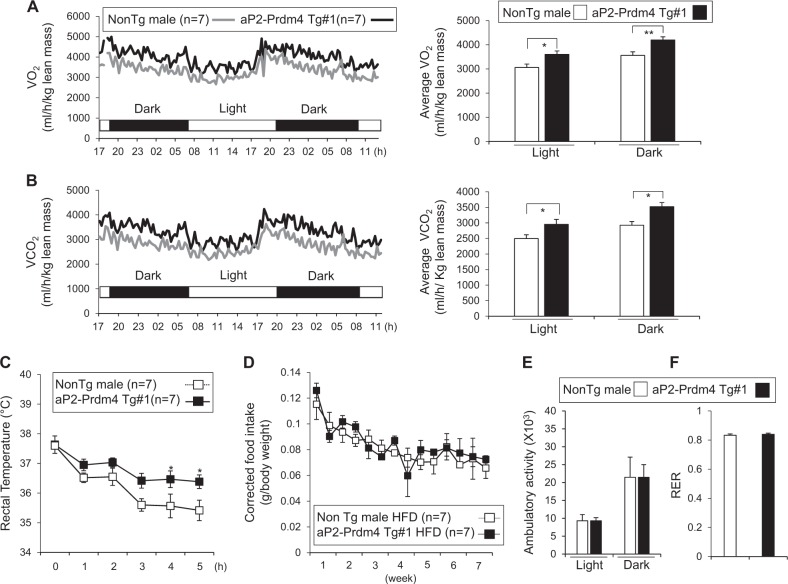
aP2-Prdm4 Tg male mice increase energy expenditure in HFD feeding. **a, b** Energy expenditure was evaluated by oxygen consumption and carbon dioxide production. **a** O_2_ consumption and **b** CO_2_ production rates of littermate control NonTg and Prdm4 Tg male mice were measured by indirect calorimetry using CLAMS after 6 weeks on HFD (*n* = 7 per group). Bar graph (right panel) represents the average of O_2_ consumption or CO_2_ production in each group. **c** Rectal temperature was measured in NonTg and aP2-Prdm4 Tg male mice during cold exposure at 4 °C. **d** Corrected food intake in NonTg and aP2-Prdm4 Tg male mice. **e** Ambulatory activities, and **f** Respiratory exchange ratio (RER) of NonTg and aP2-Prdm4 Tg HFD-fed mice (*n* = 7 per group). Data represent mean ± s.e.m. and statistically significant differences between control NonTg and aP2-Prdm4 Tg mice were determined by Student’s *t*-test. Statistically significant differences were determined by two-way ANOVA for the rectal temperature. *P < 0.05; **P < 0.005

**Fig. 5:**
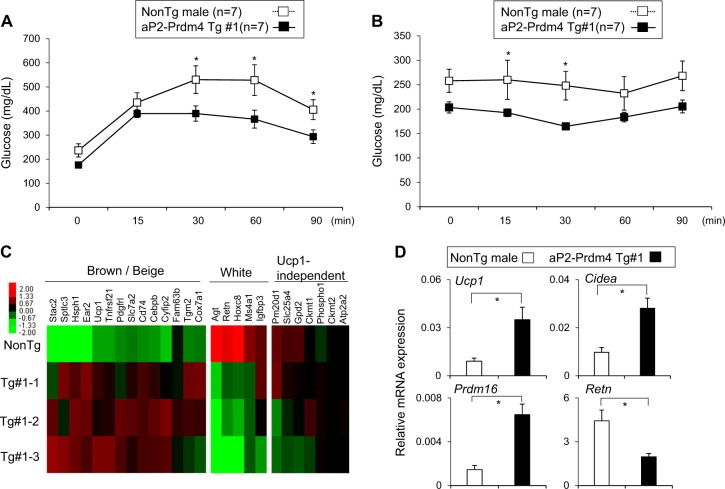
aP2-Prdm4 Tg mice improve glucose homeostasis and induce thermogenic program. **a** Glucose tolerance test and **b** insulin tolerance test in control NonTg (*n* = 7) and aP2-Prdm4 Tg group (*n* = 7). HFD-fed mice were fasted for 6 h before intraperitoneal injection of glucose (1 g/kg) or insulin (0.5 U/kg) for GTT and ITT experiments, respectively. Tail blood samples were collected at different time points to measure blood glucose levels. **c** Heat maps of relative expression levels of brown or beige-selective, white-selective, and Ucp1-independent genes in iWAT of aP2-Prdm4 Tg and NonTg mice. **d** Expression analysis of selected brown genes (*Ucp1*, *Cidea*, and *Prdm16*) and white adipocyte-selective gene (*Retn*) in iWAT of NonTg and aP2-Prdm4 Tg mice. Gene expression was determined by real-time PCR. Data were normalized to 36b4 and expressed as mean ± s.e.m. statistically significant differences in gene expression data were determined by Student’s *t*-test and two-way ANOVA was used to determine significance in GTT and ITT. *P < 0.05

To directly investigate the role of adipose Prdm4 in energy metabolism, we measured energy expenditure in transgenic and non-transgenic mice. Metabolic analysis showed increased O_2_ consumption and CO_2_ production in Prdm4 Tg mice compared with those in NonTg male HFD-fed mice (Figs. 4a, b). Consistent with enhanced energy expenditure, Prdm4 Tg mice were able to maintain body temperature better during acute cold exposure compared with NonTg mice (Fig. 4c). However, food intake, physical activity, and RER were not significantly different between Prdm4 Tg mice and NonTg mice (Figs. 4d-f). We also found that Tg mice exhibited improved glucose and insulin tolerance relative to NonTg mice (Figs. 5a, b). These phenotypic changes were associated with increased expression of thermogenic genes in iWAT of Prdm4 Tg mice (Figs. 5c, d). Known Ucp1-independent thermogenic players including *Pm20d1*, *Ckmt1*, and *Gpd2* were not differently expressed in these groups. Expression of pan-adipocyte and white adipocyte selective genes in iWAT was suppressed in Tg mice. Histological observation also showed reduced triglyceride accumulation in the liver and reduced adipocyte size in adipose tissue of Prdm4 Tg mice (Supplementary Figure S11). These effects were not gender specific as female transgenic mice also exhibited less weight gains, improved glucose tolerance test (GTT) and insulin tolerance test (ITT), enhanced energy expenditure, and higher body temperature without showing significant difference in food intake or physical activities (Supplementary Figure S12-15). These data showed that adipose-selective increased expression of Prdm4 could promote WAT browning, increase energy expenditure, and prevent obesity.

### A critical role of PI3K signaling in butein-mediated Prdm4 induction

**Fig. 6:**
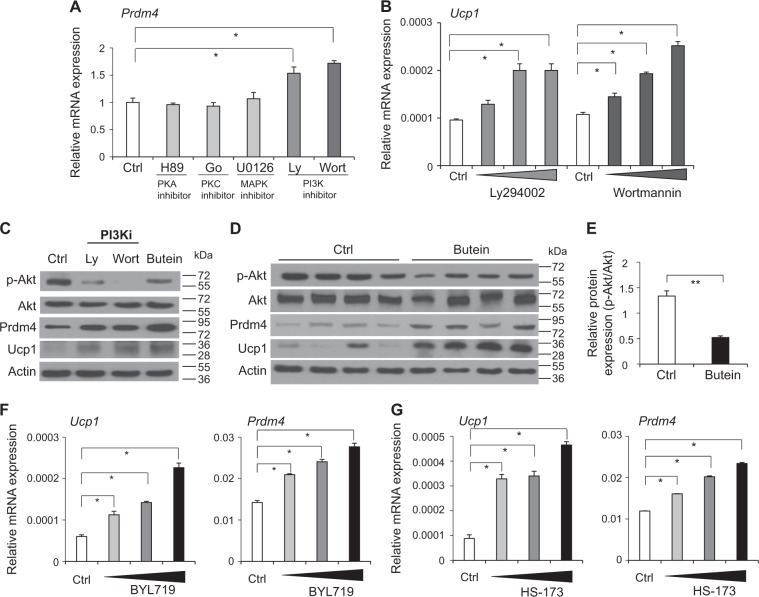
Butein regulates PI3Kα signaling pathway in adipocytes. **a** C3H10T1/2 adipocytes were treated with 20 μM of H89 (PKA inhibitor), Go6983 (PKC inhibitor), U0126 (MAPK kinase inhibitor), Ly294002 (PI3K inhibitor), or Wortmannin (PI3K inhibitor) for 6 h and Prdm4 mRNA expression levels were determined by real-time PCR. **b** Dose-dependent effect of Ly294002 (10, 20, and 40 μM) and Wortmannin (10, 20, and 40 μM) on *Ucp1* mRNA expression. **c** C3H10T1/2 adipocytes were treated with PI3K inhibitors (Ly294002 or Wortmannin) or butein and PI3K-mediated Prdm4, Ucp1, and Akt phosphorylation was determined by western blot analysis. **d** Akt phosphorylation, Akt, Prdm4, and Ucp1 levels in epididymal adipose tissue (eWAT) of butein or control treated obese C57BL/6J mice (*n* = 4 per group) were determined by western blot analysis. **e** Relative p-Akt/Akt levels in eWAT of mice treated with butein or control were quantified. **f, g** C3H10T1/2 adipocytes were treated with PI3Kα-selective inhibitors and mRNA expression levels were determined by real-time PCR. **f** Treatments with PI3Kα-selective inhibitor BYL719 (50, 100, and 200 µM) induced *Ucp1* and *Prdm4* mRNA expression in C3H10T1/2 adipocytes. **g** Effects of HS-173 (10, 20, and 40 µM), another PI3Kα-selective inhibitor, on *Ucp1* and *Prdm4* mRNA expression. Data represent mean ± s.e.m. and statistically significant differences were determined by Student’s *t*-test. *P < 0.05; **P < 0.005

Having determined that butein treatment or induction of Prdm4 in adipose tissue promoted energy expenditure, we focused on molecular mechanisms. To define molecular links between butein and Prdm4 in adipocytes, effects of butein on a kinase activity panel consisting of 51 different recombinant kinases, including phosphatidylinositol-4,5-bisphopsate 3-kinases (PI3K), epidermal growth factor receptor (EGFR), Met proto-oncogene (MET), AMP-activated protein kinase (AMPK), and inhibitor of kappa B kinase beta (IKKβ), were investigated. Interestingly, butein at 20 μM preferentially inhibited PI3Kα activity (62%). However, it exhibited only marginal effects on other kinases (Supplementary Figure S16). Although this does not exclude the possibility that butein might interact with other kinases, it suggests that butein is a relatively selective inhibitor of PI3Kα. We also examined the role of selected kinases (PI3K, protein kinase A (PKA), mapk signaling (MAPK), and protein kinase C (PKC)) in butein-mediated induction of Prdm4 and Ucp1. Treatment of C3H10T1/2 adipocytes with PI3K inhibitors LY294002 and Wortmannin resulted in increased *Prdm4* expression (Fig. 6a and Supplementary Figure S17). In contrast, alteration of PKA, MAPK, or PKC signaling failed to affect *Prdm4* levels (Fig. 6a). PI3K inhibition also increased *Ucp1* mRNA expression levels (Fig. 6b). We then measured phosphorylation levels of Akt, a downstream target protein of PI3K. Levels of phosphorylated Akt (S473) were suppressed by butein in C3H10T1/2 adipocytes (Fig. 6c). Reduced levels of Akt phosphorylation were consistently detected in adipose tissue from butein-treated mice (Figs. 6d, e). Furthermore, regulatory effects of butein on Akt and expression of *Prdm4* and *Ucp1* were also observed in human adipocytes (Supplementary Figure S18), further supporting that PI3K could be a molecular target of butein in adipocytes.

### Butein directly inhibits PI3Kα activity

PI3K is composed of four different classes (class I–IV) that transduce essential signals of mitogenic, cell survival, cytoskeletal remodeling, and metabolic controls. In particular, class I catalytic subunit p110α (PI3Kα) plays a role in energy expenditure, metabolism, and obesity^40–42^. To determine specific PI3K isoforms involved in the regulation of Prdm4 and Ucp1 induction, we investigated the effects of class I catalytic subunit inhibitors, including PI3K catalytic subunit α (PI3Kα)-selective BYL719, PI3Kβ-selective GSK2636771, PI3Kδ-selective IC-87114, and PI3Kγ-selective inhibitor AS-252424. BYL719, but not others, increased the expression of *Prdm4* and *Ucp1* expression (Fig. 6f, and Supplementary Figure S19 and S20). HS-173, another PI3Kα-selective inhibitor, also increased *Prdm4* and *Ucp1* mRNA and protein expression levels (Fig. 6g and Supplementary Figure S20). These PI3Kα-selective inhibitors induced mitochondrial mass and oxygen consumption rates (OCRs) (Supplementary Figure S20), further showing a close functional link between PI3Kα and Prdm4.

Given the specific effects of PI3Kα on Prdm4 and Ucp1 induction, we investigated whether butein could directly inhibit activities of purified PI3Kα. Our results revealed that butein exhibited inhibitory activities of PI3Kα, with an IC_50_ value of 6.4 µM (Supplementary Figure S21). However, butein did not display inhibitory effect on PI3Kβ, PI3Kδ, or PI3Kγ (Supplementary Figure S21). These data showed that butein could selectively target PI3Kα to induce Prdm4 expression in adipocytes.

### Selective inhibition on Akt1 is critical for butein’s effects on Prdm4 and Ucp1 induction

**Fig. 7:**
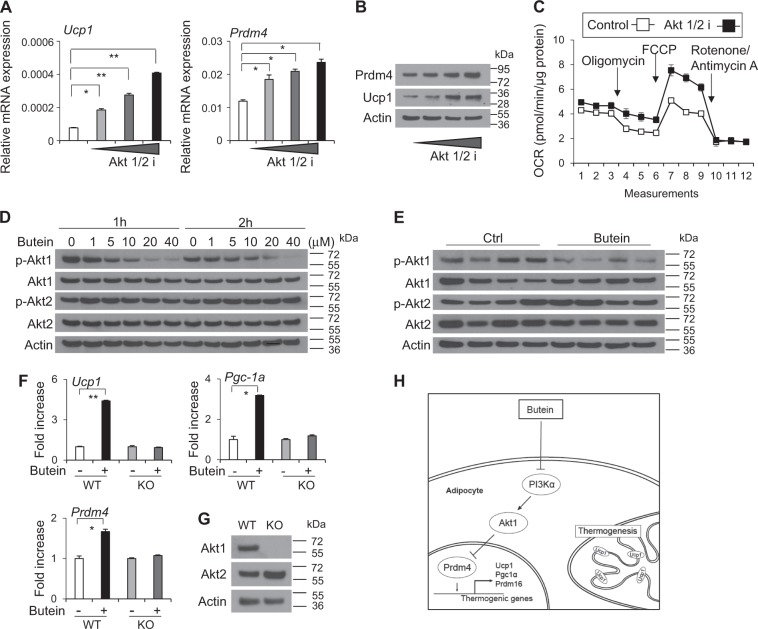
Butein selectively inhibits Akt1 and Akt1 is necessary for effects of butein in adipocytes. **a** C3H10T1/2 adipocytes were treated with 10, 20, 40 μM of Pan Akt inhibitor (Akt1/2 i) for 6 h and expression levels of *Ucp1* and *Prdm4* mRNA were determined by real-time PCR. **b** C3H10T1/2 adipocytes were treated with Akt1/2 i (10, 20, or 40 μM) for 6 h and expression levels of Prdm4 and Ucp1 protein were measured by western blotting. **c** C3H10T1/2 adipocytes were treated with 20 μM of Pan Akt inhibitor (Akt1/2 i) for 6 h and consumption rates (OCR) was measured in approximately 8 min intervals using XF24 Extracellular Flux Analyzer. Data represent means ± s.d. (*n* = 3). **d** C3H10T1/2 adipocytes were treated with butein, and Akt1 and Akt2 phosphorylation levels were determined by western blot analysis. **e** Akt1 and Akt2 phosphorylation levels in epididymal adipose tissue (eWAT) of HFD-fed C57BL/6J mice treated with butein or control for 3 weeks (*n* = 4 per group) were determined by western blot analysis. **f** Mouse embryonic fibroblast isolated from wild-type (WT) mice or Akt1 knockout (KO) mice were treated with DMSO (control) or butein for 12 h and expression of Prdm4 and thermogenic genes were determined. **g** Expression of Akt1 in WT and KO MEF was verified by western blot analysis. **h** Model of adipocyte browning by butein. Butein inhibit the PI3Kα–Akt1 pathway in adipocytes, leading to upregulation of Prdm4 followed by expression of thermogenic genes. Data represent mean ± s.e.m. and statistically significant differences were determined by Student’s *t*-test. *P < 0.05; **P < 0.005

Akt family, a downstream target of PI3K, has been implicated in insulin-mediated effects. It consists of closely related kinases Akt1-3^43^. To further delineate molecular mechanisms, we directly investigated whether butein could affect the activity of Akt. Treatment of C3H10T1/2 adipocytes with pan-Akt inhibitor (Akt1/2 i) increased *Prdm4* and *Ucp1* mRNA and protein expression (Figs. 7a, b). In line with this, oxygen consumption was increased significantly in cells treated with Akt1/2i compared with control cells (Fig. 7c).

Akt1 is ubiquitously expressed in most tissues. Akt2 is highly expressed in insulin-sensitive tissues, whereas Akt3 is selectively expressed in brain, kidney, and heart^43^. To determine the specificity of butein on Akt family, we assessed the effect of butein on Akt1 and Akt2. Treatment of C3H10T1/2 adipocytes with butein decreased phosphorylation of Akt1 in a dose- and time-dependent manner. However, it failed to affect phosphorylation levels of Akt2 (Fig. 7d and Supplementary Figure S22). Selective inhibition on Akt1 but not Akt2 was also observed in eWAT from mice treated with butein (Fig. 7e). Consistently, Akt1 knockdown in adipocytes induced Prdm4 and Ucp1 protein expression and increased mitochondrial mass (Supplementary Figure S23). To further investigate a role of Akt1 in the butein-mediated effects on Prdm4 and thermogenic induction, we assessed the effect of butein in the absence of Akt1 using knockout mouse embryonic fibroblasts (MEF) (Figs. 7f, g). Butein induced *Prdm4* expression in wild-type MEF but this effect was significantly blunted in Akt1 KO MEF. Similarly, butein induced the expression of *Pgc-1a and Ucp1* in wild-type MEF, but not in Akt1 KO MEF (Figs. 7f, g). Taken together, these data demonstrate the necessity of Akt1 in butein-induced Prdm4 and thermogenic gene expression, further highlighting the importance of PI3Kα–Akt1–Prdm4 cascades in the butein-mediated WAT browning (Fig. 7h).

## Discussion

Induction of brown-like adipocytes within WAT can protect rodents against the development of diet-induced obesity and its related metabolic diseases^19,25,44^. Increase of BAT-like activity in WAT in human upon cold acclimation can also increase energy expenditure and reduce body fat mass^8,12,45^. These observations led to the notion that activation of BAT might be considered as a new strategy for counteracting obesity and its associated metabolic derangements. In this study, we showed that butein directly inhibited PI3Kα. PI3Kα-selective inhibitors induced *Prdm4* and *Ucp1* expression in adipocytes. These findings are consistent with prior studies showing that PI3K signaling can affect energy expenditure and metabolism. Pten transgenic mice and PI3K synthetic inhibitors can increase energy expenditure and protect mice against obesity and metabolic disorders^46,47^. However, it has been shown that knockin mutation or adipose-specific deletion of PI3Kα can decrease mitochondrial associated respiration, lower energy balance, and promote metabolic dysfunction^40,41^. Given with PI3K is a nodal point that incorporates various signaling pathways including insulin signaling, PI3K inhibition during development may further complicate actions in obesity and insulin resistance. Indeed, PI3K signaling seems to regulate embryonic adipogenesis and adult obesogenic adipogenesis through different mechanisms^48^. Human clinical trials with small group of cancer patients treated with potent PI3K p110α inhibitors exhibited manageable hyperglycemia in 7 out of 25 cancer patients^49^. A recent study has suggested that pharmacological inhibition of PI3Kα (BYL719) can induce weight loss and increase energy expenditure in genetically induced obese mice^42^. Such complicated and discrepant effects of PI3K signaling in metabolism might be partly due to developmental actions, partial loss, different isoforms in various adipose depots, presence of compensatory/redundant mechanisms, and feeding with different experimental diets that may provide distinct metabolic effects^41,50^. It is thus plausible that selective inhibition of PI3Kα in adult adipose may serve as a strategy to reduce obesity and increase energy expenditure in obesogenic conditions.

Overlapping and unique functions among Akt family have been reported^43^. Akt1 null (Akt1-/-) mice were protected from HFD-induced obesity and insulin resistance through enhanced energy expenditure. Tissue-specific deletion of Akt1 in muscle, brain, and liver does not recapitulate the phenotype of Akt1 whole body knockout mice, indicating that Akt1 in another tissue (s) such as adipose tissue may regulate energy expenditure^51^. In line with this, other studies have shown that BAT-specific conditional deletion of Akt1 significantly reduces body mass and adiposity accompanied with increased Ucp1 expression^52^. Further, DJ-1 a dominant Pten-negative regulator has been shown to decrease Ucp1 expression and energy expenditure through acting on Akt1 (but not Akt2) activation^52^. Consistent with these observations, our results on Akt1 and Akt2 phosphorylation levels by butein and necessity of Akt1 for effects of butein indicate that selective inhibition of Akt1 in adipose tissue can stimulate WAT browning. Unlike Akt1, ablation of Akt2 results in impaired glucose tolerance and glucose uptake, whereas Akt3 deletion causes smaller brain size with normal glucose homeostasis^43^. Together, we speculate that the adipose-selective inhibition of Akt1 is a novel strategy to induce energy expenditure against obesity and its associated metabolic diseases.

Butein treatments in current studies are well-tolerated without showing any notable toxicity. However, a more detailed study of toxicity should be performed because it exerts various biological activities including anti-inflammatory, anticancer, anti-obese, anti-diabetic, and neuroprotective effects possibly through multiple molecular targets^53^. Future experiments of adipose tissue-specific delivery of butein in obese mice are needed to provide safe and effective strategies against obesity and consequent metabolic diseases. Together, our studies showed that chronic inhibition on PI3Kα-Akt1 and its mediated Prdm4 signaling in adipose tissue is a newly identified axis involved in the induction of Ucp1 and thermogenic genes.

In conclusion, treatment with the small molecule butein and adipose-specific induction of Prdm4 prevented obesity and metabolic diseases in HFD-fed mice. We also presented evidence that butein directly inhibited PI3Kα activity and its downstream Akt1 was necessary for butein-mediated induction of thermogenic gene expression. These data suggest that the small molecule butein and its target (PI3Kα–Akt1–Prdm4 pathway) in adipocytes might be useful for developing treatments for obesity and related metabolic diseases.

## Materials and methods

### Cell culture

C3H10T1/2 cells were purchased from the American Type Culture Collection (Manassas, VA, USA) and cultured in Dulbecco’s modified Eagle’s medium (DMEM) (Hyclone, Logan, UT, USA) supplemented with 10% fetal bovine serum (FBS, Hyclone, Logan, UT, USA). Confluent C3H10T1/2 cells were induced to adipocytes using adipogenic medium containing DMEM, 10% FBS, 1 µM dexamethasone (Sigma, St. Louis, MO, USA), IBMX (0.5 mM, Sigma), 1 µM troglitazone (Sigma), and 5 µg/ml insulin (Sigma). The medium was changed every 2 days with fresh media containing DMEM, FBS, insulin, and troglitazone for 8 days. Properly maintained C3H10T1/2 cells showed >80% of differentiation at day 8. Differentiation degree was checked by Oil red O staining followed by microscopic analysis to make sure homogenous differentiation. Cells did not reach the proper rate of differentiation were not used. Human mesenchymal stem cells were purchased from the Lonza (Allendale, NJ, USA) and maintained in DMEM (Hyclone, Logan, UT, USA) supplemented with 10% FBS (Hyclone, Logan, UT, USA). Confluent mesenchymal stem cells were induced to adipocytes by 1 µM dexamethasone (Sigma, St. Louis, MO, USA), 0.5mM 3-isobutyl-1-methylxanthine (Sigma), 1 µM troglitazone (Sigma), and 5 µg/ml insulin (Sigma).

For chemical inhibitor treatments, C3H10T1/2 adipocytes were treated with 20 µM of PKA inhibitor H89 (Sigma), PKC inhibitor Go6983 (Sigma), MAPK kinase inhibitor U0126 (Sigma), PI3K inhibitor Ly294002 (Sigma), or PI3K inhibitor Wortmannin (Sigma). PI3K p110α-selective inhibitors BYL719 and HS-173, β-selective inhibitor GSK2636771, δ-selective inhibitor IC-87114, and γ-selective inhibitor AS-252424 were purchased from Selleckchem (Houston, TX, USA). For PI3K inhibition, PI3K ADP-Glo kinase assay kits (PI3Kα, PI3Kβ, PI3Kδ, and PI3Kγ) were purchased from Promega (Madison, WI, USA) and PI3K ADP-Glo kinase activities were measured according to the manufacturer’s instructions. Prdm4 knockdown was performed as previously described^30^. The PI3Kα siRNA sequences for si1 and si2 were 5ʹ-GAAUGAUAGUGACUUUAGAUU-3ʹ and 5ʹ-GAAUAUCAGGGCAAGUAUAUU-3ʹ, respectively.

For mitochondrial staining, differentiated C3H10T1/2 adipocytes were treated with BYL719 (100 µM), HS-173 (20 µM), Akt1/2 i (20 µM) or transfected with Akt1 siRNA and stained cells with cytopainter for 1 h followed by fixation. Staining was observed by fluorescence microscopy as previously described^30^. OCR was determined using a Seahorse Bioscience XF24 analyzer. C3H10T1/2 cells were differentiated into adipocytes for 6 days and treated with BYL719 (100 µM), HS-173 (20 µM), Akt1/2 i (20 µM). Then, adipocytes were incubated in pre-warmed unbuffered DMEM (sodium bicarbonate free, pH 7.4) for 1 h. Mitochondrial capacities were profiled by treating compounds of oligomycin (2 μg/ml), carbonyl cyanide-p-trifluoromethoxyphenylhydrazone (2 μM), Rotenone (1 μM), and antimycin A (1 μM).

### Expression analysis

To measure mRNA expression levels of genes, total RNA was isolated from adipocytes using TRIzol reagent (Invitrogen, Carlsbad, CA, USA) following the manufacturer’s instructions. Briefly, adipose tissues were homogenized in TRIzol and the lipid layer was removed. Total RNAs were purified using RNA purification Kit (Qiagen, Germantown, MD, USA). Then, complementary DNA was reversely transcribed using RTase M-MLV (2640A, Takara, Otsu, Japan). PCR amplification was performed in a thermal cycler (Takara). Gene-specific primer sets were described previously^29^. Expression was normalized to the level of ribosomal protein 36B4. We also verified the relative expression levels by normalizing other housekeeping genes such as Tbp and actin.

For western blotting, adipocytes or adipose tissues from mice treated with butein were harvested and lysed in RIPA buffer (1 % NP-40, 50 mM Tris-HCl, pH 7.4, 150 mM NaCl, and 10 mM NaF) containing a protease inhibitor cocktail (Roche Diagnostics, Manheim, Germany). Western blot analyses were performed as described previously^54^. Homogenates of adipose tissues (100 mg) in RIPA buffer (200 μl) were centrifuged at 14,000 rpm for 10 min at 4 °C and supernatants were collected. Protein lysates were subjected to sodium dodecyl sulfate polyacrylamide gel electrophoresis and transferred to polyvinylidene difluoride membranes (Bio-Rad Laboratories, Hercules, CA, USA). These membranes were probed with primary antibodies against Prdm4 (ab156867, Abcam, Cambridge, MA, USA), Ucp1 (ab10983, Abcam), Akt (4685s, Cell Signaling, Danvers, MA, USA), p-Akt (4060s, Cell Signaling), Akt1 (75692s, Cell Signaling) p-Akt1 (9018s, Cell Signaling), Akt2 (5239s, Cell Signaling), p-Akt2 (8599s, Cell Signaling), or actin (sc-47778 horseradish peroxidase (HRP), Santa Cruz Biotech, Santa Cruz, CA, USA) followed by incubation with HRP-conjugated secondary antibodies (AbFrontier). An enhanced chemiluminescent western blotting detection reagent (GE Health Care, MA, USA) was used to detect protein expression.

### Animal studies

All animal studies were carried out in accordance with the guidelines of the Animal Research Committee of Sungkyunkwan University or Animal Research Committee of University of Utah. Seven weeks old male C57BL/6J mice were purchased from Central Lab Animal Inc. (Seoul, Korea) and housed in rooms with the ambient temperature (23 °C). For butein treatment in obese mice, mice (*n* = 5) in one group were fed a LFD (D12450J, Research Diets Inc., New Brunswick, NJ, USA). Mice in the other three groups (*n* = 7 per group) were fed a HFD (D12492, Research Diets Inc.). Two groups of mice were i.p. injected with butein either at 5 mg/kg per day (HFD + butein 5 mg/kg, *n* = 7) or at 15 mg/kg per day (HFD + butein 15 mg/kg, *n* = 7). The other group was administrated vehicle as a control (HFD, *n* = 7) for 8 weeks. After treatments of butein (15 mg/kg) for 8 weeks, the mice were imaged using Micro-CT (Skyscan model 1076; Skyscan, Kontich, Belgium) under anesthesia. The resolution of the micro-CT was 35 μm. Seven weeks old male C57BL/6J mice, housed at 30 °C, were subjected to high‐fat diet and injected with control (HFD, *n* = 7) or butein at 15 mg/kg per day (*n* = 7). The body weight and food intake were measured twice per week.

Whole-body energy metabolism was measured using Columbus Instruments Oxymax Lab Animal Monitoring System. C57BL/6J mice on a HFD (Research Diets Inc.) were treated with butein (15 mg/kg) via i.p. injection for 3 weeks and energy expenditure was evaluated. Mice were placed in metabolic cages and were acclimated in the metabolic chambers for 1 day before the measuring energy expenditure, O_2_ consumption, and CO_2_ production.

Transgenic mice were generated from University of Utah transgenic core facility. Briefly, aP2-Prdm4 transgenic plasmid was linearized with Kpn I and purified using a DNA purification kit (Qiagen). Transgenic founders were produced by nuclear injection of the linearized DNA into C57Bl6 X DBA mice. Tail DNA was harvested from pups and founders were identified by PCR using primers specific for transgene. The primer sequences were 5′-GGGGAAGTTCAATGCATTAGC-3′ and 5′-GTGATGTGTGGGCTACCTG-3′ or 5′-ATTGCCAGGGAGAACCAAAGT-3′ and 5′-AAGCCCAGGTGACTTCCT-3′. Founders were crossed with C57Bl6 mice to generate mixed progeny and were used in the experiments. Determination of energy expenditure by Columbus Instruments Oxymax Lab Animal Monitoring System and body composition by NMR were performed at the metabolic phenotyping core of the University of Utah. Transgenic experimentation was performed according to guidelines established by the NIH and protocol were approved by the Animal Care and Use Committee of the University of Utah.

To perform glucose tolerance tests, mice were fasted for 6–16 h and tail-vein blood samples were collected after i.p. injection of glucose (1 g/kg). Blood glucose levels were then determined. For insulin tolerance tests, mice were injected i.p. with insulin (Humulin R, Eli Lilly) (0.5 U/kg) and their glucose levels were determined.

### Statistical analysis

Data are presented as mean ± standard error of the mean (s.e.m) or standard deviation (s.d). Statistical tests included two-way analysis of variance (ANOVA) and unpaired *t*-test. Statistical analysis was performed using GraphPad Prism (GraphPad Software, Inc., La Jolla, CA, USA). Statistical significance was defined at *P* *<* 0.05.

## Electronic supplementary material

Supplemental figures1–23

Supplementary Figure Legends
