# Supplementary material for: PI3Ka-Akt1-mediated Prdm4 induction in adipose tissue increases energy expenditure, inhibits weight gain, and improves insulin resistance in diet-induced obese mice
Source: Cell Death Dis. 2018 Aug 29;9(9):876. doi: 10.1038/s41419-018-0904-3 (PMC6115456; doi:10.1038/s41419-018-0904-3)
Supplement: Supplementary file 1 — Supplemental figures1–23 [file 41419_2018_904_MOESM1_ESM.pptx]

## Slide 1
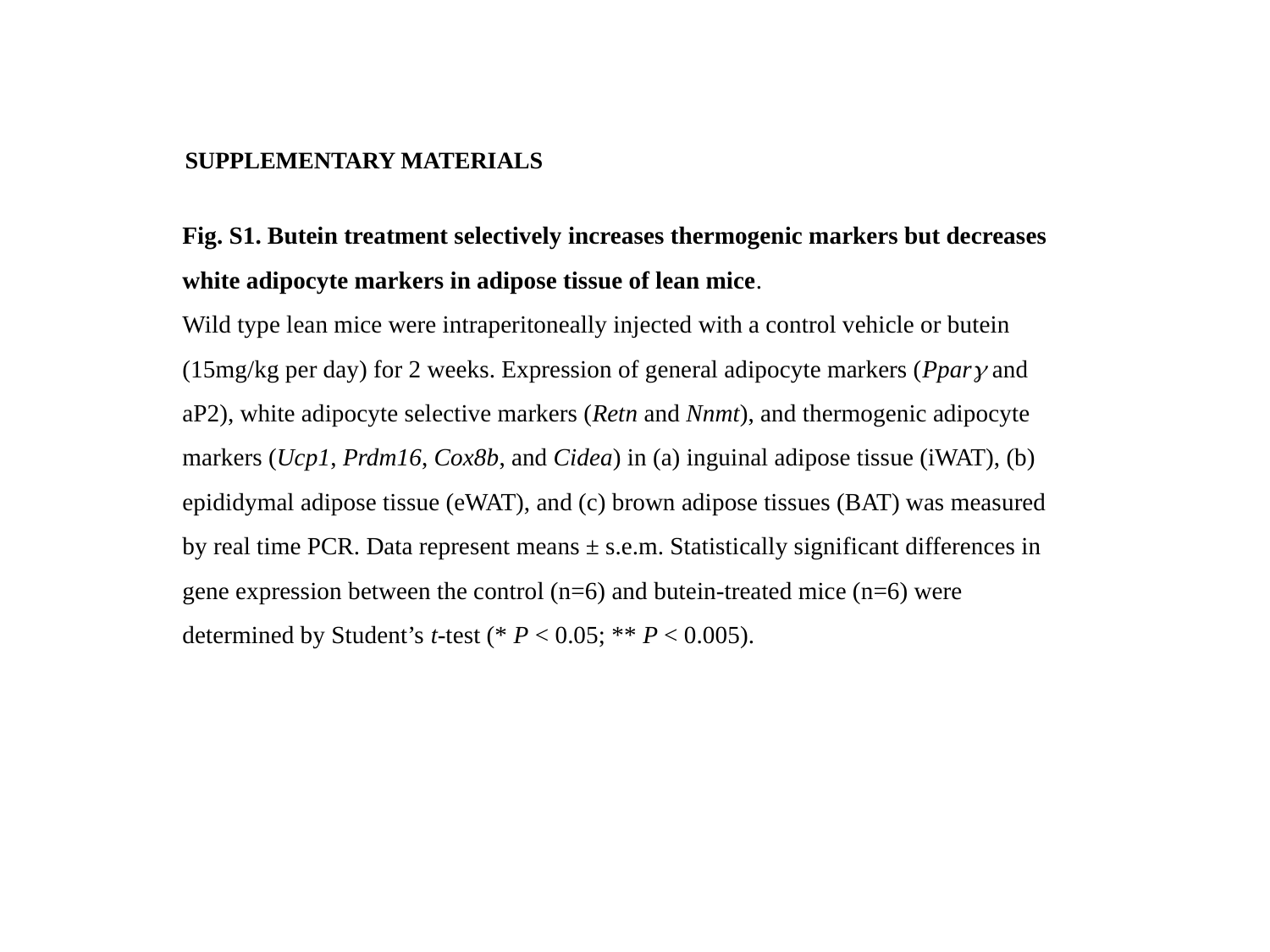

SUPPLEMENTARY MATERIALS
Fig. S1. Butein treatment selectively increases thermogenic markers but decreases white adipocyte markers in adipose tissue of lean mice.
Wild type lean mice were intraperitoneally injected with a control vehicle or butein (15mg/kg per day) for 2 weeks. Expression of general adipocyte markers (Pparγ and aP2), white adipocyte selective markers (Retn and Nnmt), and thermogenic adipocyte markers (Ucp1, Prdm16, Cox8b, and Cidea) in (a) inguinal adipose tissue (iWAT), (b) epididymal adipose tissue (eWAT), and (c) brown adipose tissues (BAT) was measured by real time PCR. Data represent means ± s.e.m. Statistically significant differences in gene expression between the control (n=6) and butein-treated mice (n=6) were determined by Student’s t-test (* P < 0.05; ** P < 0.005).

## Slide 2
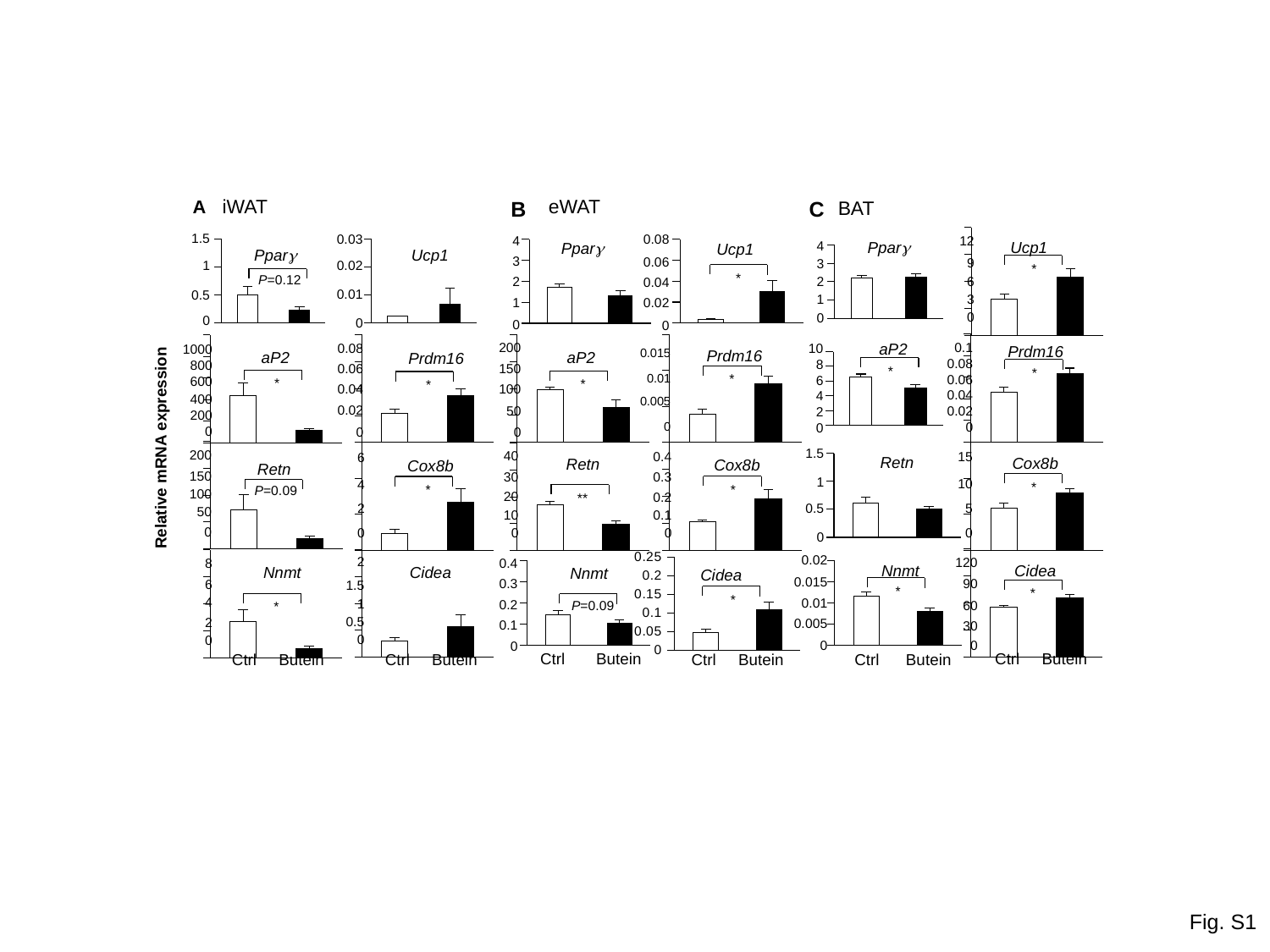

iWAT
eWAT
B
A
BAT
C
1.5
 1
0.5
 0
### Chart
| Category | |
|---|---|Pparγ
0.03
0.02
0.01
 0
### Chart
| Category | |
|---|---|Ucp1
0.08
0.06
0.04
0.02
 0
Ucp1
### Chart
| Category | |
|---|---|
### Chart
| Category | |
|---|---|12
 9
 6
 3
 0
Ucp1
### Chart
| Category | |
|---|---|Pparγ
4
3
2
1
0
4
3
2
1
0
Pparγ
### Chart
| Category | |
|---|---|*
*
P=0.12
### Chart
| Category | |
|---|---| 0.1
 0.08
 0.06
 0.04
 0.02
 0
Prdm16
### Chart
| Category | |
|---|---|aP2
10
 8
 6
 4
 2
 0
### Chart
| Category | |
|---|---|200
150
100
 50
 0
aP2
### Chart
| Category | |
|---|---| 0.015
 0.01
0.005
 0
Prdm16
### Chart
| Category | |
|---|---|0.08
0.06
0.04
0.02
 0
Prdm16
### Chart
| Category | |
|---|---|1000
 800
 600
 400
 200
 0
aP2
Relative mRNA expression
*
*
*
*
*
*
1.5
 1
0.5
 0
Retn
### Chart
| Category | |
|---|---|
### Chart
| Category | |
|---|---|200
150
100
 50
 0
Retn
### Chart
| Category | |
|---|---|15
10
 5
 0
Cox8b
### Chart
| Category | |
|---|---|6
4
2
0
Cox8b
### Chart
| Category | |
|---|---|0.4
0.3
0.2
0.1
 0
Cox8b
### Chart
| Category | |
|---|---|40
30
20
10
 0
Retn
*
*
*
P=0.09
**
 0.02
0.015
 0.01
0.005
 0
Nnmt
### Chart
| Category | |
|---|---|
### Chart
| Category | |
|---|---|120
 90
 60
 30
 0
Cidea
 2
1.5
 1
0.5
 0
### Chart
| Category | |
|---|---|Cidea
### Chart
| Category | |
|---|---|Cidea
### Chart
| Category | |
|---|---|8
6
4
2
0
Nnmt
0.4
0.3
0.2
0.1
 0
### Chart
| Category | |
|---|---|Nnmt
*
*
*
P=0.09
*
Ctrl Butein
Ctrl Butein
Ctrl Butein
Ctrl Butein
Ctrl Butein
Ctrl Butein
Fig. S1

## Slide 3
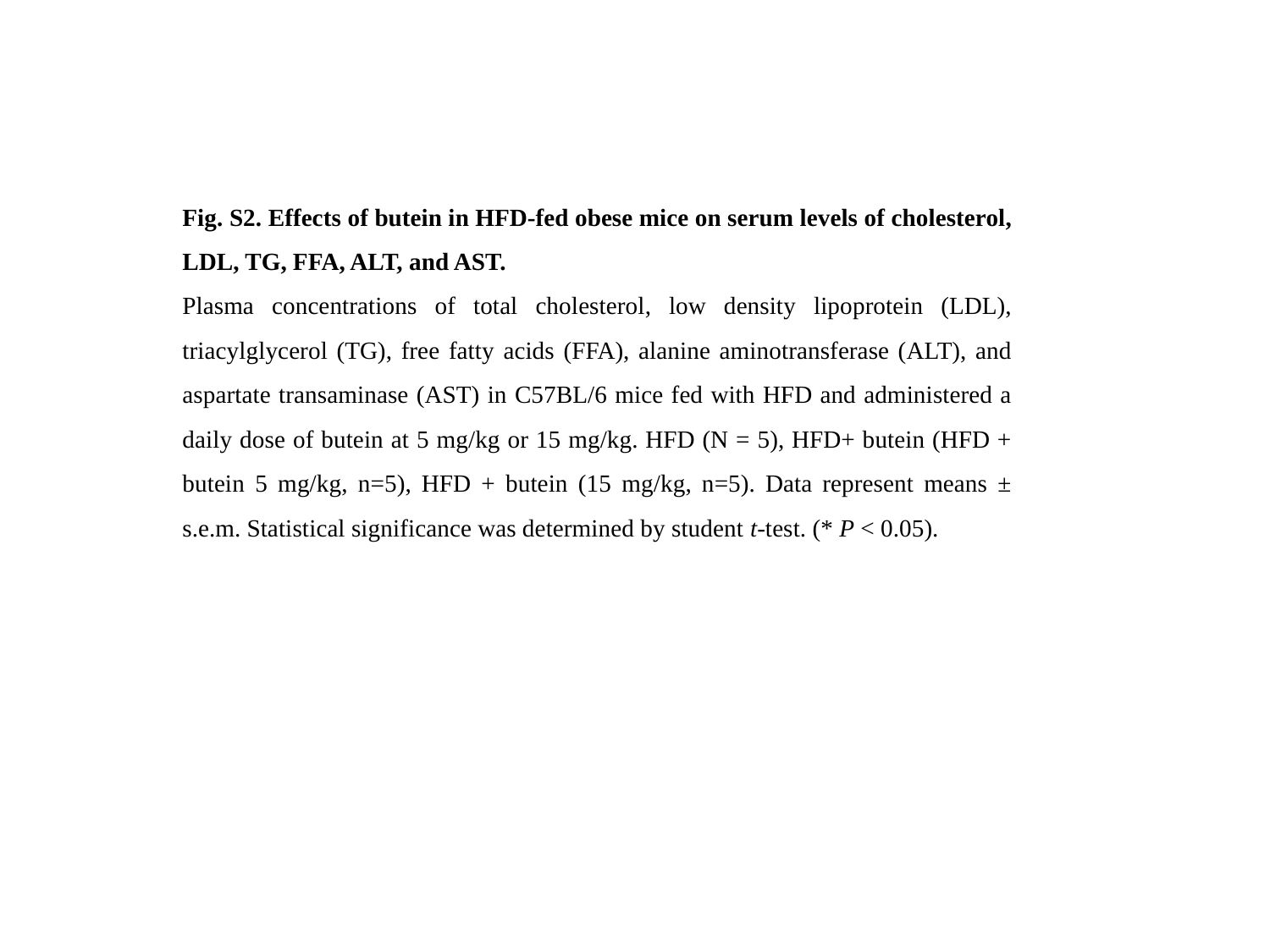

Fig. S2. Effects of butein in HFD-fed obese mice on serum levels of cholesterol, LDL, TG, FFA, ALT, and AST.
Plasma concentrations of total cholesterol, low density lipoprotein (LDL), triacylglycerol (TG), free fatty acids (FFA), alanine aminotransferase (ALT), and aspartate transaminase (AST) in C57BL/6 mice fed with HFD and administered a daily dose of butein at 5 mg/kg or 15 mg/kg. HFD (N = 5), HFD+ butein (HFD + butein 5 mg/kg, n=5), HFD + butein (15 mg/kg, n=5). Data represent means ± s.e.m. Statistical significance was determined by student t-test. (* P < 0.05).

## Slide 4
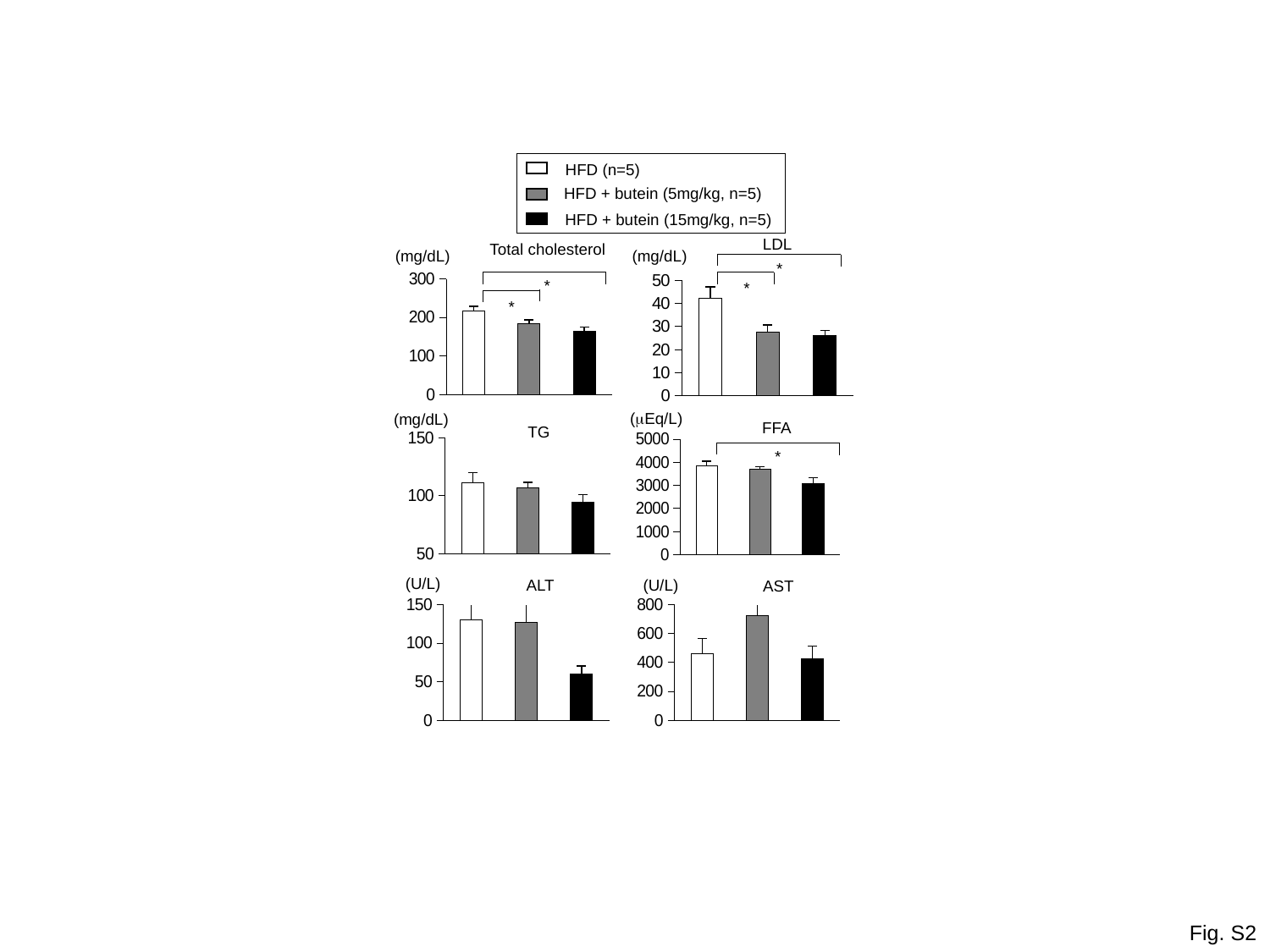

HFD (n=5)
HFD + butein (5mg/kg, n=5)
HFD + butein (15mg/kg, n=5)
LDL
Total cholesterol
(mg/dL)
(mg/dL)
*
### Chart
| Category | |
|---|---|
| HFD | 217.47692307692304 |
| But10 | 182.9846153846154 |
| but30 | 164.8615384615385 |*
### Chart
| Category | |
|---|---|
| HFD | 41.975384615384144 |
| But10 | 27.7107692307685 |
| but30 | 26.24923076923077 |*
*
(μEq/L)
(mg/dL)
FFA
TG
### Chart
| Category | |
|---|---|
| HFD | 111.07692307692308 |
| But10 | 107.17948717948252 |
| but30 | 94.51282051281976 |
### Chart
| Category | |
|---|---|
| HFD | 3847.353846153846 |
| But10 | 3708.6538461538457 |
| but30 | 3090.423076923077 |*
(U/L)
ALT
(U/L)
AST
### Chart
| Category | |
|---|---|
| HFD | 130.36923076923077 |
| But10 | 126.4230769230769 |
| but30 | 59.63076923076923 |
### Chart
| Category | |
|---|---|
| HFD | 461.84615384615364 |
| But10 | 725.8974358974355 |
| but30 | 423.2615384615255 |Fig. S2

## Slide 5
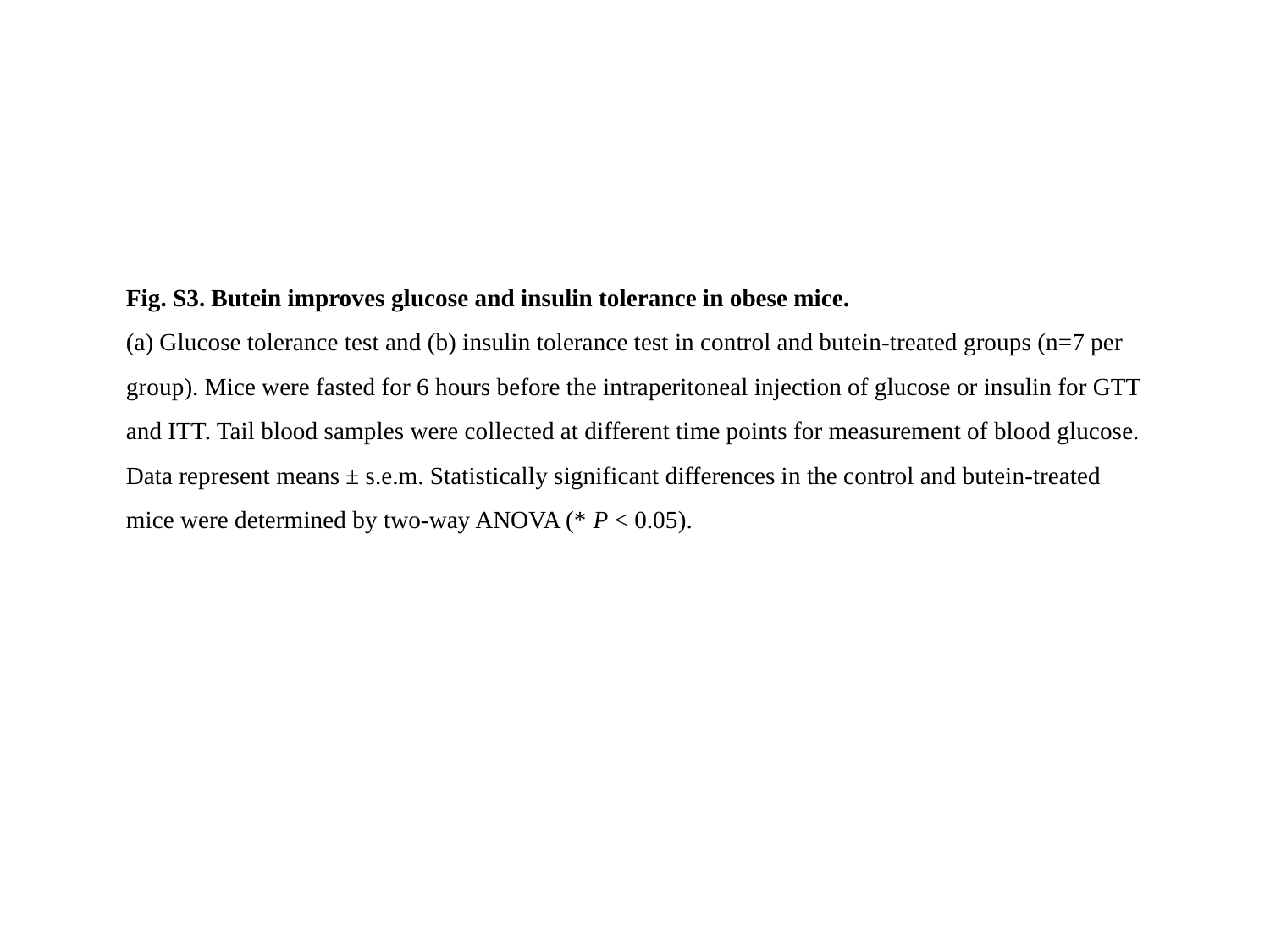

Fig. S3. Butein improves glucose and insulin tolerance in obese mice.
(a) Glucose tolerance test and (b) insulin tolerance test in control and butein-treated groups (n=7 per group). Mice were fasted for 6 hours before the intraperitoneal injection of glucose or insulin for GTT and ITT. Tail blood samples were collected at different time points for measurement of blood glucose. Data r­­epresent means ± s.e.m. Statistically significant differences in the control and butein-treated mice were determined by two-way ANOVA (* P < 0.05).

## Slide 6
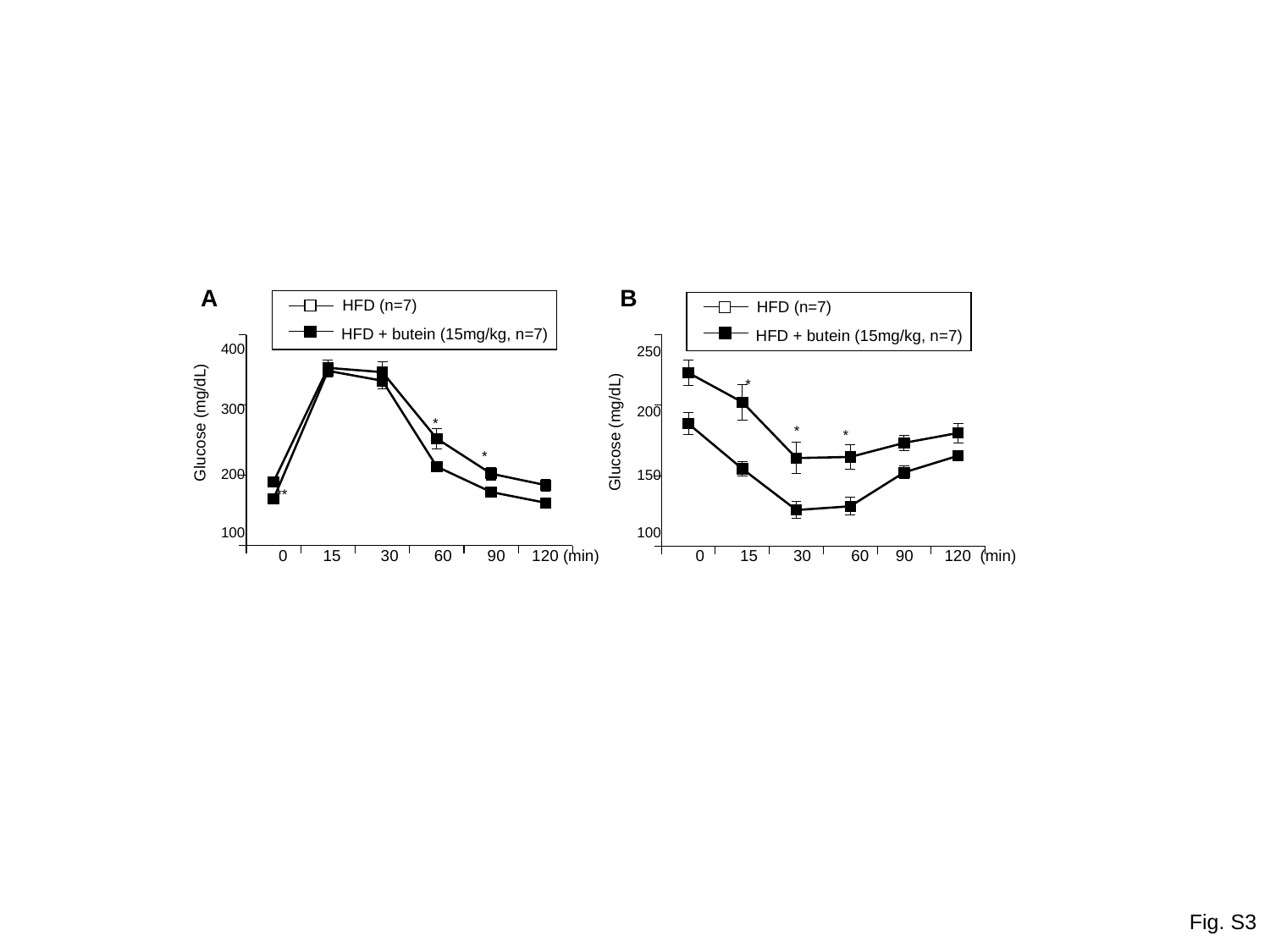

A
B
HFD (n=7)
HFD (n=7)
HFD + butein (15mg/kg, n=7)
HFD + butein (15mg/kg, n=7)
### Chart
| Category | hfd | hfd but 30 |
|---|---|---|
### Chart
| Category | hfd | hfd but 30 |
|---|---|---|400
300
200
100
250
200
150
100
*
*
*
0 15 30 60 90 120 (min)
Glucose (mg/dL)
*
Glucose (mg/dL)
*
**
0 15 30 60 90 120 (min)
Fig. S3

## Slide 7
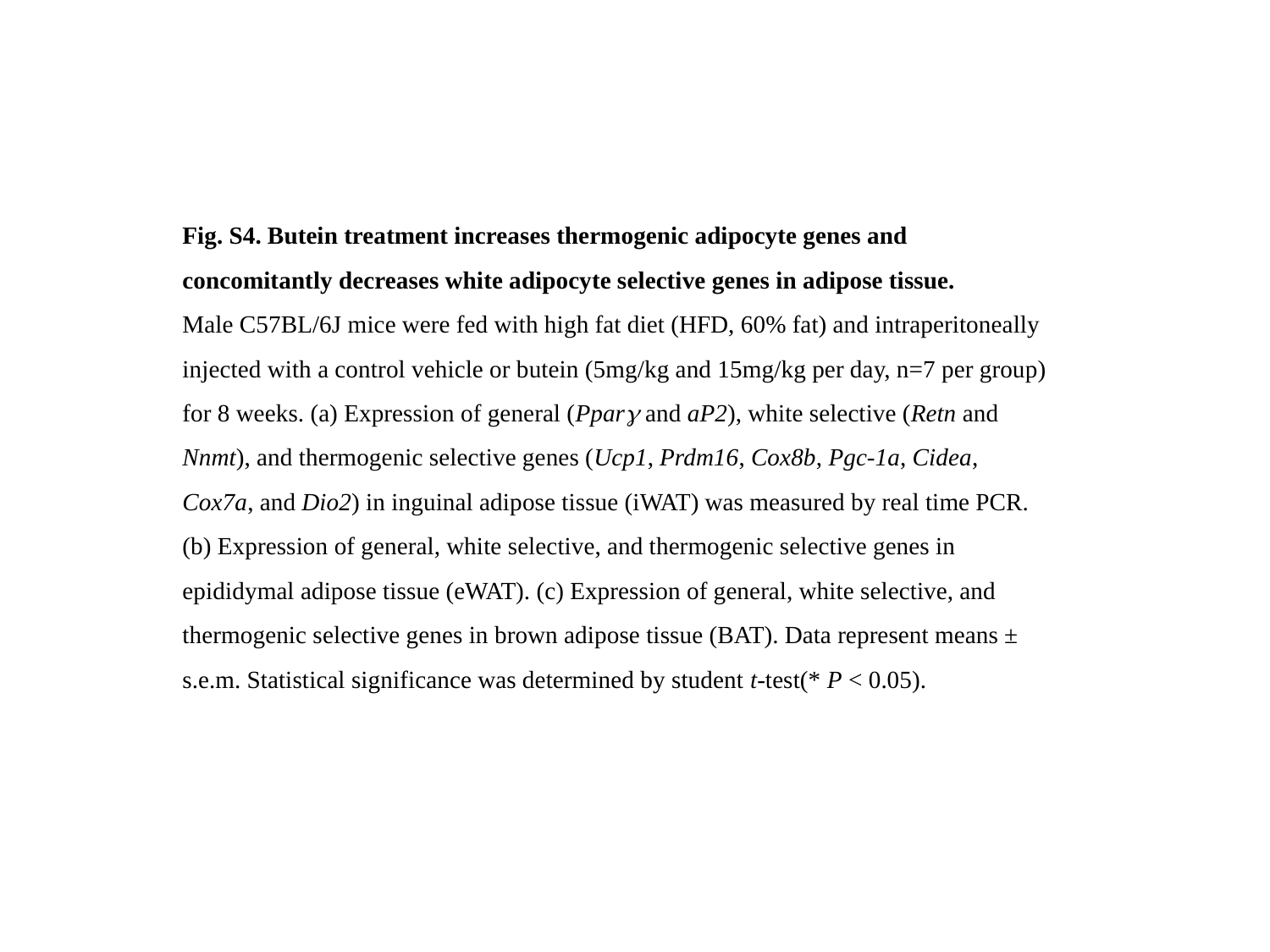

Fig. S4. Butein treatment increases thermogenic adipocyte genes and concomitantly decreases white adipocyte selective genes in adipose tissue.
Male C57BL/6J mice were fed with high fat diet (HFD, 60% fat) and intraperitoneally injected with a control vehicle or butein (5mg/kg and 15mg/kg per day, n=7 per group) for 8 weeks. (a) Expression of general (Pparγ and aP2), white selective (Retn and Nnmt), and thermogenic selective genes (Ucp1, Prdm16, Cox8b, Pgc-1a, Cidea, Cox7a, and Dio2) in inguinal adipose tissue (iWAT) was measured by real time PCR. (b) Expression of general, white selective, and thermogenic selective genes in epididymal adipose tissue (eWAT). (c) Expression of general, white selective, and thermogenic selective genes in brown adipose tissue (BAT). Data represent means ± s.e.m. Statistical significance was determined by student t-test(* P < 0.05).

## Slide 8
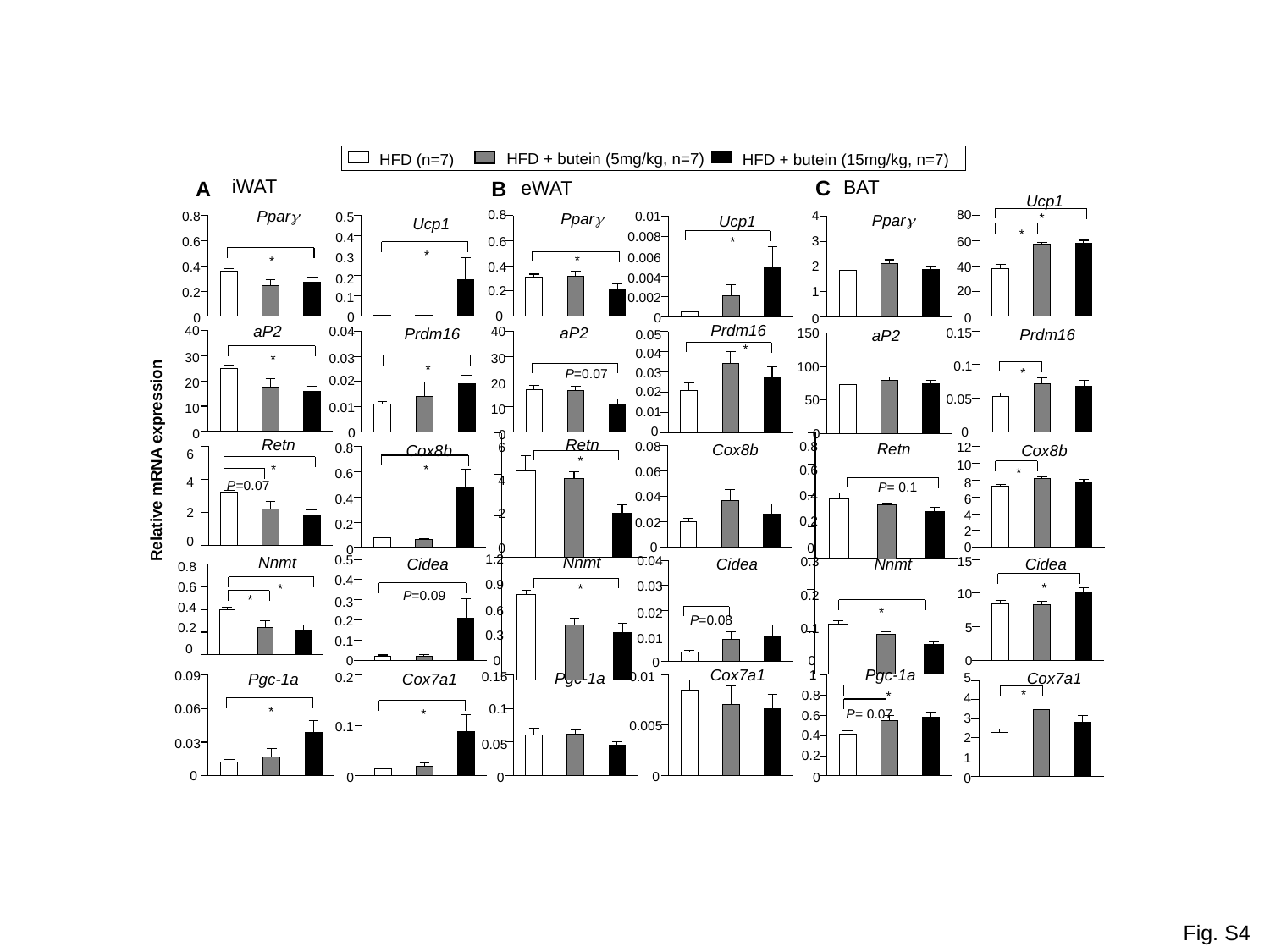

HFD + butein (5mg/kg, n=7)
HFD + butein (15mg/kg, n=7)
HFD (n=7)
iWAT
BAT
C
eWAT
B
A
Ucp1
Pparγ
80
60
40
20
 0
### Chart
| Category | |
|---|---|0.8
0.6
0.4
0.2
 0
4
3
2
1
0
### Chart
| Category | |
|---|---| 0.01
 0.008
 0.006
 0.004
 0.002
 0
0.8
0.6
0.4
0.2
 0
### Chart
| Category | |
|---|---|Pparγ
0.5
0.4
0.3
0.2
0.1
 0
### Chart
| Category | |
|---|---|*
Pparγ
Ucp1
Ucp1
### Chart
| Category | |
|---|---|
### Chart
| Category | |
|---|---|*
*
*
*
*
Prdm16
aP2
40
30
20
10
 0
aP2
0.04
0.03
0.02
0.01
 0
### Chart
| Category | |
|---|---|40
30
20
10
 0
Prdm16
0.15
 0.1
0.05
 0
### Chart
| Category | |
|---|---|Prdm16
150
100
 50
 0
### Chart
| Category | |
|---|---|aP2
0.05
0.04
0.03
0.02
0.01
 0
### Chart
| Category | |
|---|---|
### Chart
| Category | |
|---|---|
### Chart
| Category | |
|---|---|*
*
*
Relative mRNA expression
*
P=0.07
Retn
Retn
### Chart
| Category | |
|---|---|0.8
0.6
0.4
0.2
 0
### Chart
| Category | |
|---|---|6
4
2
0
0.08
0.06
0.04
0.02
 0
12
10
 8
 6
 4
 2
 0
### Chart
| Category | |
|---|---|Retn
 0.8
 0.6
 0.4
 0.2
 0
### Chart
| Category | |
|---|---|Cox8b
### Chart
| Category | |
|---|---|6
4
2
0
Cox8b
Cox8b
### Chart
| Category | |
|---|---|*
*
P=0.07
*
*
P= 0.1
1.2
0.9
0.6
0.3
 0
### Chart
| Category | |
|---|---|
### Chart
| Category | |
|---|---|0.8
0.6
0.4
0.2
 0
### Chart
| Category | |
|---|---|0.3
0.2
0.1
 0
0.5
0.4
0.3
0.2
0.1
 0
### Chart
| Category | |
|---|---|Nnmt
Nnmt
0.04
0.03
0.02
0.01
 0
### Chart
| Category | |
|---|---|15
10
 5
 0
### Chart
| Category | |
|---|---|Cidea
Nnmt
Cidea
Cidea
*
*
*
P=0.09
*
*
P=0.08
Pgc-1a
 1
 0.8
 0.6
 0.4
 0.2
 0
### Chart
| Category | |
|---|---|*
P= 0.07
Cox7a1
0.09
0.06
0.03
 0
### Chart
| Category | |
|---|---|Cox7a1
Pgc-1a
0.15
 0.1
0.05
 0
### Chart
| Category | |
|---|---|0.01
0.005
 0
5
4
3
2
1
0
Cox7a1
Pgc-1a
0.2
0.1
 0
### Chart
| Category | |
|---|---|
### Chart
| Category | |
|---|---|
### Chart
| Category | |
|---|---|*
*
*
Fig. S4

## Slide 9
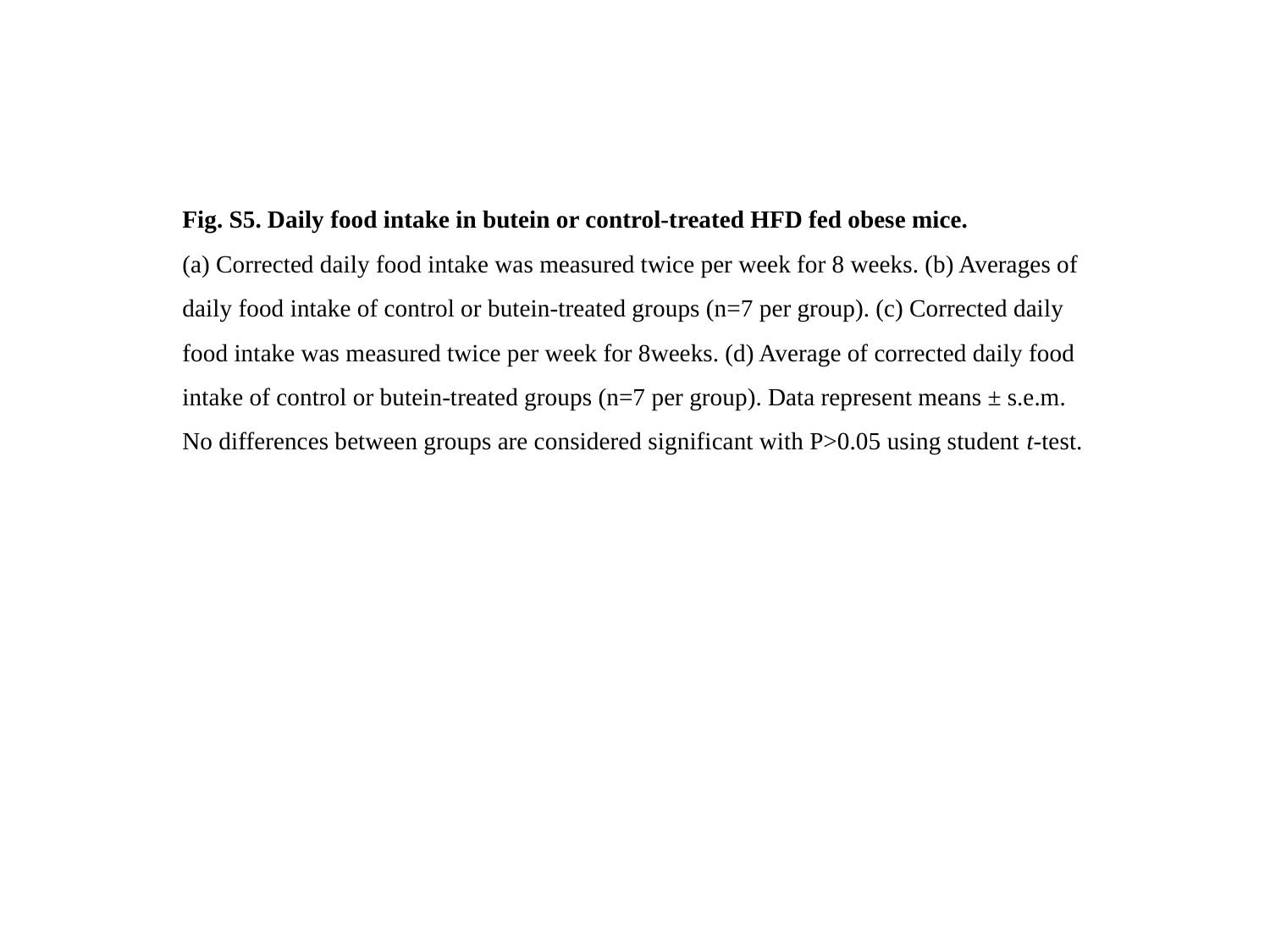

Fig. S5. Daily food intake in butein or control-treated HFD fed obese mice.
(a) Corrected daily food intake was measured twice per week for 8 weeks. (b) Averages of daily food intake of control or butein-treated groups (n=7 per group). (c) Corrected daily food intake was measured twice per week for 8weeks. (d) Average of corrected daily food intake of control or butein-treated groups (n=7 per group). Data represent means ± s.e.m. No differences between groups are considered significant with P>0.05 using student t-test.

## Slide 10
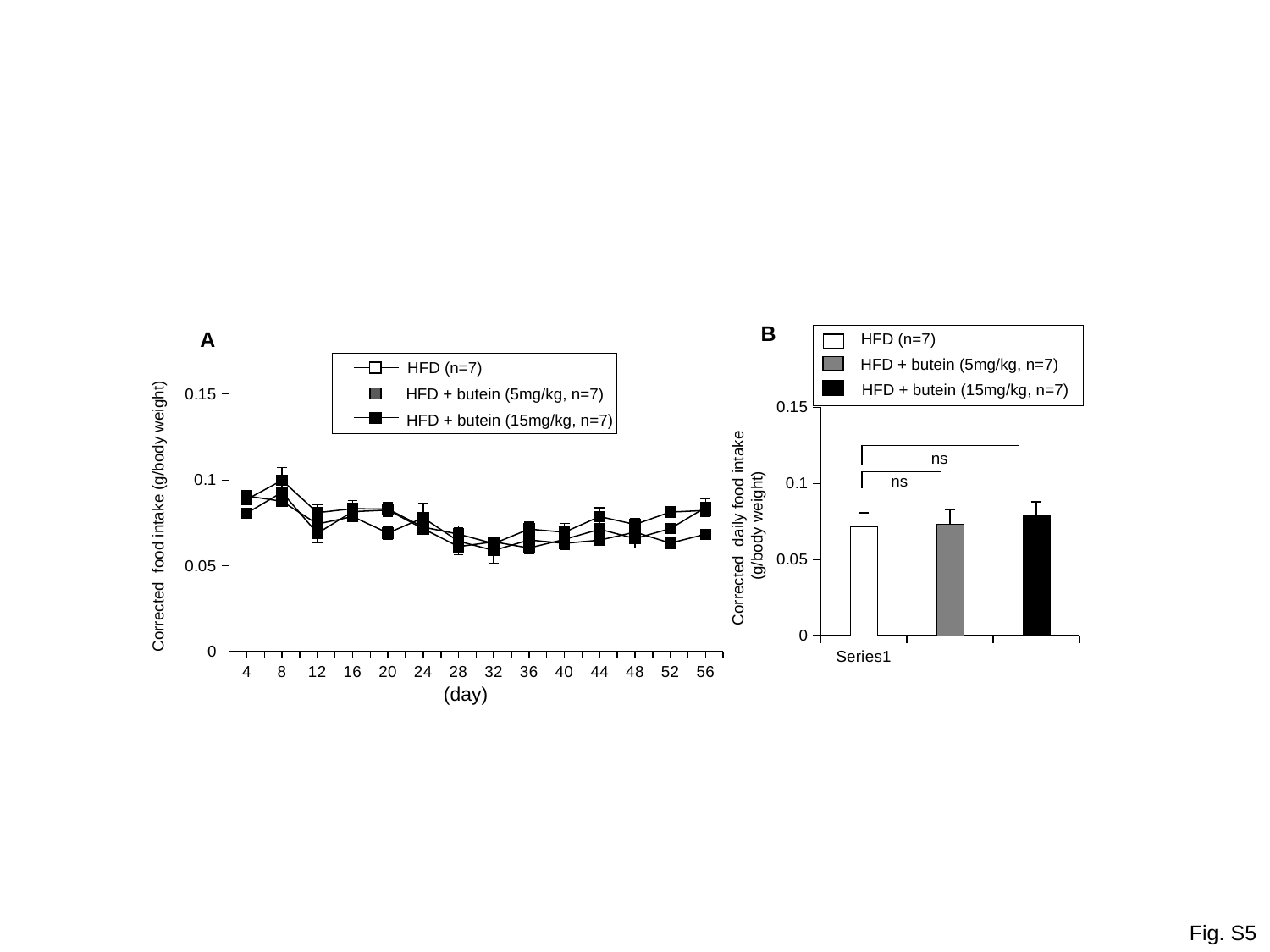

B
A
HFD (n=7)
HFD + butein (5mg/kg, n=7)
HFD (n=7)
HFD + butein (15mg/kg, n=7)
HFD + butein (5mg/kg, n=7)
### Chart
| Category | | | |
|---|---|---|---|
| 4 | 0.090732988107036 | 0.08072304609931046 | 0.08871763699773307 |
| 8 | 0.08759592499926554 | 0.0928622949945901 | 0.09982394585707864 |
| 12 | 0.07439279891456031 | 0.06897589709833538 | 0.08101257765784001 |
| 16 | 0.07871095991930696 | 0.08160226994142472 | 0.08329315359911578 |
| 20 | 0.06914923970814638 | 0.0825252370485933 | 0.08311433241222531 |
| 24 | 0.07802028925266949 | 0.07163516485661733 | 0.07261390135242721 |
| 28 | 0.0643336401462559 | 0.06121510175666149 | 0.0684685847746195 |
| 32 | 0.05901039726981729 | 0.06398172670112953 | 0.06297437459217521 |
| 36 | 0.0650588800282155 | 0.060414252809422475 | 0.07142787296331707 |
| 40 | 0.06310586350644508 | 0.0654275447085392 | 0.0696340647717147 |
| 44 | 0.06499003298388775 | 0.07135690915101807 | 0.07873330875846585 |
| 48 | 0.06968910023360421 | 0.06589582971740324 | 0.07413670298431338 |
| 52 | 0.06325754943459184 | 0.07171246900478613 | 0.08136891955433621 |
| 56 | 0.06844084171970853 | 0.08404918455811106 | 0.08229300150877036 |Corrected food intake (g/body weight)
### Chart
| Category | |
|---|---|
| | 0.07117775044453648 |
| | 0.07302692346042447 |
| | 0.07840088412743804 |HFD + butein (15mg/kg, n=7)
Corrected daily food intake
 (g/body weight)
ns
ns
(day)
Fig. S5

## Slide 11
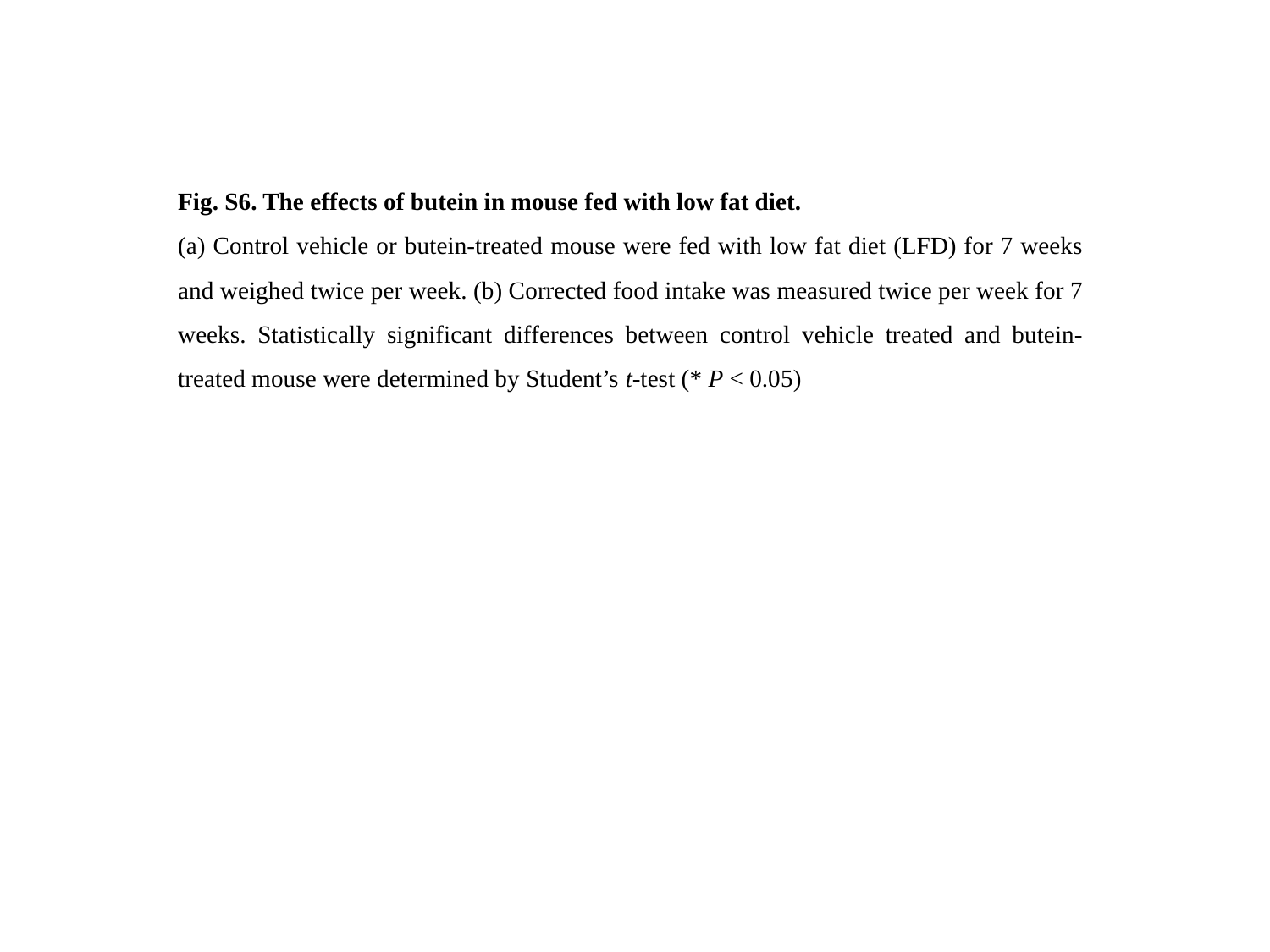

Fig. S6. The effects of butein in mouse fed with low fat diet.
(a) Control vehicle or butein-treated mouse were fed with low fat diet (LFD) for 7 weeks and weighed twice per week. (b) Corrected food intake was measured twice per week for 7 weeks. Statistically significant differences between control vehicle treated and butein-treated mouse were determined by Student’s t-test (* P < 0.05)

## Slide 12
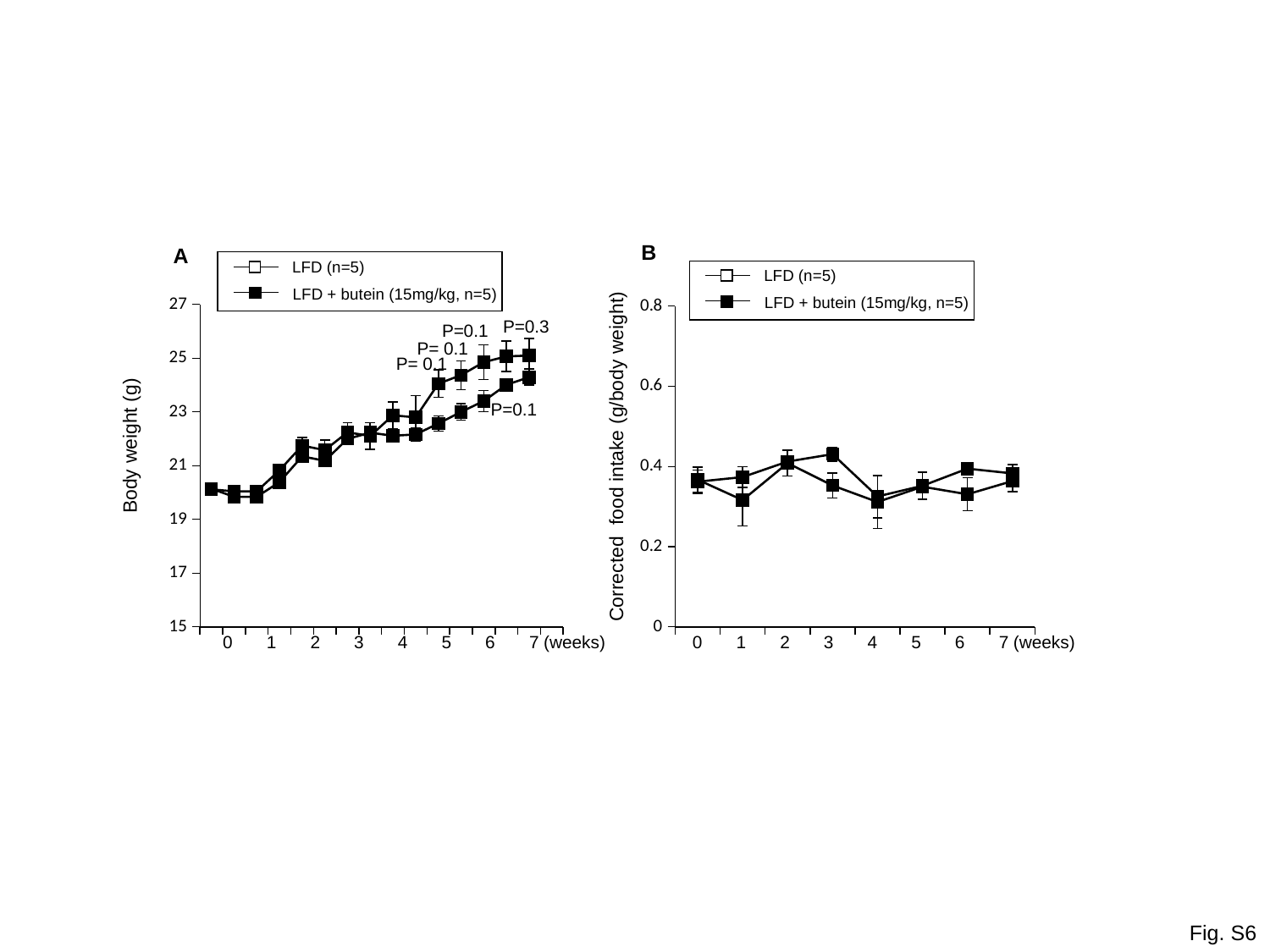

B
A
LFD (n=5)
LFD (n=5)
LFD + butein (15mg/kg, n=5)
LFD + butein (15mg/kg, n=5)
### Chart
| Category | | |
|---|---|---|Corrected food intake (g/body weight)
### Chart
| Category | | |
|---|---|---|
P=0.3
P=0.1
P= 0.1
P= 0.1
Body weight (g)
P=0.1
0 1 2 3 4 5 6 7 (weeks)
0 1 2 3 4 5 6 7 (weeks)
Fig. S6

## Slide 13
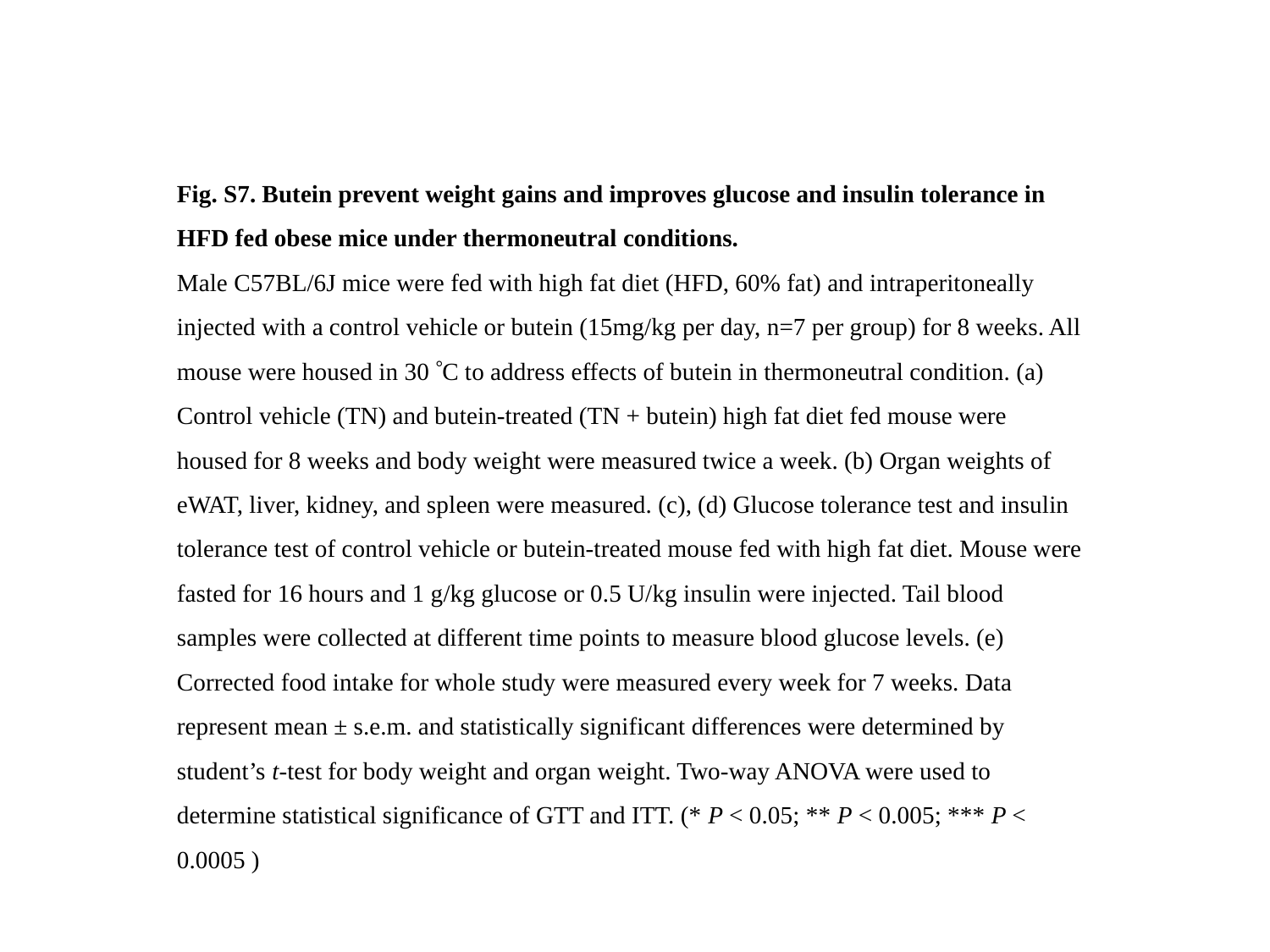

Fig. S7. Butein prevent weight gains and improves glucose and insulin tolerance in HFD fed obese mice under thermoneutral conditions.
Male C57BL/6J mice were fed with high fat diet (HFD, 60% fat) and intraperitoneally injected with a control vehicle or butein (15mg/kg per day, n=7 per group) for 8 weeks. All mouse were housed in 30 °C to address effects of butein in thermoneutral condition. (a) Control vehicle (TN) and butein-treated (TN + butein) high fat diet fed mouse were housed for 8 weeks and body weight were measured twice a week. (b) Organ weights of eWAT, liver, kidney, and spleen were measured. (c), (d) Glucose tolerance test and insulin tolerance test of control vehicle or butein-treated mouse fed with high fat diet. Mouse were fasted for 16 hours and 1 g/kg glucose or 0.5 U/kg insulin were injected. Tail blood samples were collected at different time points to measure blood glucose levels. (e) Corrected food intake for whole study were measured every week for 7 weeks. Data represent mean ± s.e.m. and statistically significant differences were determined by student’s t-test for body weight and organ weight. Two-way ANOVA were used to determine statistical significance of GTT and ITT. (* P < 0.05; ** P < 0.005; *** P < 0.0005 )

## Slide 14
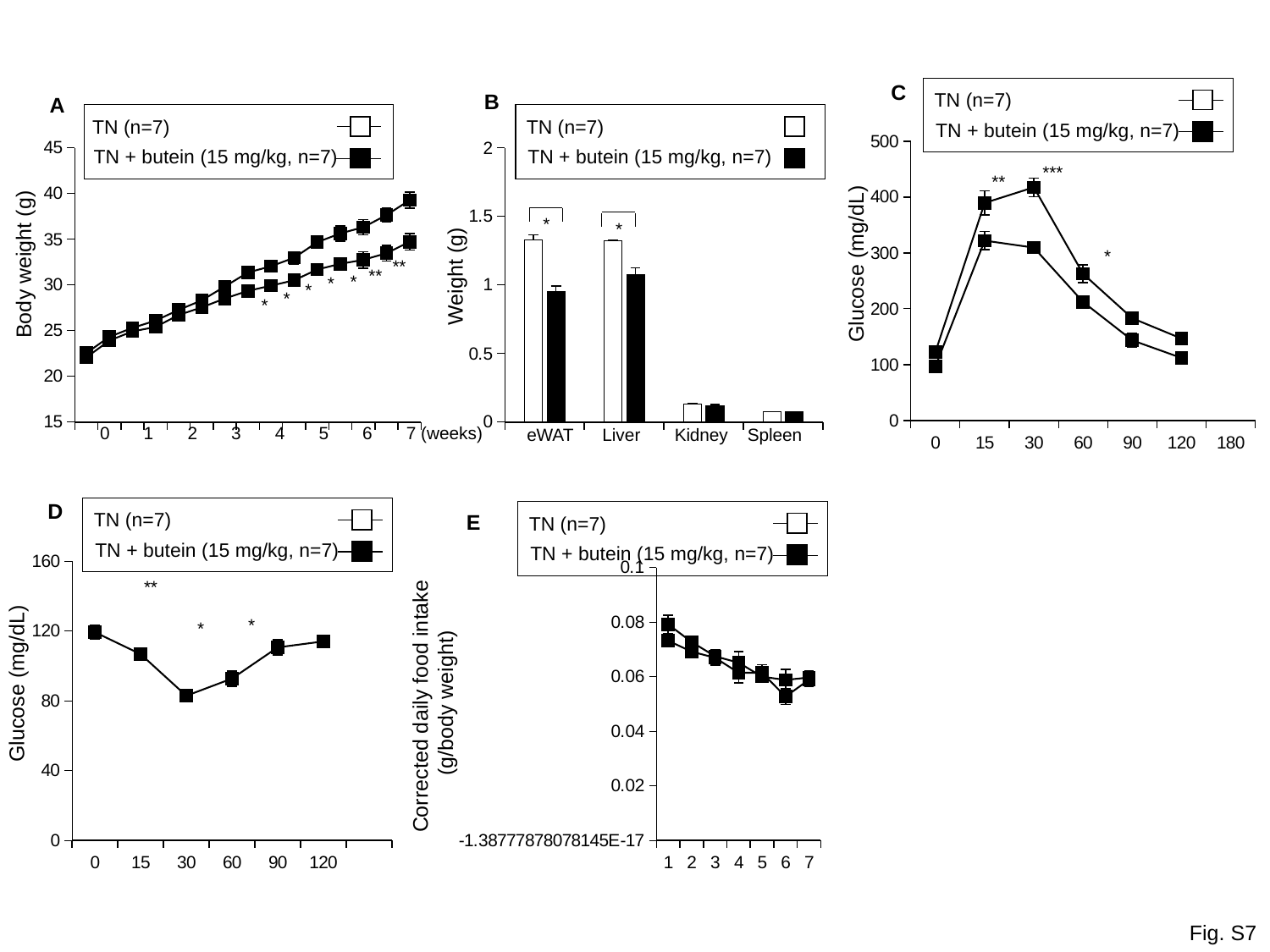

C
TN (n=7)
B
A
TN (n=7)
TN (n=7)
TN + butein (15 mg/kg, n=7)
[unsupported chart]
### Chart
| Category | | |
|---|---|---|
### Chart
| Category | | |
|---|---|---|TN + butein (15 mg/kg, n=7)
TN + butein (15 mg/kg, n=7)
***
**
Body weight (g)
*
*
Weight (g)
*
Glucose (mg/dL)
**
**
*
*
*
*
*
0 1 2 3 4 5 6 7 (weeks)
eWAT Liver Kidney Spleen
D
TN (n=7)
E
TN (n=7)
TN + butein (15 mg/kg, n=7)
TN + butein (15 mg/kg, n=7)
### Chart
| Category | TN_HFD | TN_Butein |
|---|---|---|
| 0 | 131.28571428571428 | 119.28571428571429 |
| 15 | 139.42857142857142 | 106.71428571428571 |
| 30 | 111.14285714285714 | 82.85714285714286 |
| 60 | 115.57142857142857 | 92.71428571428571 |
| 90 | 127.28571428571429 | 110.57142857142857 |
| 120 | 123.57142857142857 | 114.0 |
### Chart
| Category | TN_HFD | TN_Butein |
|---|---|---|**
*
*
Glucose (mg/dL)
Corrected daily food intake
 (g/body weight)
Fig. S7

## Slide 15
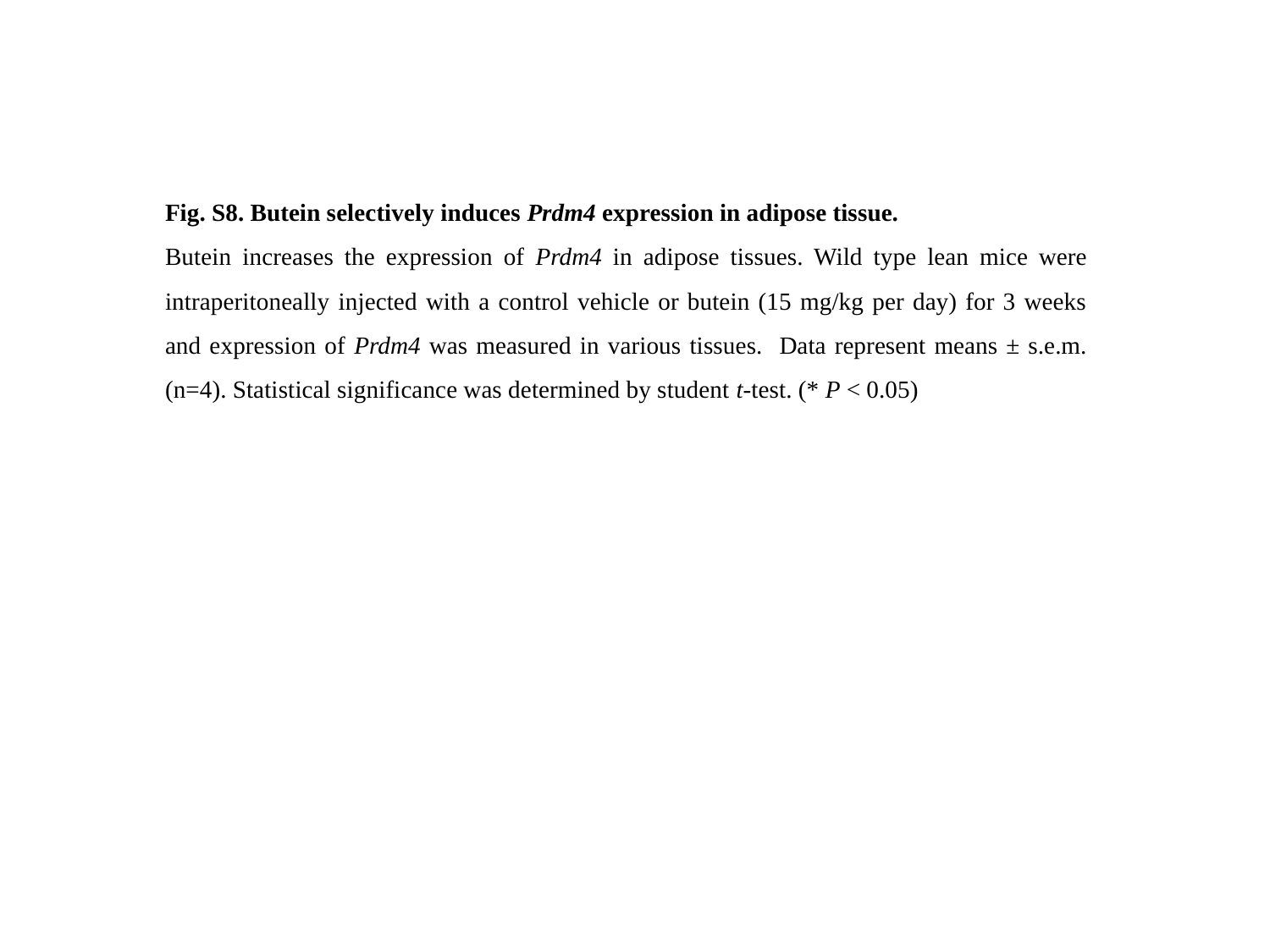

Fig. S8. Butein selectively induces Prdm4 expression in adipose tissue.
Butein increases the expression of Prdm4 in adipose tissues. Wild type lean mice were intraperitoneally injected with a control vehicle or butein (15 mg/kg per day) for 3 weeks and expression of Prdm4 was measured in various tissues. Data represent means ± s.e.m. (n=4). Statistical significance was determined by student t-test. (* P < 0.05)

## Slide 16
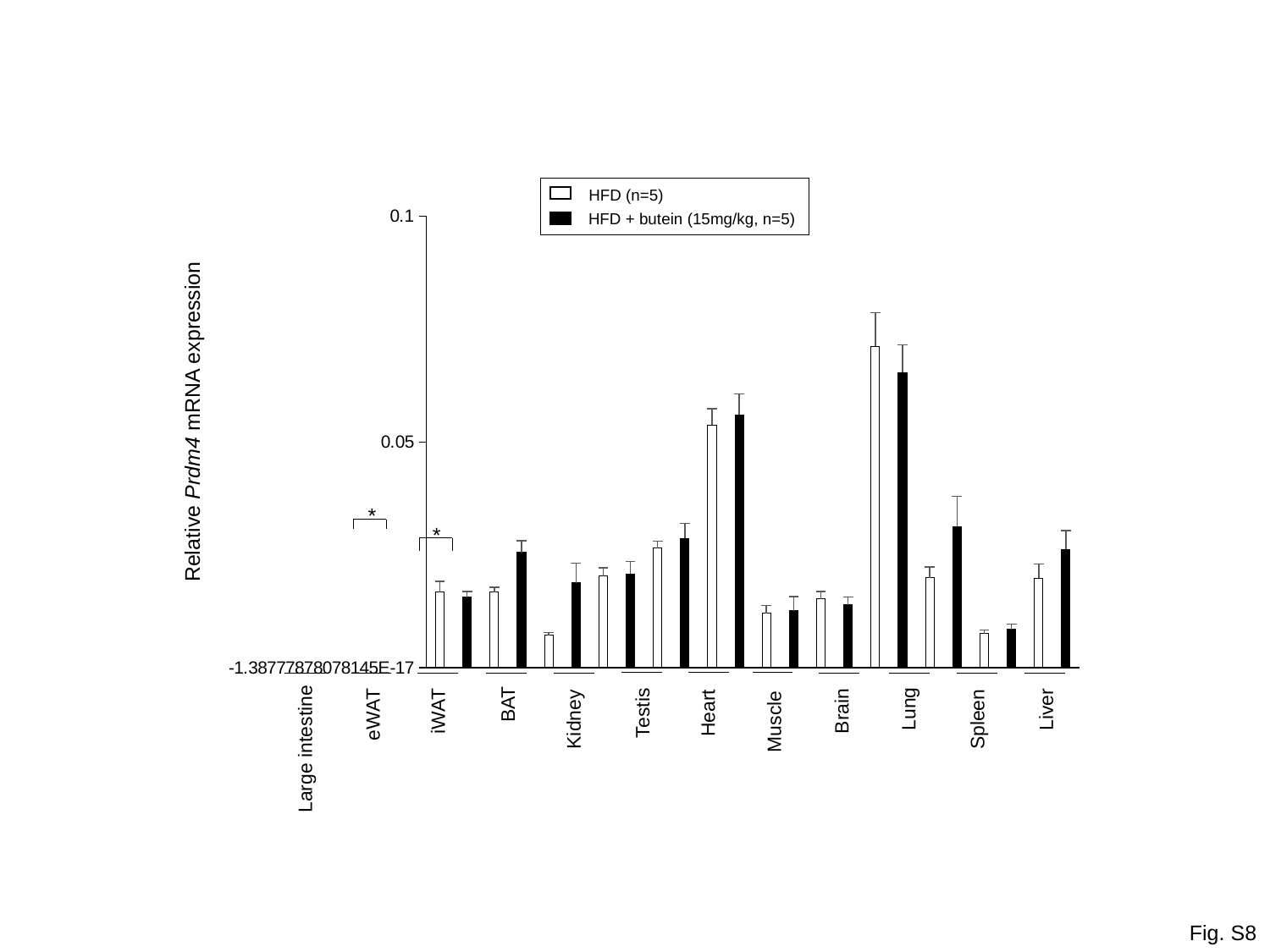

HFD (n=5)
HFD + butein (15mg/kg, n=5)
### Chart
| Category | |
|---|---|
| LI | 0.01675837136389089 |
| LI | 0.015581851213764144 |
| eWAT | 0.016746532652216185 |
| eWAT | 0.025527117587893805 |
| iWAT | 0.0071187899131608294 |
| iWAT | 0.018734218268459578 |
| BAT | 0.020286161841980295 |
| BAT | 0.020718092093245148 |
| Kidney | 0.02647993404328286 |
| Kidney | 0.02865754042663811 |
| Testis | 0.0537054050624874 |
| Testis | 0.05599130414417033 |
| Heart | 0.012012621466559997 |
| Heart | 0.01259736092865632 |
| Muscle | 0.015254020533274263 |
| Muscle | 0.013862844907589317 |
| Brain | 0.07115044922068307 |
| Brain | 0.06535333855285673 |
| Lung | 0.019880413826260643 |
| Lung | 0.03126570351579219 |
| Spleen | 0.0075396789795047275 |
| Spleen | 0.008489650114624698 |
| Liver | 0.01978083697375258 |
| Liver | 0.026135348246585803 |Relative Prdm4 mRNA expression
*
*
BAT
Lung
Liver
iWAT
Brain
Testis
Heart
eWAT
Kidney
Spleen
Muscle
Large intestine
Fig. S8

## Slide 17
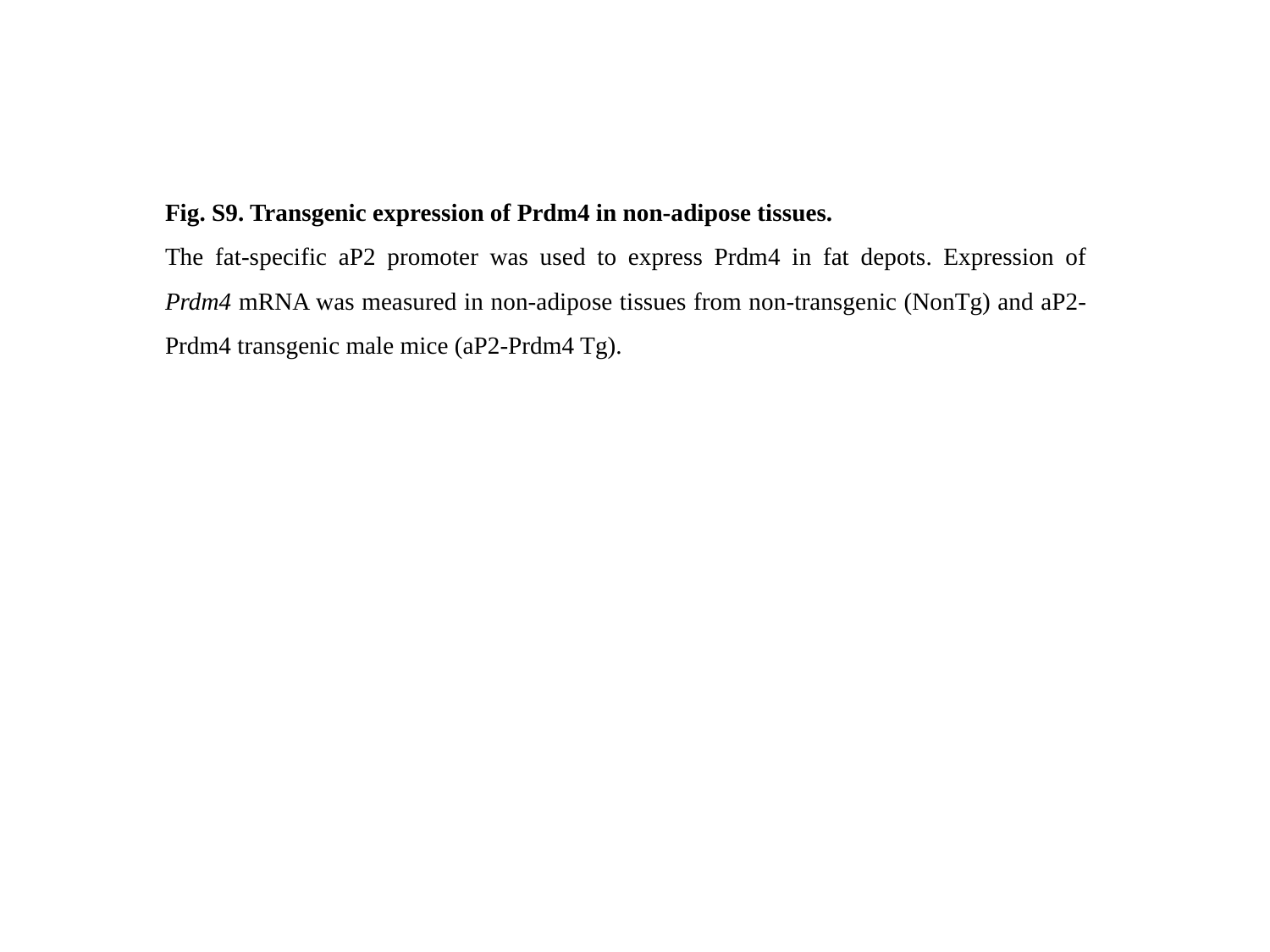

Fig. S9. Transgenic expression of Prdm4 in non-adipose tissues.
The fat-specific aP2 promoter was used to express Prdm4 in fat depots. Expression of Prdm4 mRNA was measured in non-adipose tissues from non-transgenic (NonTg) and aP2-Prdm4 transgenic male mice (aP2-Prdm4 Tg).

## Slide 18
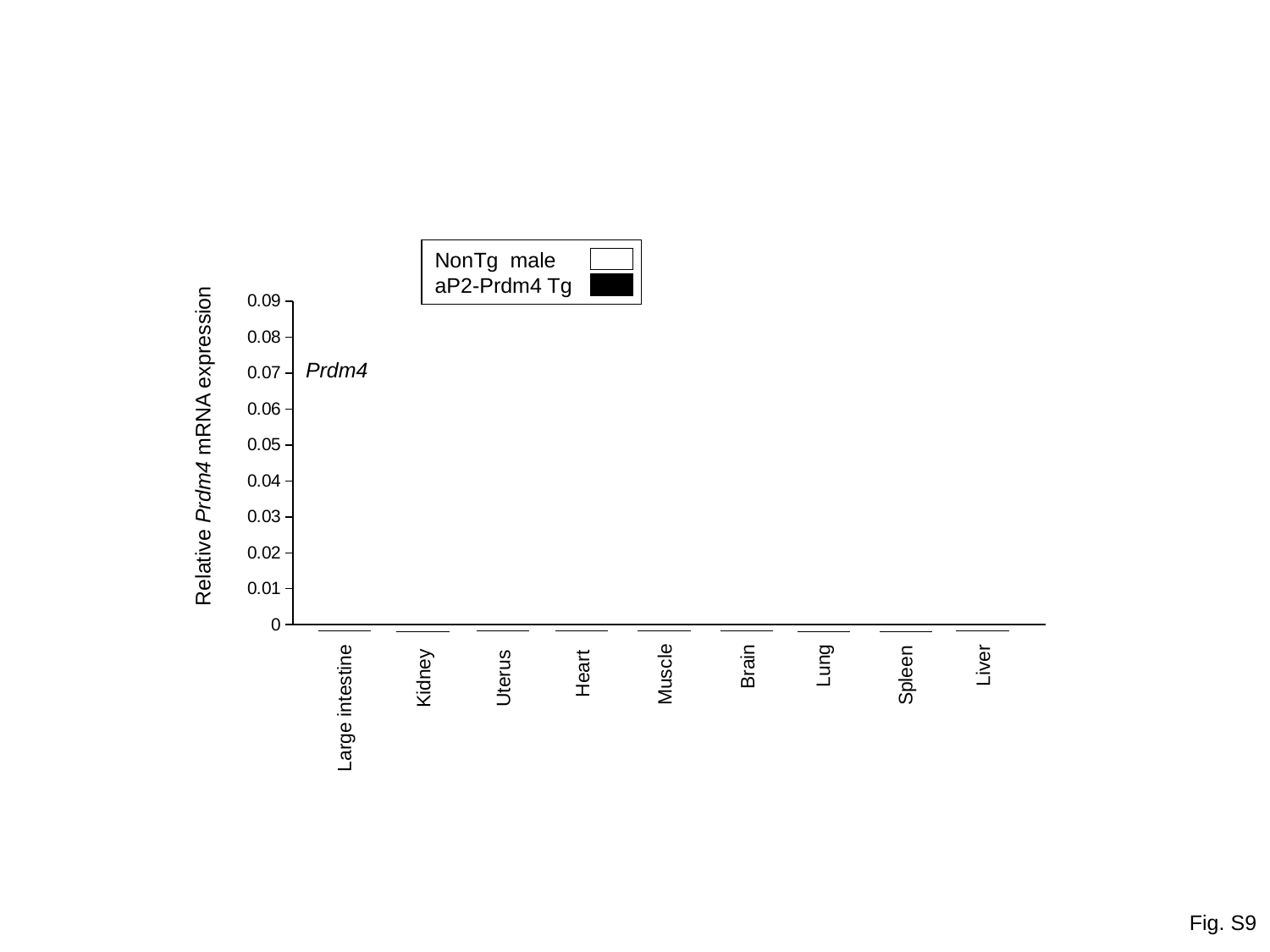

NonTg male
aP2-Prdm4 Tg
Relative Prdm4 mRNA expression
### Chart
| Category | |
|---|---|Prdm4
Lung
Liver
Brain
Heart
Muscle
Spleen
Kidney
Uterus
Large intestine
Fig. S9

## Slide 19
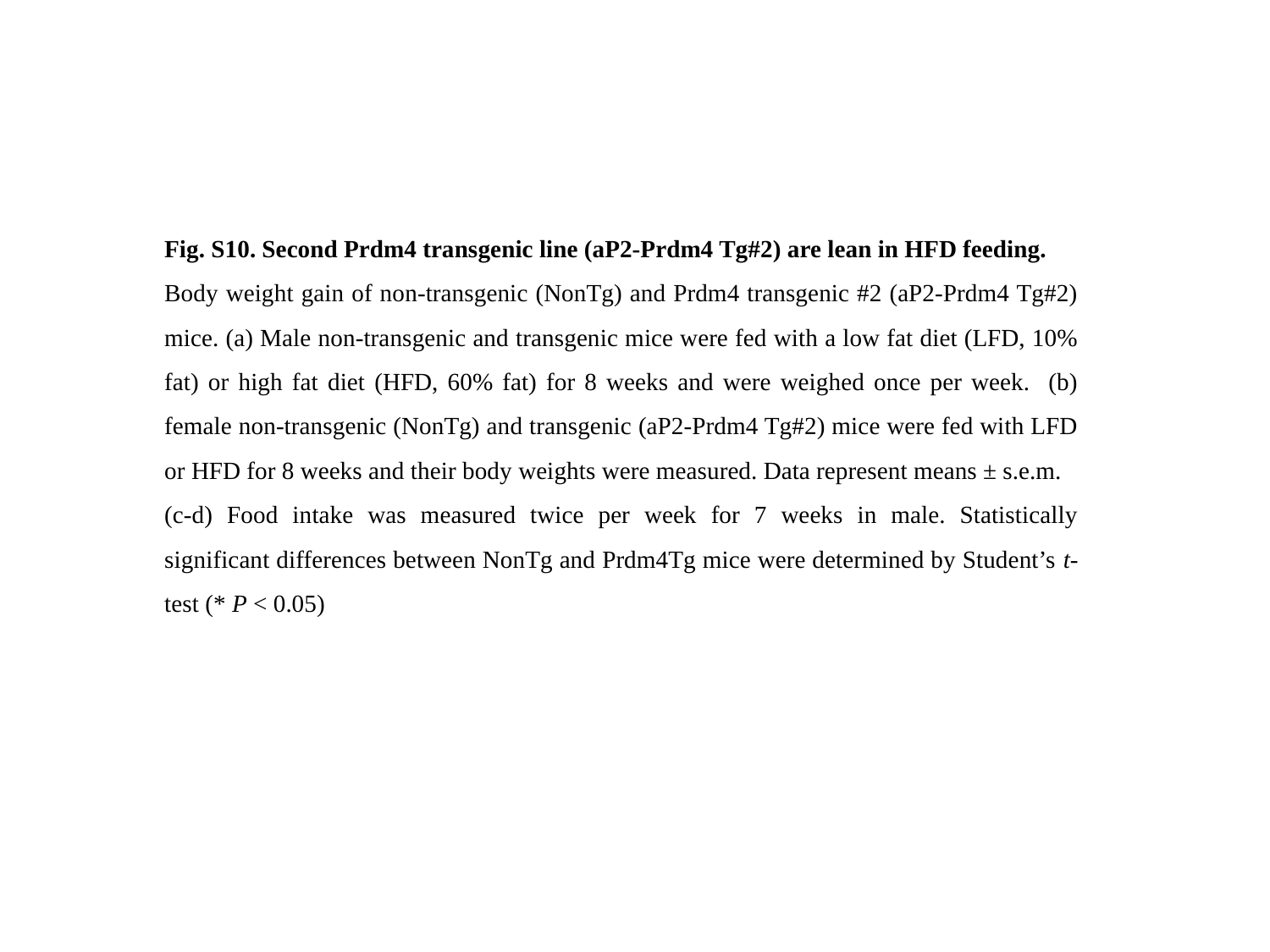

Fig. S10. Second Prdm4 transgenic line (aP2-Prdm4 Tg#2) are lean in HFD feeding.
Body weight gain of non-transgenic (NonTg) and Prdm4 transgenic #2 (aP2-Prdm4 Tg#2) mice. (a) Male non-transgenic and transgenic mice were fed with a low fat diet (LFD, 10% fat) or high fat diet (HFD, 60% fat) for 8 weeks and were weighed once per week. (b) female non-transgenic (NonTg) and transgenic (aP2-Prdm4 Tg#2) mice were fed with LFD or HFD for 8 weeks and their body weights were measured. Data represent means ± s.e.m.
(c-d) Food intake was measured twice per week for 7 weeks in male. Statistically significant differences between NonTg and Prdm4Tg mice were determined by Student’s t-test (* P < 0.05)

## Slide 20
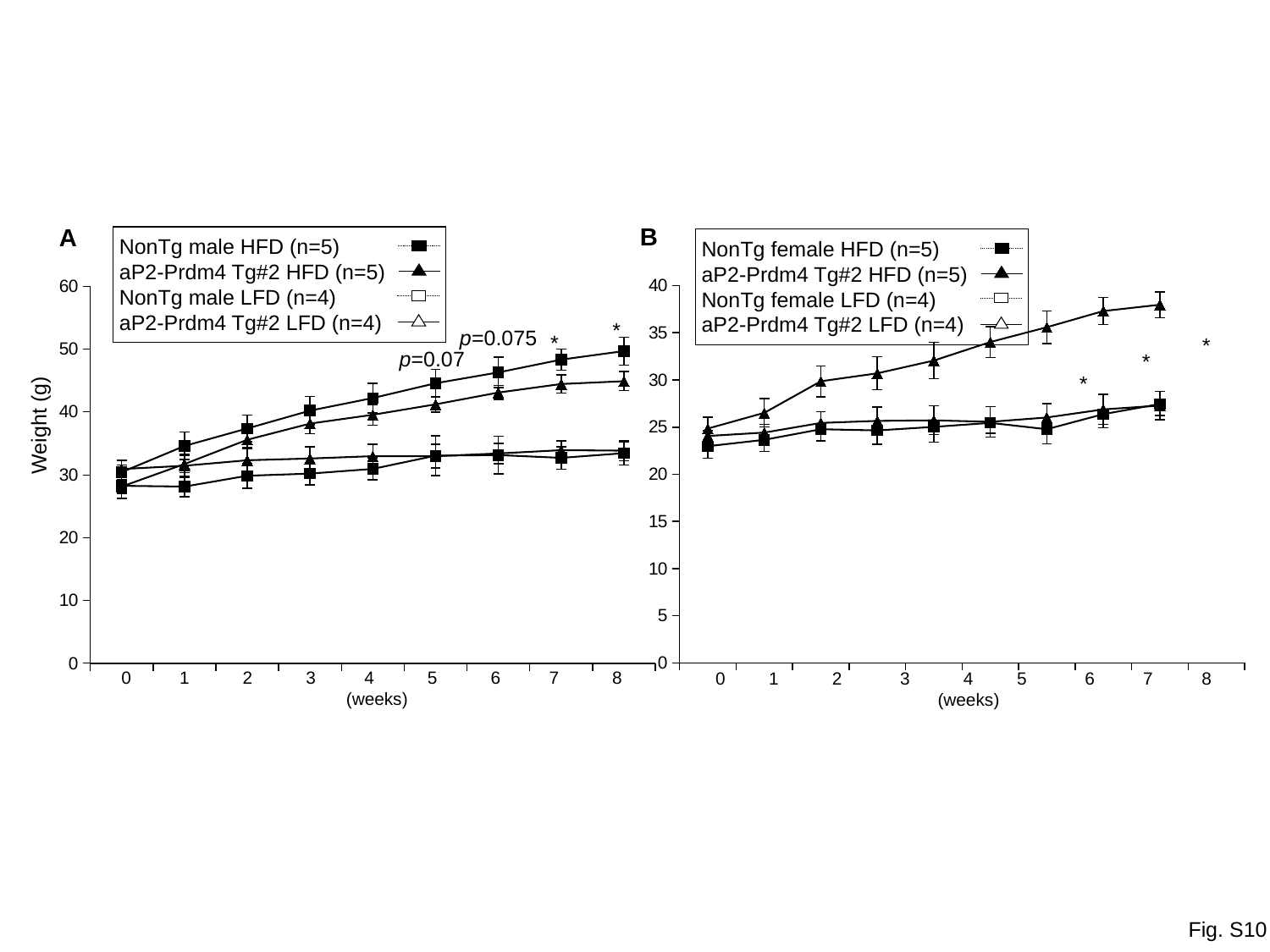

B
A
NonTg male HFD (n=5)
aP2-Prdm4 Tg#2 HFD (n=5)
NonTg male LFD (n=4)
aP2-Prdm4 Tg#2 LFD (n=4)
NonTg female HFD (n=5)
aP2-Prdm4 Tg#2 HFD (n=5)
NonTg female LFD (n=4)
aP2-Prdm4 Tg#2 LFD (n=4)
### Chart
| Category | | | | |
|---|---|---|---|---|
### Chart
| Category | | | | |
|---|---|---|---|---|*
p=0.075
*
*
p=0.07
*
*
Weight (g)
0 1 2 3 4 5 6 7 8
(weeks)
0 1 2 3 4 5 6 7 8
(weeks)
Fig. S10

## Slide 21
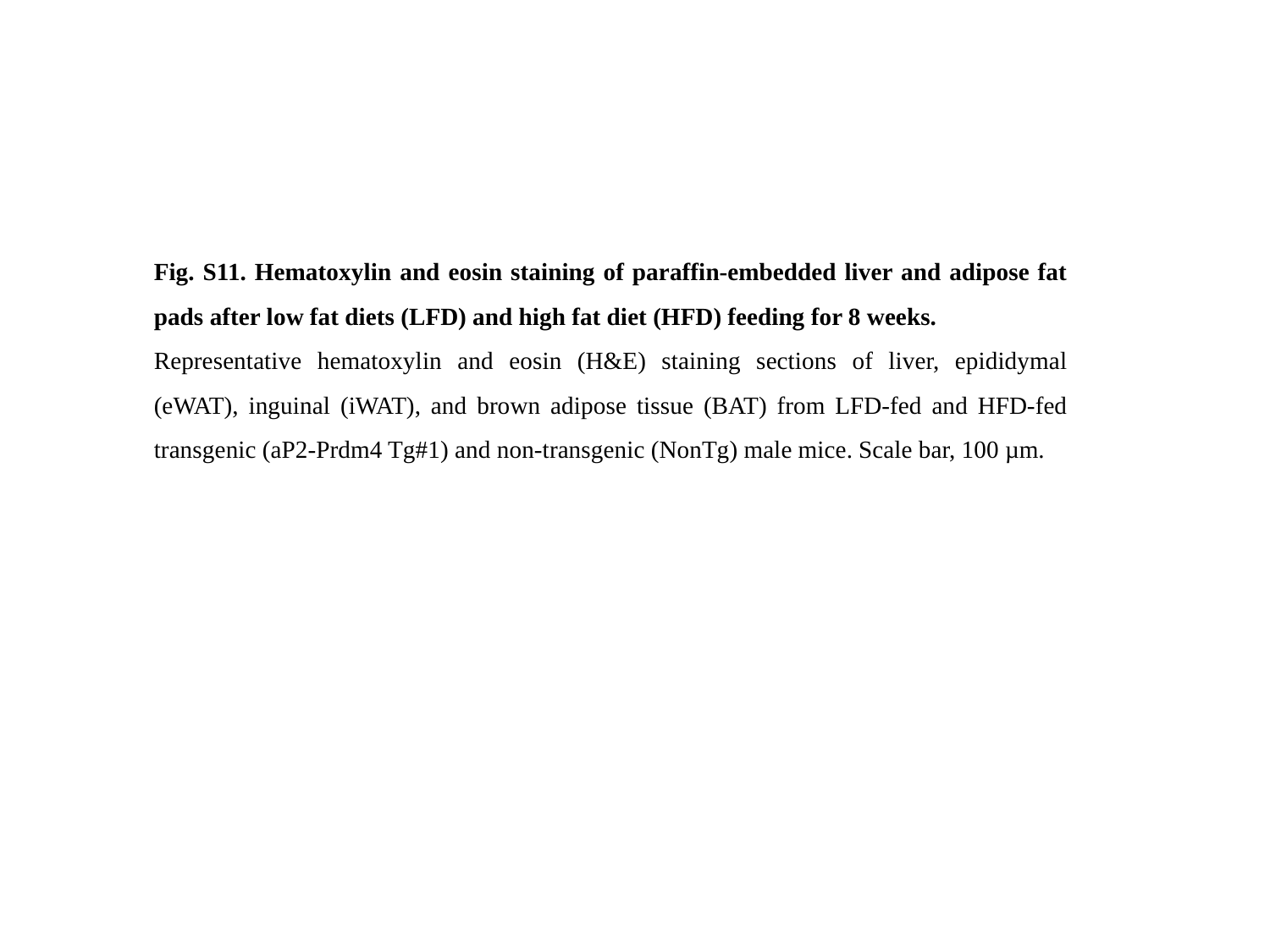

Fig. S11. Hematoxylin and eosin staining of paraffin-embedded liver and adipose fat pads after low fat diets (LFD) and high fat diet (HFD) feeding for 8 weeks.
Representative hematoxylin and eosin (H&E) staining sections of liver, epididymal (eWAT), inguinal (iWAT), and brown adipose tissue (BAT) from LFD-fed and HFD-fed transgenic (aP2-Prdm4 Tg#1) and non-transgenic (NonTg) male mice. Scale bar, 100 µm.

## Slide 22
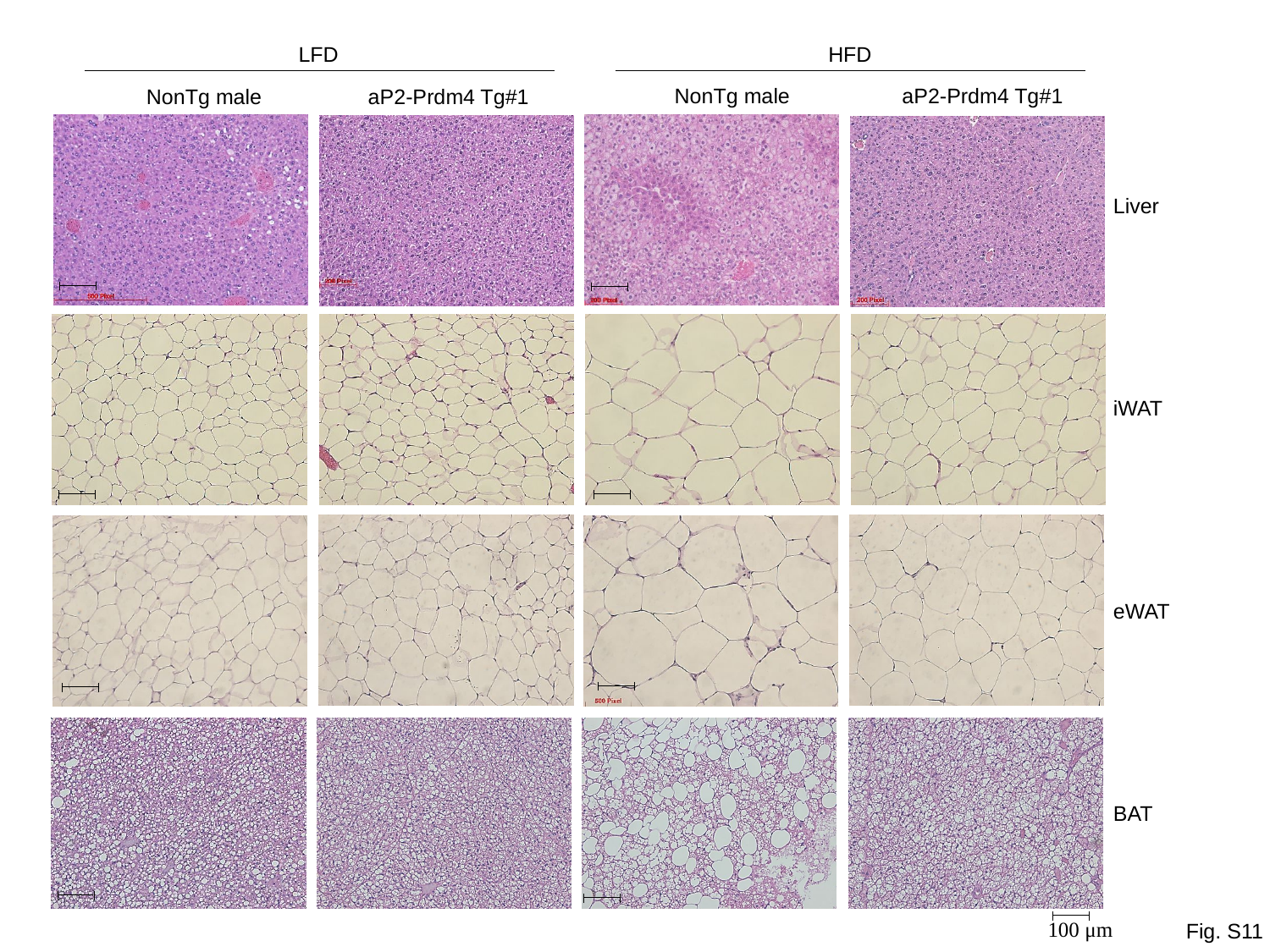

HFD
LFD
NonTg male aP2-Prdm4 Tg#1
NonTg male aP2-Prdm4 Tg#1
Liver
iWAT
eWAT
BAT
100 μm
Fig. S11

## Slide 23
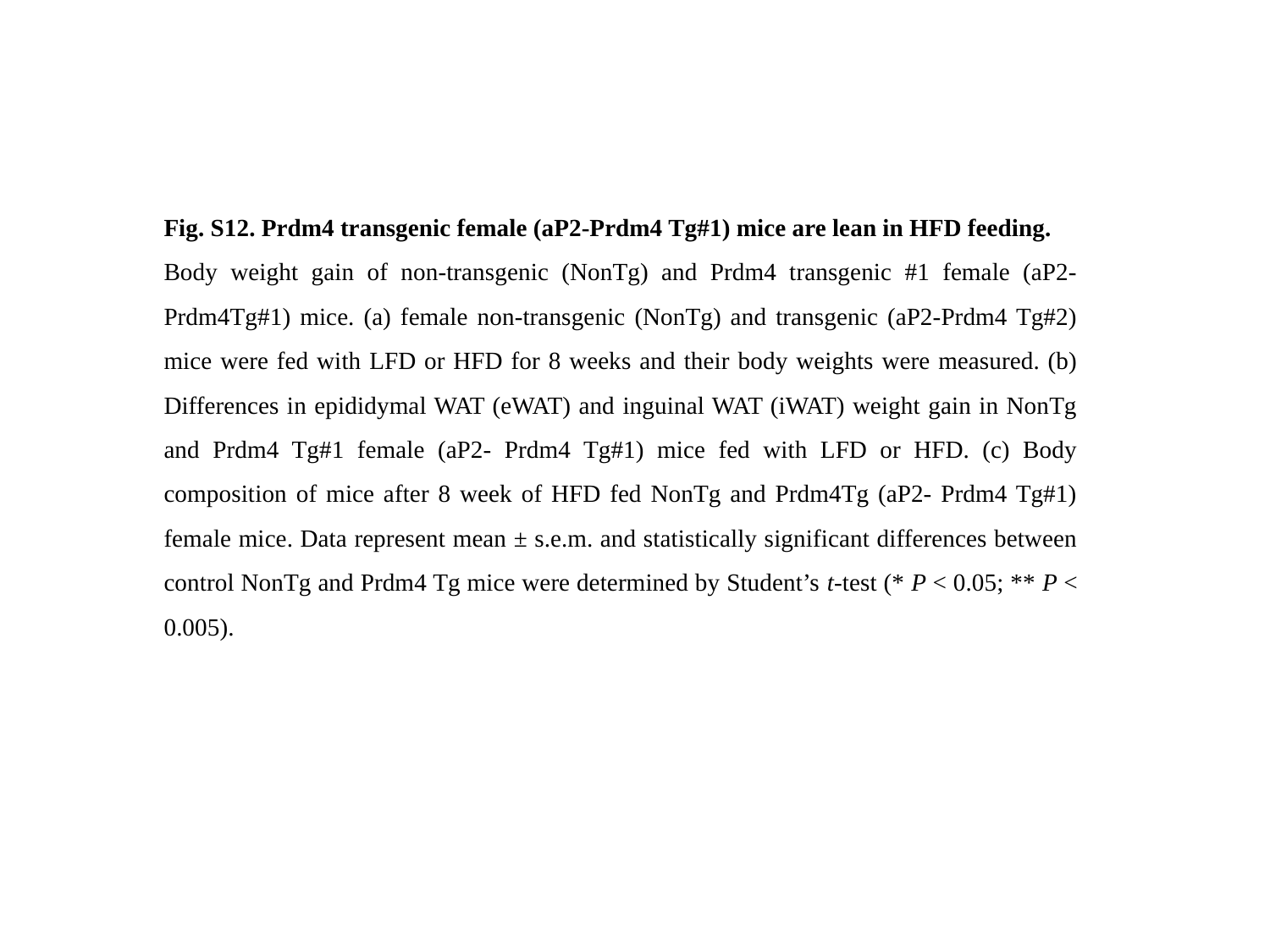

Fig. S12. Prdm4 transgenic female (aP2-Prdm4 Tg#1) mice are lean in HFD feeding.
Body weight gain of non-transgenic (NonTg) and Prdm4 transgenic #1 female (aP2-Prdm4Tg#1) mice. (a) female non-transgenic (NonTg) and transgenic (aP2-Prdm4 Tg#2) mice were fed with LFD or HFD for 8 weeks and their body weights were measured. (b) Differences in epididymal WAT (eWAT) and inguinal WAT (iWAT) weight gain in NonTg and Prdm4 Tg#1 female (aP2- Prdm4 Tg#1) mice fed with LFD or HFD. (c) Body composition of mice after 8 week of HFD fed NonTg and Prdm4Tg (aP2- Prdm4 Tg#1) female mice. Data represent mean ± s.e.m. and statistically significant differences between control NonTg and Prdm4 Tg mice were determined by Student’s t-test (* P < 0.05; ** P < 0.005).

## Slide 24
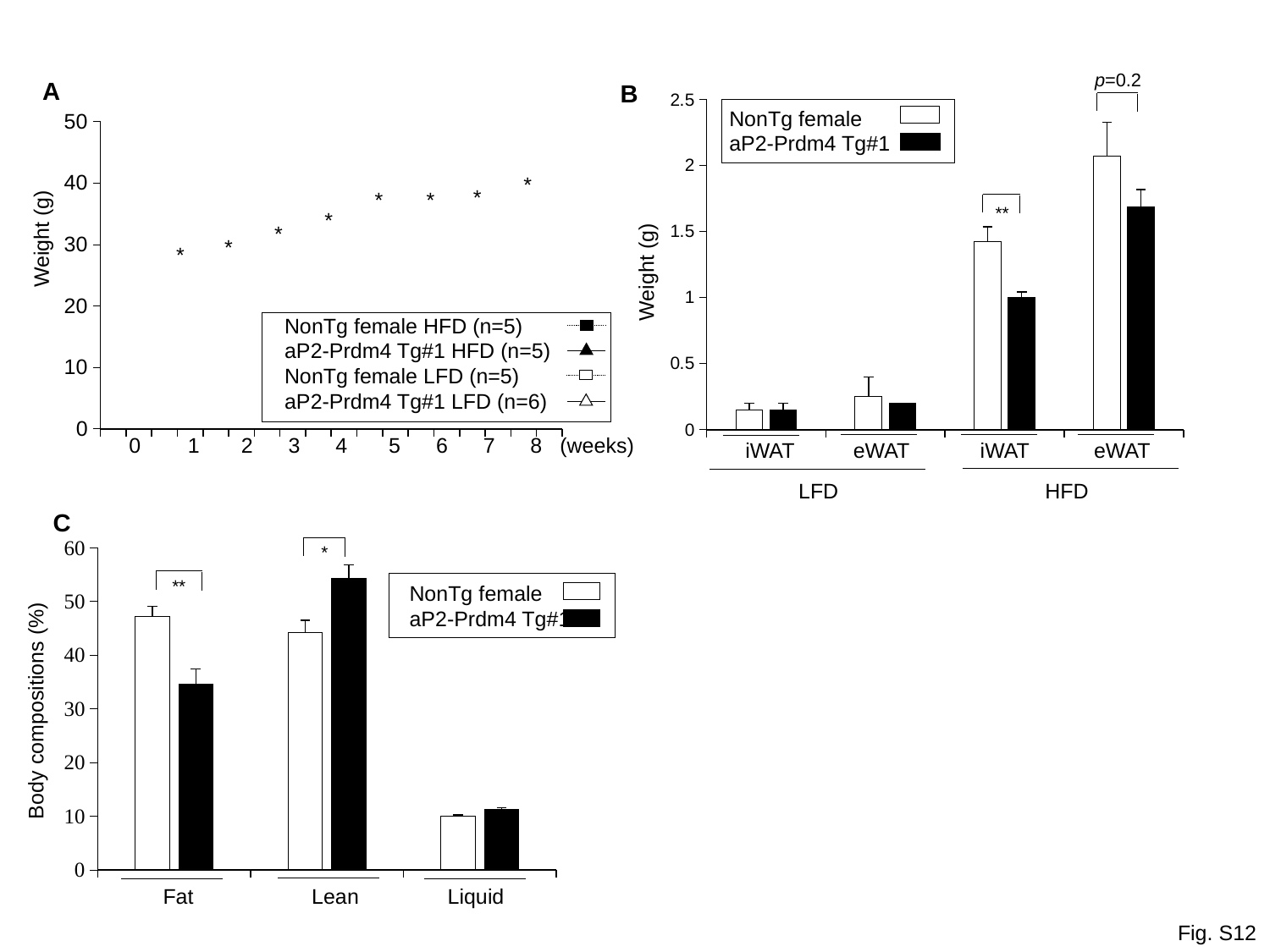

p=0.2
A
B
### Chart
| Category | | |
|---|---|---|NonTg female
aP2-Prdm4 Tg#1
### Chart
| Category | | | | |
|---|---|---|---|---|*
*
*
*
**
*
*
Weight (g)
*
*
Weight (g)
NonTg female HFD (n=5)
aP2-Prdm4 Tg#1 HFD (n=5)
NonTg female LFD (n=5)
aP2-Prdm4 Tg#1 LFD (n=6)
0 1 2 3 4 5 6 7 8 (weeks)
iWAT eWAT iWAT eWAT
LFD HFD
C
### Chart
| Category | | |
|---|---|---|*
**
NonTg female
aP2-Prdm4 Tg#1
Body compositions (%)
 Fat Lean Liquid
Fig. S12

## Slide 25
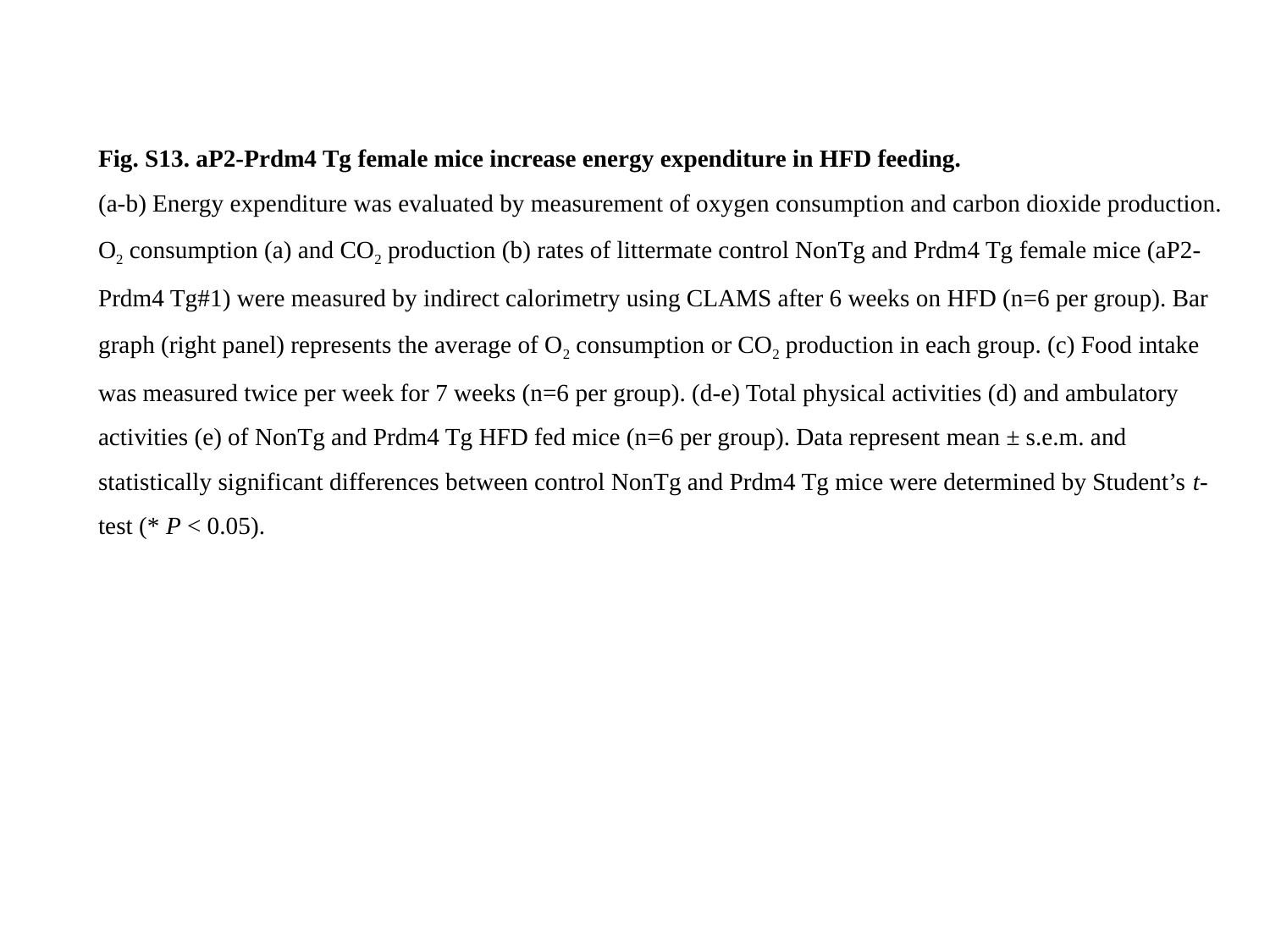

Fig. S13. aP2-Prdm4 Tg female mice increase energy expenditure in HFD feeding.
(a-b) Energy expenditure was evaluated by measurement of oxygen consumption and carbon dioxide production. O2 consumption (a) and CO2 production (b) rates of littermate control NonTg and Prdm4 Tg female mice (aP2-Prdm4 Tg#1) were measured by indirect calorimetry using CLAMS after 6 weeks on HFD (n=6 per group). Bar graph (right panel) represents the average of O2 consumption or CO2 production in each group. (c) Food intake was measured twice per week for 7 weeks (n=6 per group). (d-e) Total physical activities (d) and ambulatory activities (e) of NonTg and Prdm4 Tg HFD fed mice (n=6 per group). Data represent mean ± s.e.m. and statistically significant differences between control NonTg and Prdm4 Tg mice were determined by Student’s t-test (* P < 0.05).

## Slide 26
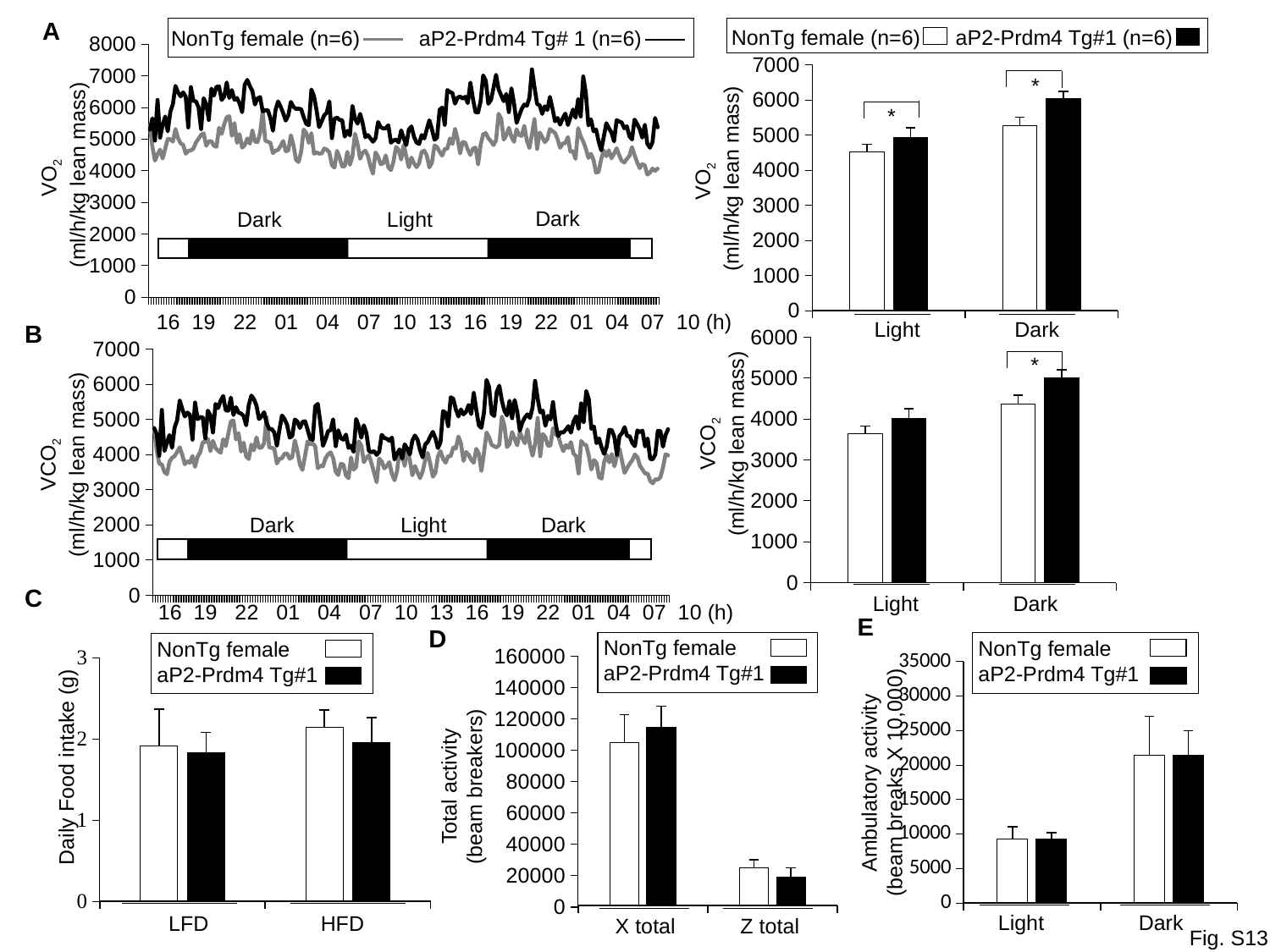

A
NonTg female (n=6) aP2-Prdm4 Tg#1 (n=6)
NonTg female (n=6) aP2-Prdm4 Tg# 1 (n=6)
### Chart
| Category | | |
|---|---|---|
### Chart
| Category | | |
|---|---|---|*
*
VO2
(ml/h/kg lean mass)
VO2
(ml/h/kg lean mass)
Dark
Light
Dark
16 19 22 01 04 07 10 13 16 19 22 01 04 07 10 (h)
Light Dark
B
### Chart
| Category | | |
|---|---|---|
### Chart
| Category | | |
|---|---|---|*
VCO2
(ml/h/kg lean mass)
VCO2
(ml/h/kg lean mass)
Dark Light Dark
C
Light Dark
16 19 22 01 04 07 10 13 16 19 22 01 04 07 10 (h)
E
D
NonTg female
aP2-Prdm4 Tg#1
NonTg female
aP2-Prdm4 Tg#1
NonTg female
aP2-Prdm4 Tg#1
### Chart
| Category | | |
|---|---|---|
### Chart
| Category | | |
|---|---|---|
### Chart
| Category | | |
|---|---|---|Daily Food intake (g)
Ambulatory activity
(beam breaks X 10,000)
Total activity
(beam breakers)
Light Dark
LFD HFD
X total Z total
Fig. S13

## Slide 27
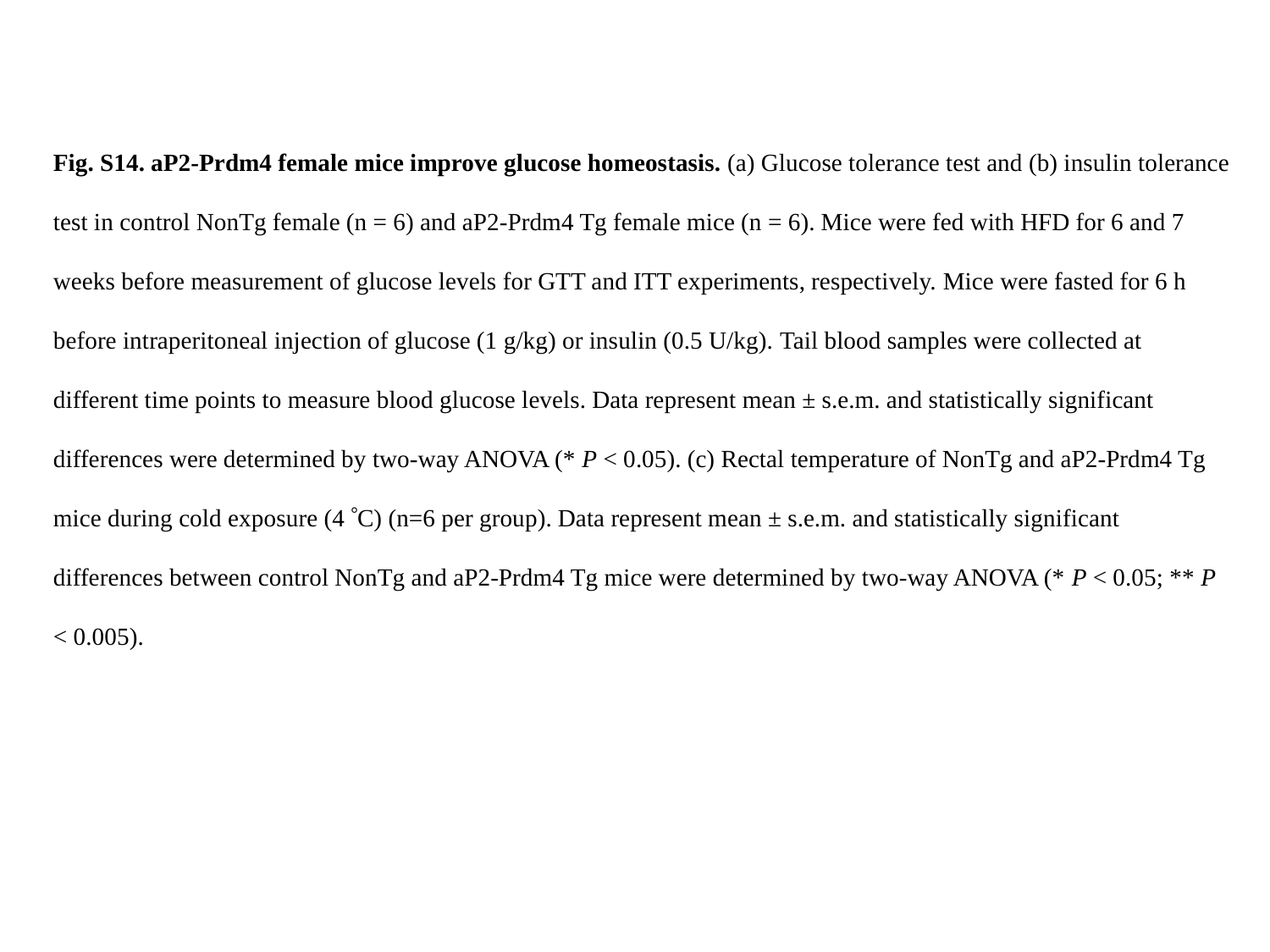

Fig. S14. aP2-Prdm4 female mice improve glucose homeostasis. (a) Glucose tolerance test and (b) insulin tolerance test in control NonTg female (n = 6) and aP2-Prdm4 Tg female mice (n = 6). Mice were fed with HFD for 6 and 7 weeks before measurement of glucose levels for GTT and ITT experiments, respectively. Mice were fasted for 6 h before intraperitoneal injection of glucose (1 g/kg) or insulin (0.5 U/kg). Tail blood samples were collected at different time points to measure blood glucose levels. Data represent mean ± s.e.m. and statistically significant differences were determined by two-way ANOVA (* P < 0.05). (c) Rectal temperature of NonTg and aP2-Prdm4 Tg mice during cold exposure (4 °C) (n=6 per group). Data represent mean ± s.e.m. and statistically significant differences between control NonTg and aP2-Prdm4 Tg mice were determined by two-way ANOVA (* P < 0.05; ** P < 0.005).

## Slide 28
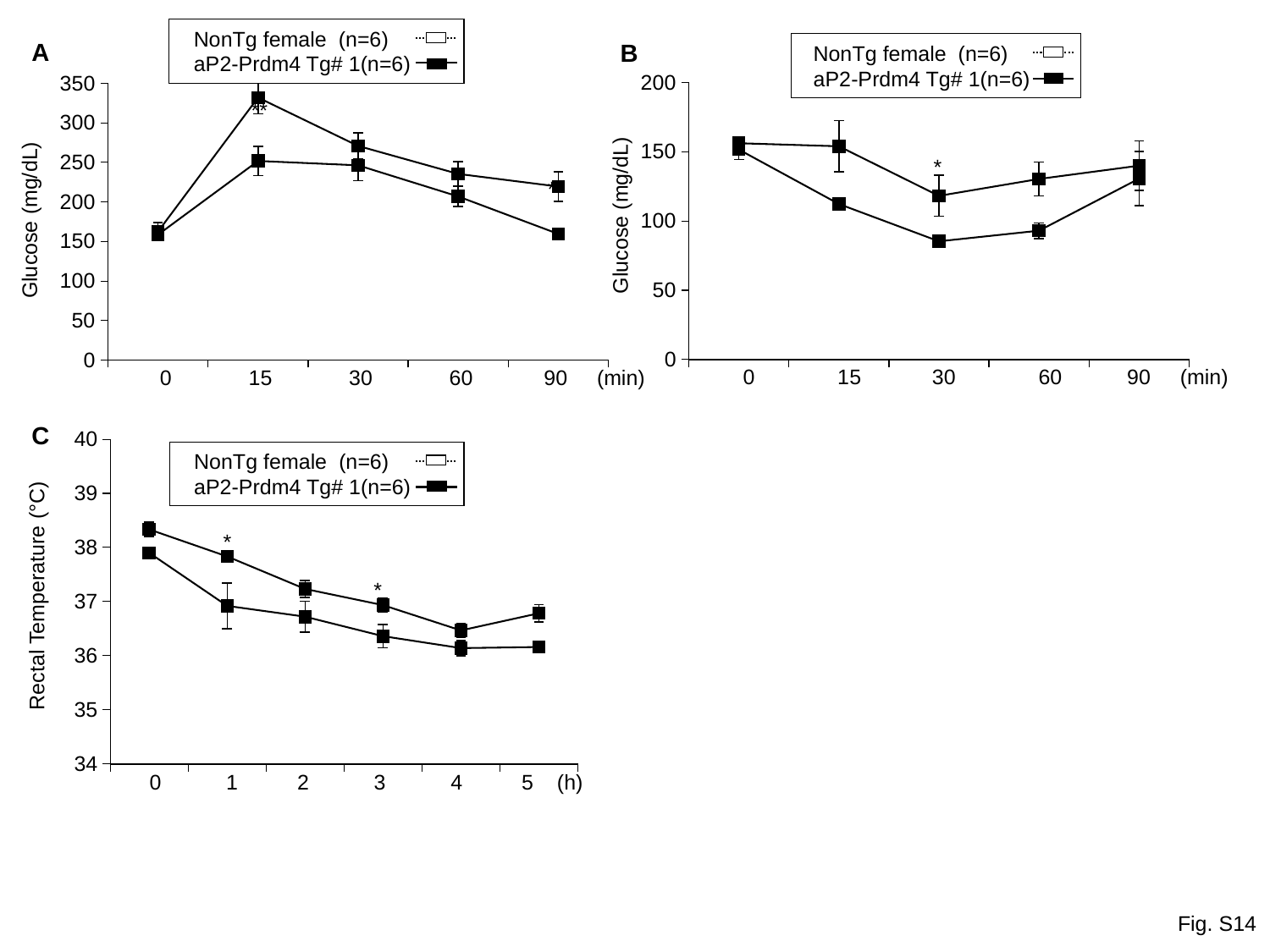

NonTg female (n=6)
aP2-Prdm4 Tg# 1(n=6)
A
B
NonTg female (n=6)
aP2-Prdm4 Tg# 1(n=6)
### Chart
| Category | | |
|---|---|---|
### Chart
| Category | | |
|---|---|---|**
*
*
Glucose (mg/dL)
Glucose (mg/dL)
0 15 30 60 90 (min)
0 15 30 60 90 (min)
C
### Chart
| Category | | |
|---|---|---|
NonTg female (n=6)
aP2-Prdm4 Tg# 1(n=6)
Rectal Temperature (°C)
*
*
0 1 2 3 4 5 (h)
Fig. S14

## Slide 29
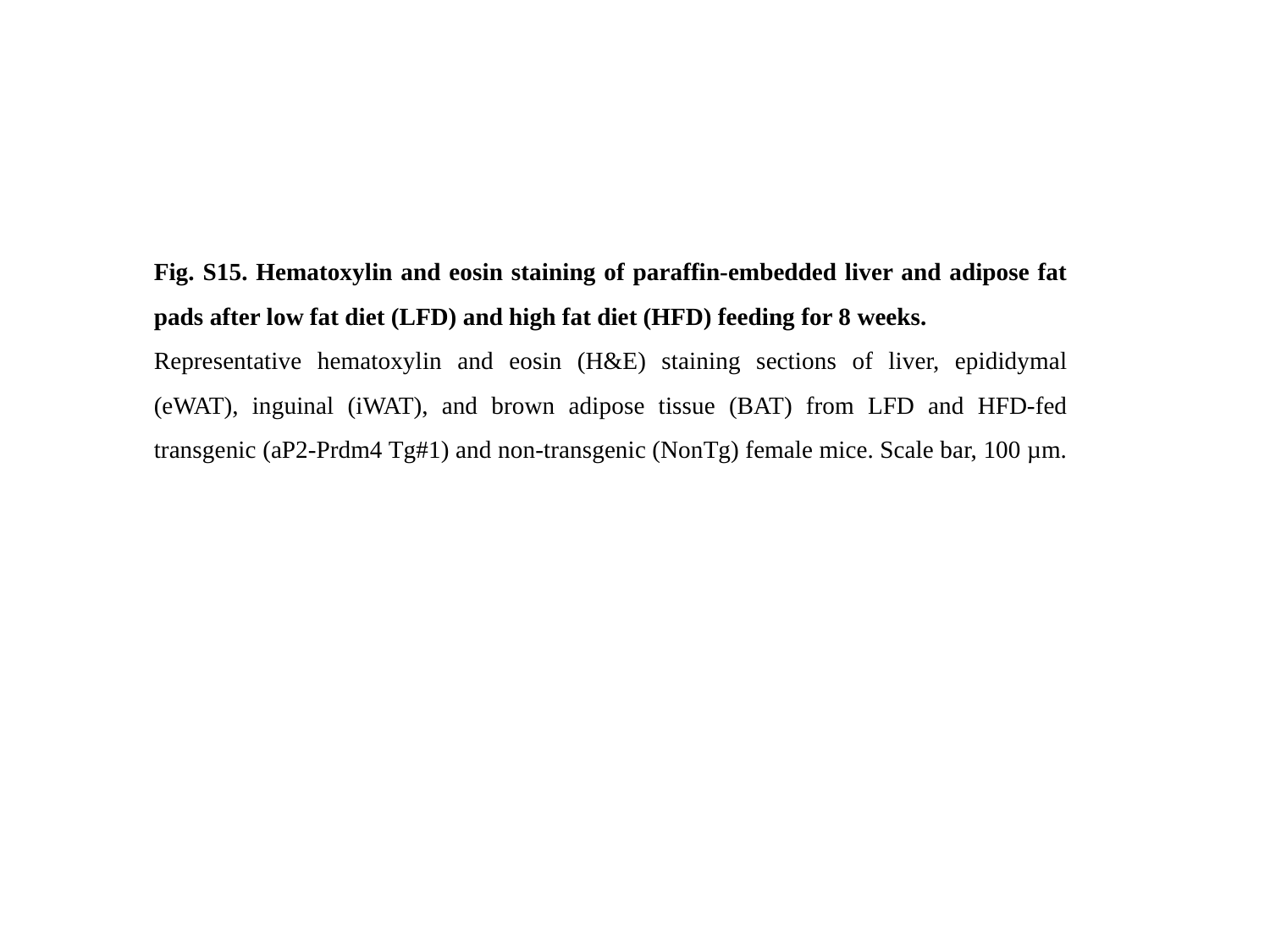

Fig. S15. Hematoxylin and eosin staining of paraffin-embedded liver and adipose fat pads after low fat diet (LFD) and high fat diet (HFD) feeding for 8 weeks.
Representative hematoxylin and eosin (H&E) staining sections of liver, epididymal (eWAT), inguinal (iWAT), and brown adipose tissue (BAT) from LFD and HFD-fed transgenic (aP2-Prdm4 Tg#1) and non-transgenic (NonTg) female mice. Scale bar, 100 µm.

## Slide 30
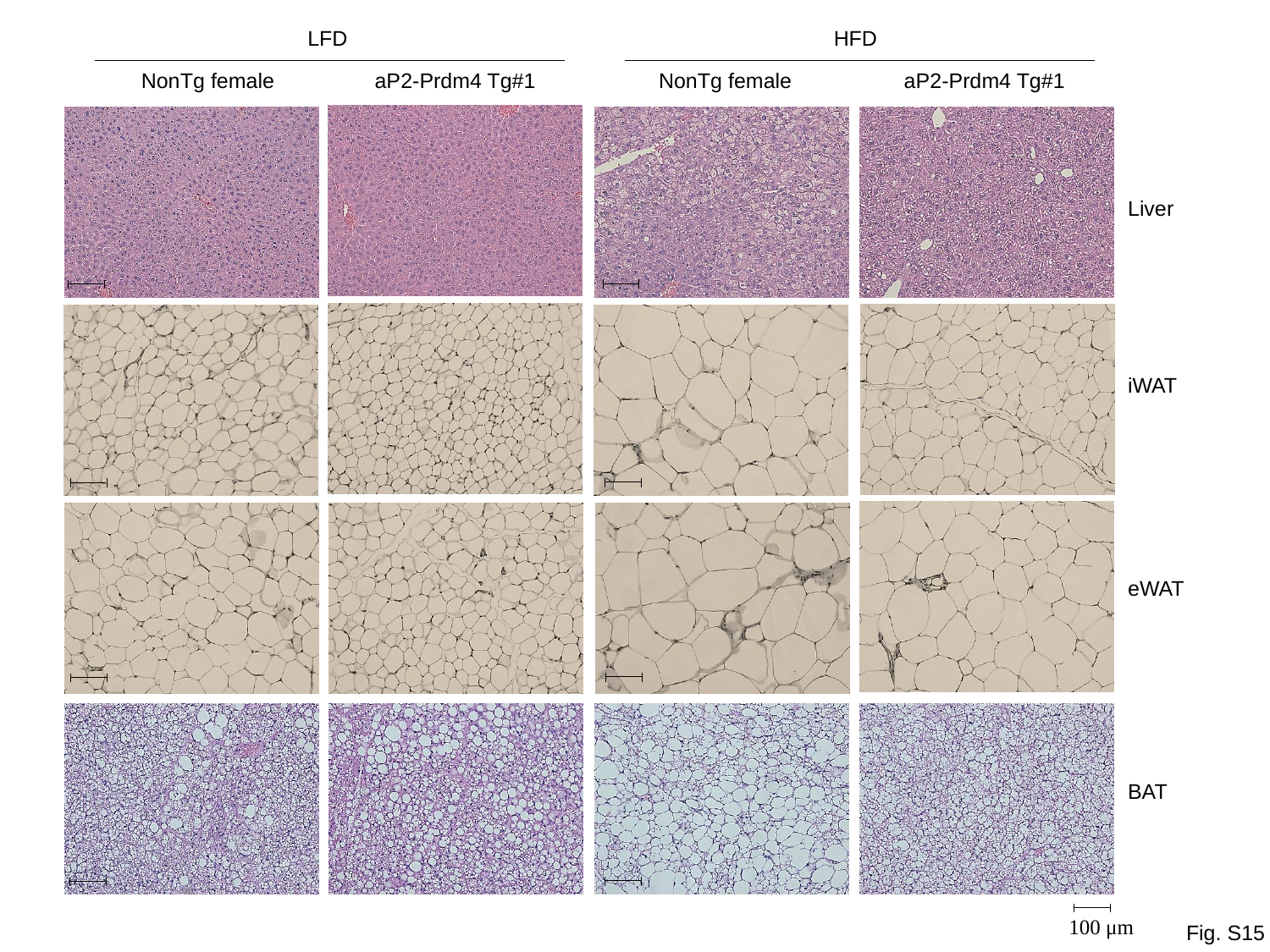

LFD
HFD
NonTg female aP2-Prdm4 Tg#1
NonTg female aP2-Prdm4 Tg#1
Liver
iWAT
eWAT
BAT
100 μm
Fig. S15

## Slide 31
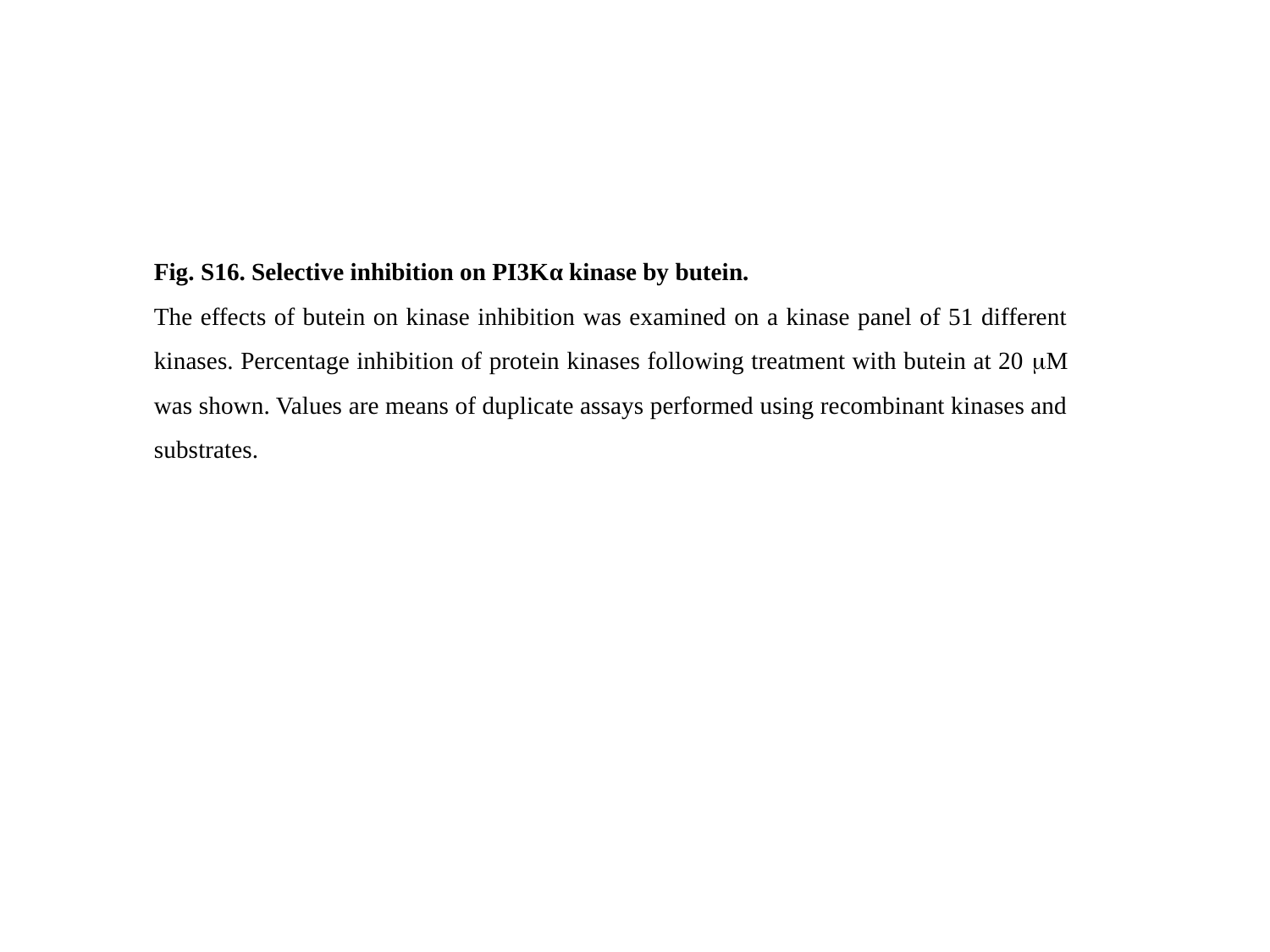

Fig. S16. Selective inhibition on PI3Kα kinase by butein.
The effects of butein on kinase inhibition was examined on a kinase panel of 51 different kinases. Percentage inhibition of protein kinases following treatment with butein at 20 μM was shown. Values are means of duplicate assays performed using recombinant kinases and substrates.

## Slide 32
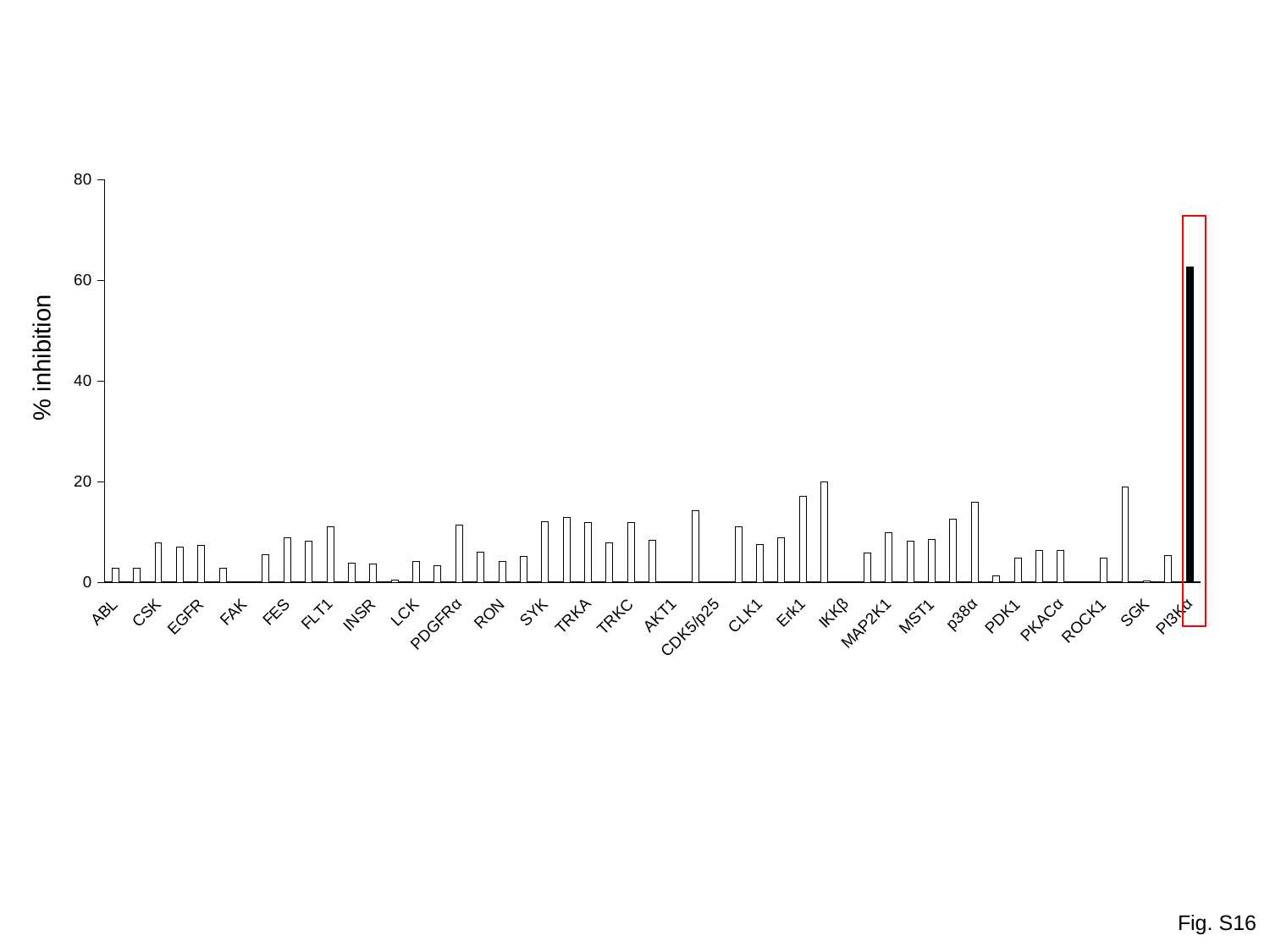

### Chart
| Category | |
|---|---|
| ABL | 2.867038676159339 |
| ALK | 2.7923410075222144 |
| CSK | 7.831181252989017 |
| DDR1 | 6.950489662676818 |
| EGFR | 7.332690114902196 |
| EPHA1 | 2.7648642035722926 |
| FAK | -0.40202929070545235 |
| FER | 5.4734620024125435 |
| FES | 8.924818108326594 |
| FGFR1 | 8.203546646106386 |
| FLT1 | 11.099777097736974 |
| IGF1R | 3.727399165507661 |
| INSR | 3.662283396829291 |
| IRR | 0.4280540006585398 |
| LCK | 4.198895027624316 |
| MET | 3.3474801061007975 |
| PDGFRα | 11.362363483618044 |
| PYK2 | 5.965133375341325 |
| RON | 4.120603015075385 |
| SRC | 5.2088985349972745 |
| SYK | 11.99786822996468 |
| TIE2 | 12.890407138873384 |
| TRKA | 11.821654373024227 |
| TRKB | 7.871462264150941 |
| TRKC | 11.904761904761907 |
| TYK2 | 8.379558597899205 |
| AKT1 | -3.423731226421811 |
| AMPKα1/β1/γ1 | 14.299326642103916 |
| CDK5/p25 | -1.0889973713856582 |
| CK1α | 11.097972972972958 |
| CLK1 | 7.570325427468294 |
| DYRK1A | 8.910370915204357 |
| Erk1 | 17.079573420836745 |
| GSK3β | 19.96009794141652 |
| IKKβ | -12.131890259249944 |
| JNK1 | 5.8727834716121645 |
| MAP2K1 | 9.819595981419472 |
| MAP3K1 | 8.202531645569621 |
| MST1 | 8.5114036981078 |
| NEK1 | 12.596444146259655 |
| p38α | 15.832669810742939 |
| p70S6K | 1.2074864157778253 |
| PDK1 | 4.7345043408035465 |
| PIM1 | 6.371135064014977 |
| PKACα | 6.402952740110724 |
| PKCα | -2.4768468662502707 |
| ROCK1 | 4.802289833563023 |
| RSK1 | 18.865757358219646 |
| SGK | 0.1939702948348443 |
| SIK | 5.369415807560141 |
| PI3Kα | 62.6 |
% inhibition
Fig. S16

## Slide 33
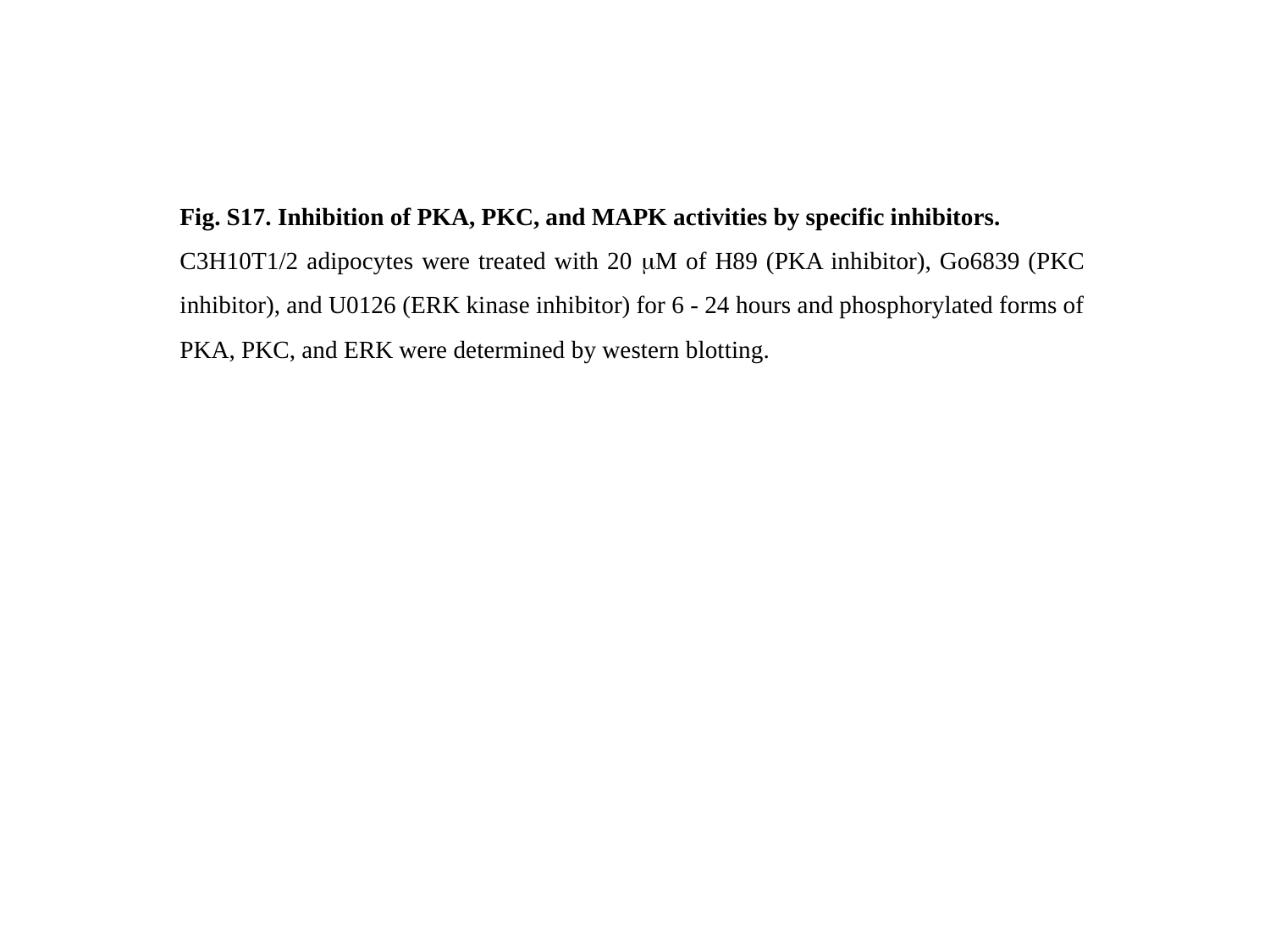

Fig. S17. Inhibition of PKA, PKC, and MAPK activities by specific inhibitors.
C3H10T1/2 adipocytes were treated with 20 μM of H89 (PKA inhibitor), Go6839 (PKC inhibitor), and U0126 (ERK kinase inhibitor) for 6 - 24 hours and phosphorylated forms of PKA, PKC, and ERK were determined by western blotting.

## Slide 34
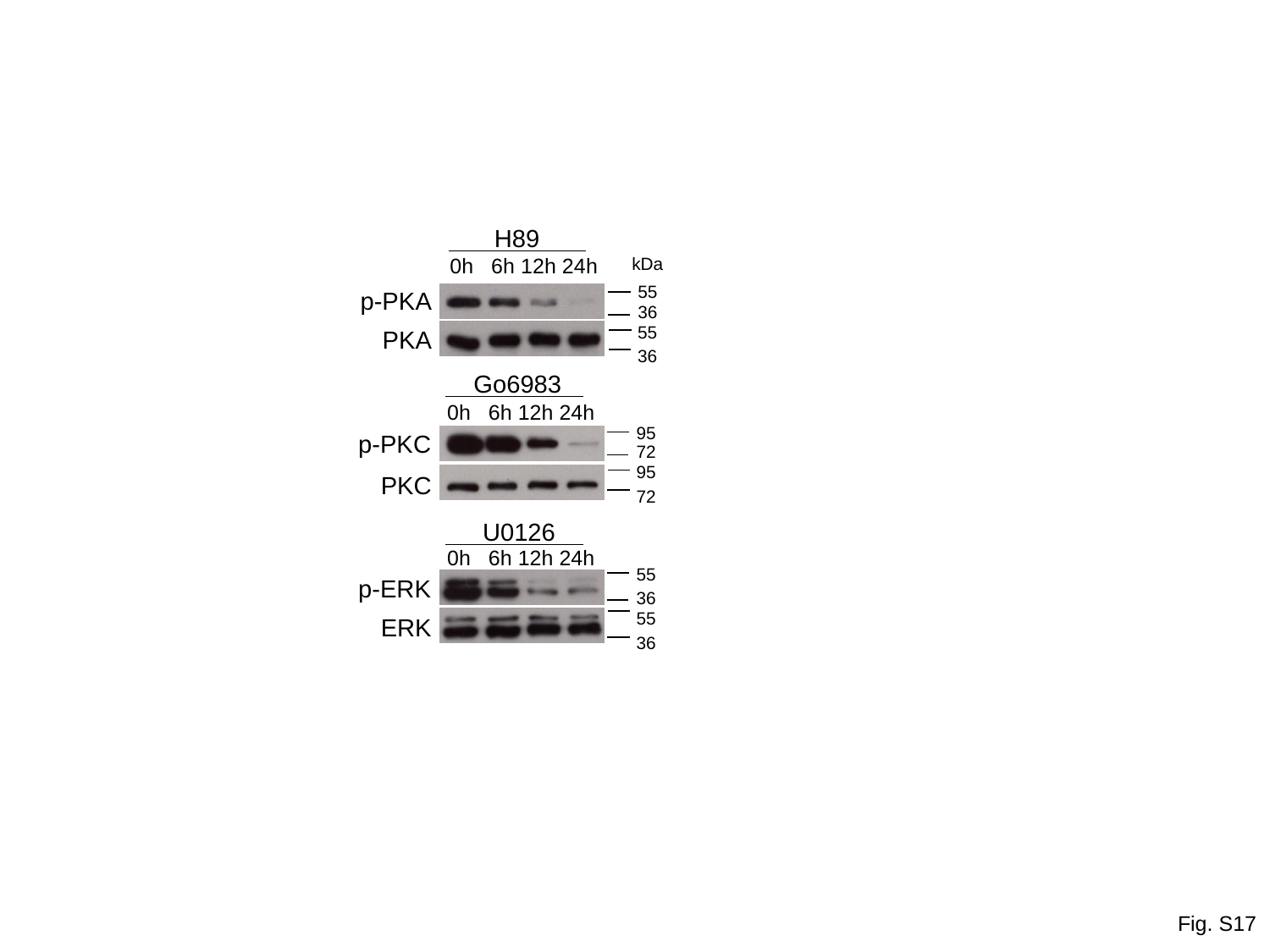

H89
0h 6h 12h 24h
kDa
55
p-PKA
36
55
PKA
36
Go6983
0h 6h 12h 24h
95
p-PKC
72
95
PKC
72
U0126
0h 6h 12h 24h
55
p-ERK
36
55
ERK
36
Fig. S17

## Slide 35
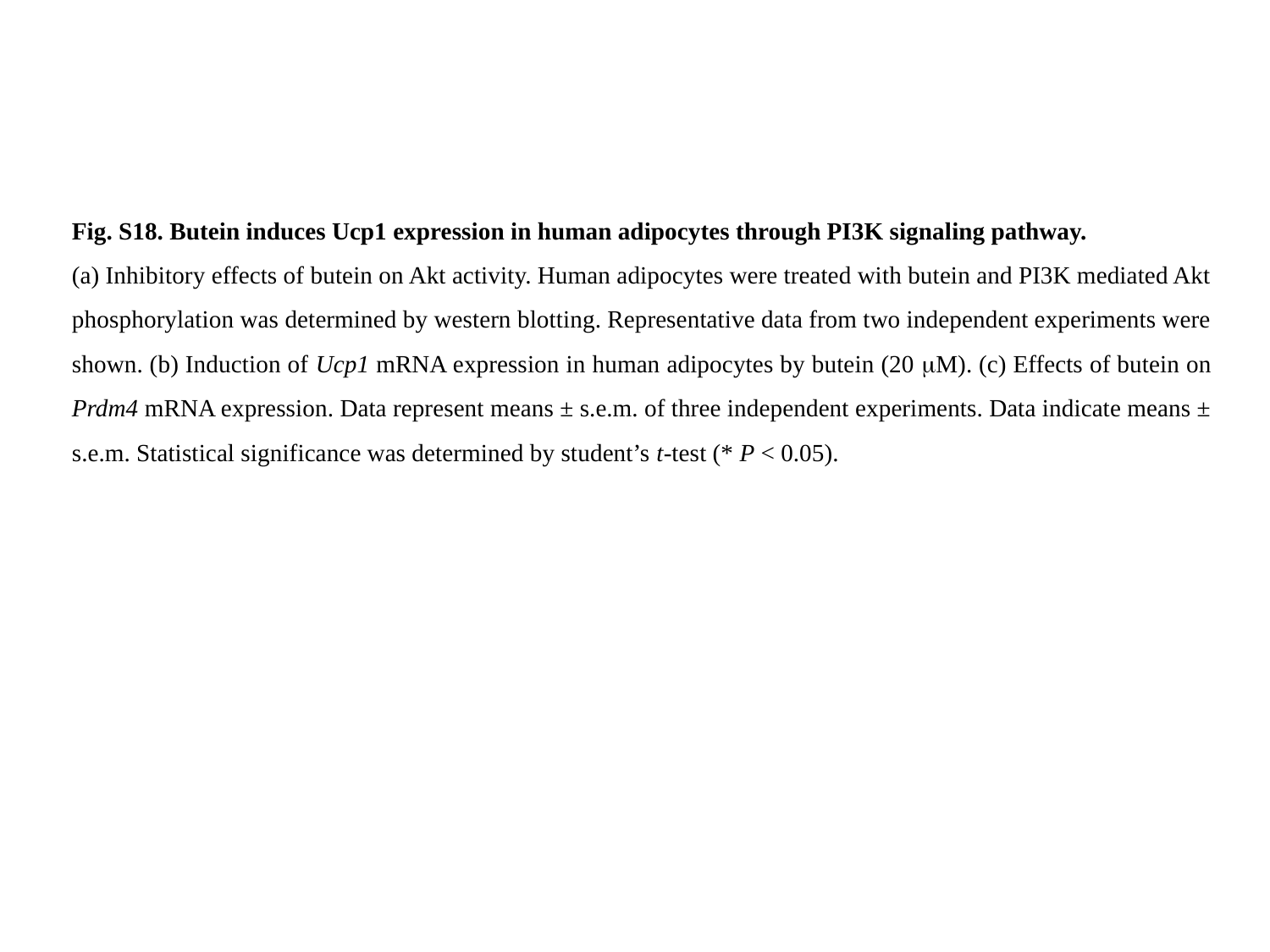

Fig. S18. Butein induces Ucp1 expression in human adipocytes through PI3K signaling pathway.
(a) Inhibitory effects of butein on Akt activity. Human adipocytes were treated with butein and PI3K mediated Akt phosphorylation was determined by western blotting. Representative data from two independent experiments were shown. (b) Induction of Ucp1 mRNA expression in human adipocytes by butein (20 μM). (c) Effects of butein on Prdm4 mRNA expression. Data represent means ± s.e.m. of three independent experiments. Data indicate means ± s.e.m. Statistical significance was determined by student’s t-test (* P < 0.05).

## Slide 36
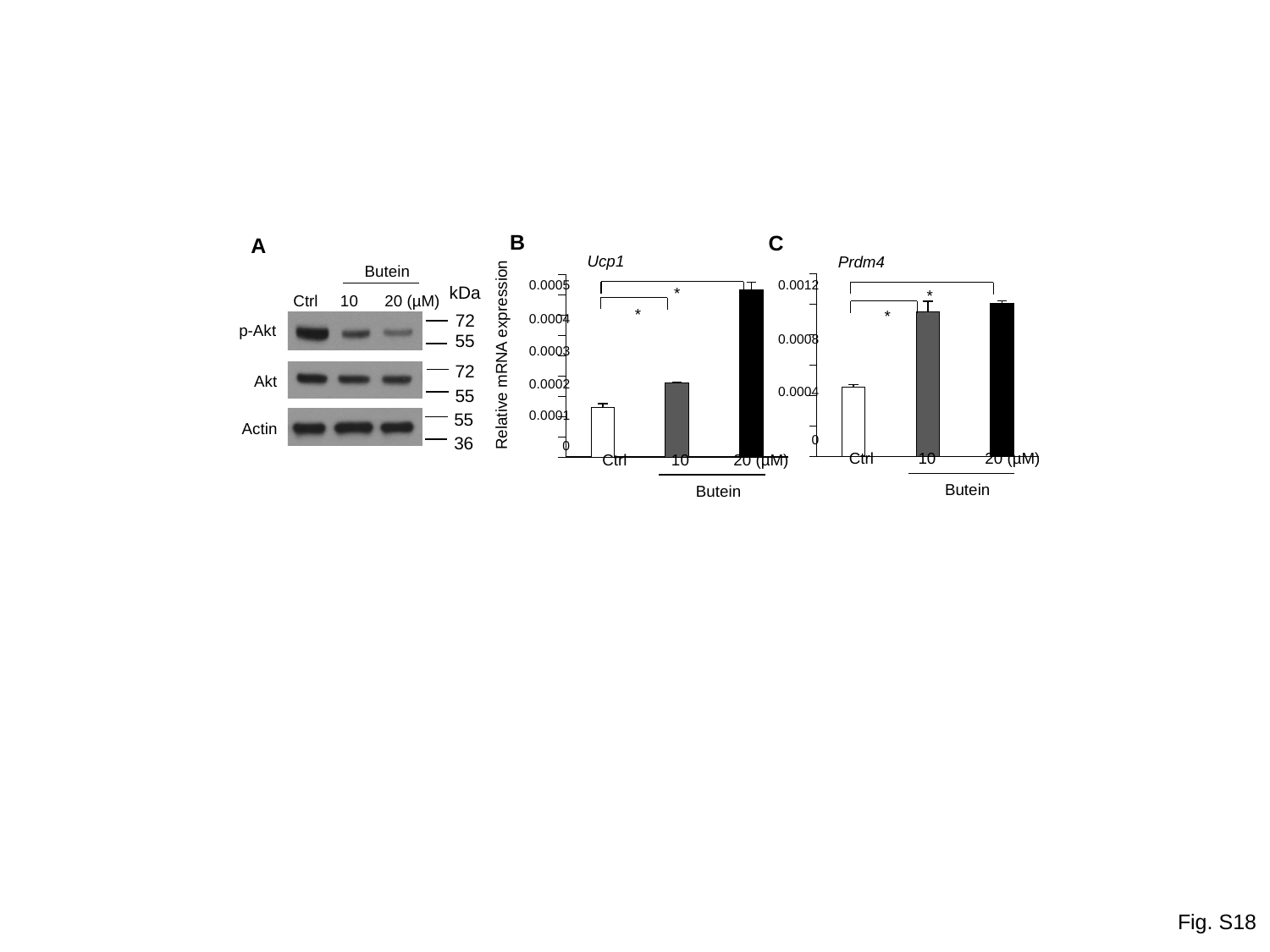

B
C
A
Ucp1
Prdm4
0.0005
0.0004
0.0003
0.0002
0.0001
 0
0.0012
0.0008
0.0004
 0
Butein
Relative mRNA expression
### Chart
| Category | |
|---|---|
| ctrl | 0.0004546511095196614 |
| but 10 | 0.0009472618841722893 |
| but 20 | 0.0010068681531282544 |
### Chart
| Category | |
|---|---|
| ctrl | 1.2174540998449018e-07 |
| but 10 | 1.8218356185318742e-07 |
| but 20 | 4.1100218897303287e-07 |kDa
*
*
 Ctrl 10 20 (µM)
*
*
72
p-Akt
55
72
 Akt
55
55
 Actin
36
 Ctrl 10 20 (µM)
 Ctrl 10 20 (µM)
Butein
Butein
Fig. S18

## Slide 37
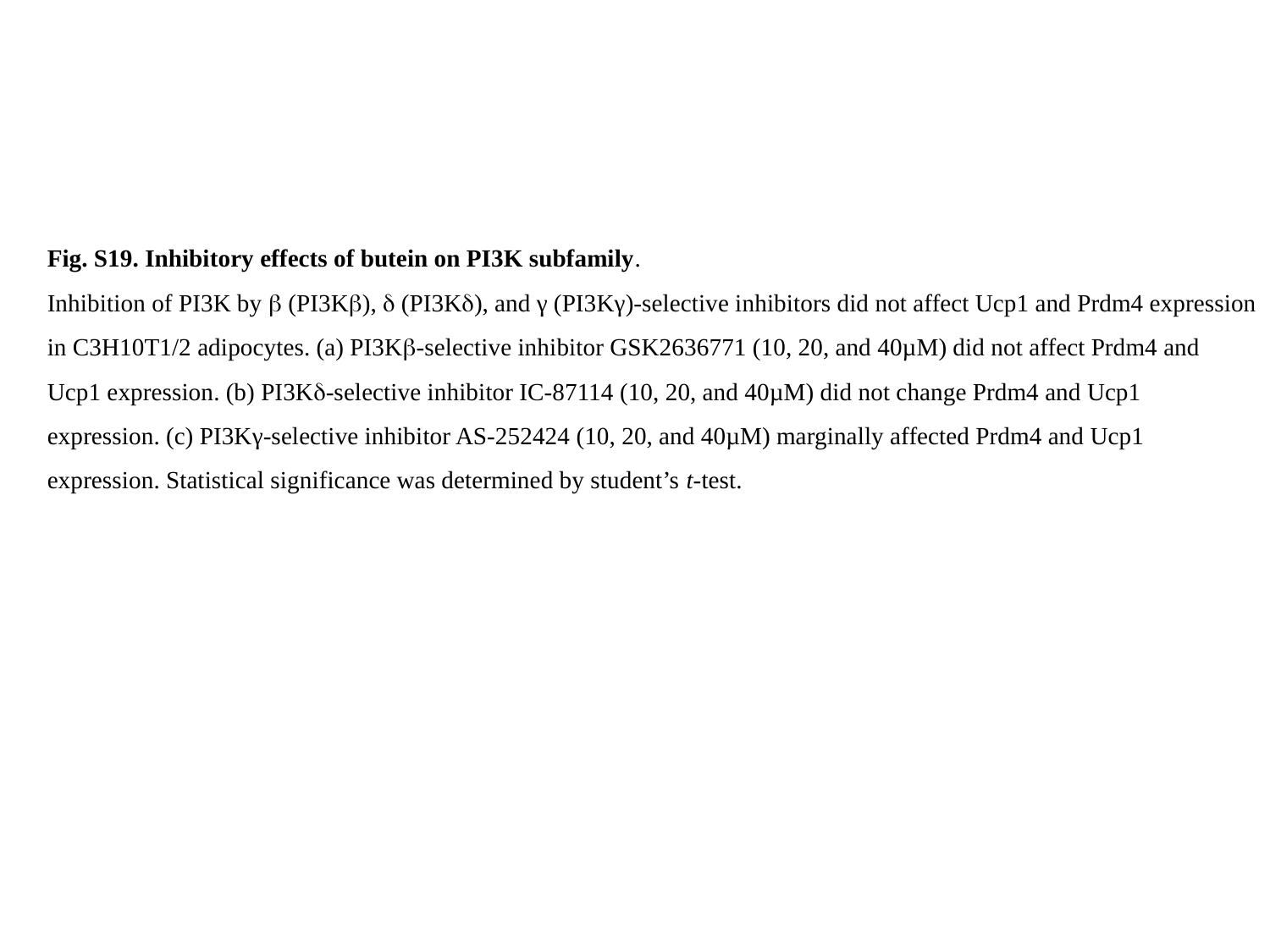

Fig. S19. Inhibitory effects of butein on PI3K subfamily.
Inhibition of PI3K by β (PI3Kβ),  (PI3Kδ), and γ (PI3Kγ)-selective inhibitors did not affect Ucp1 and Prdm4 expression in C3H10T1/2 adipocytes. (a) PI3Kβ-selective inhibitor GSK2636771 (10, 20, and 40µM) did not affect Prdm4 and Ucp1 expression. (b) PI3Kδ-selective inhibitor IC-87114 (10, 20, and 40µM) did not change Prdm4 and Ucp1 expression. (c) PI3Kγ-selective inhibitor AS-252424 (10, 20, and 40µM) marginally affected Prdm4 and Ucp1 expression. Statistical significance was determined by student’s t-test.

## Slide 38
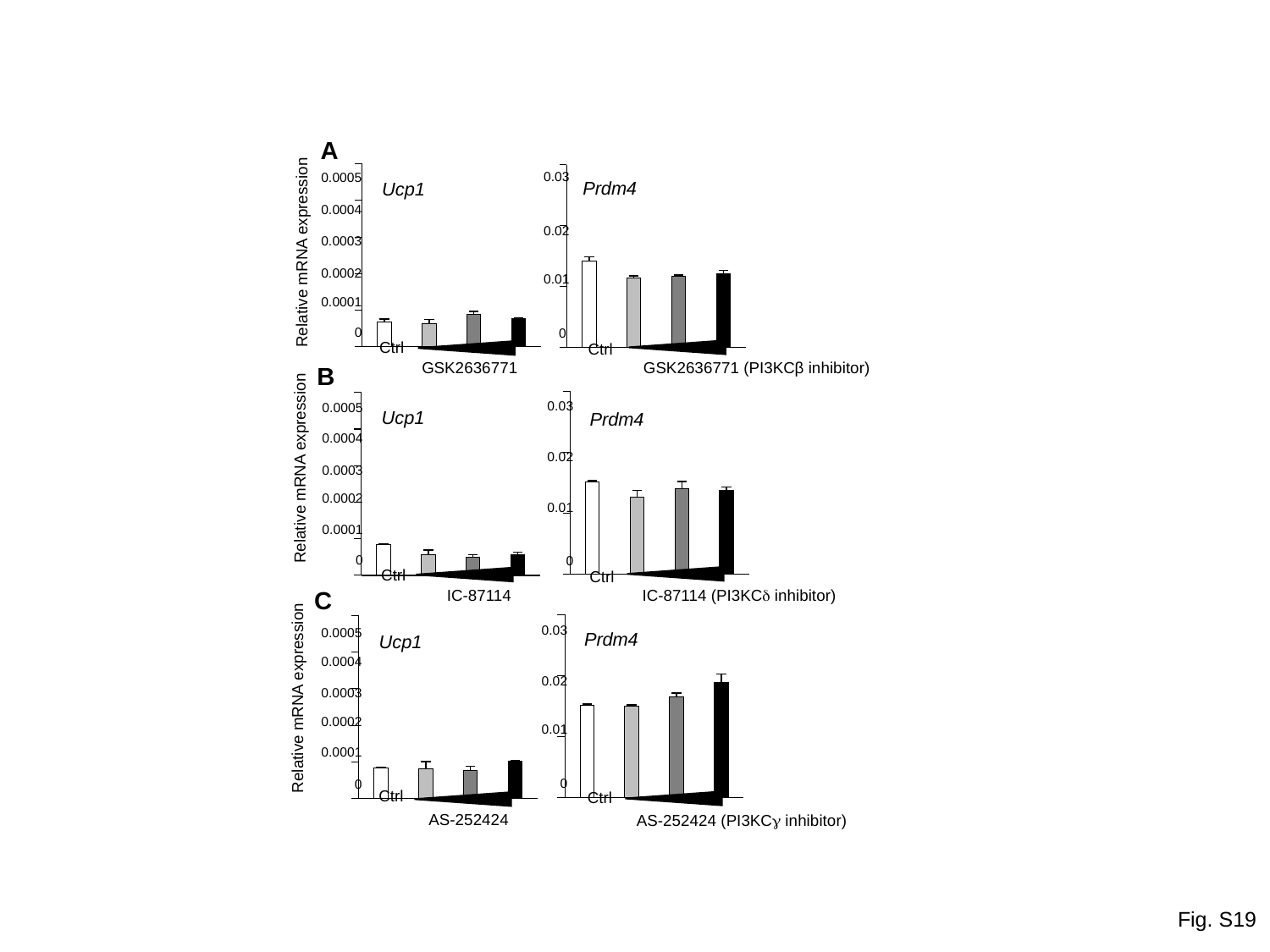

A
Relative mRNA expression
### Chart
| Category | |
|---|---|
| con 6h | 6.776444693556711e-05 |
| gsk 10 6h | 6.222960284694495e-05 |
| gsk 20 6h | 8.9189962600097e-05 |
| gsk 40 6h | 7.62561197805503e-05 |
### Chart
| Category | |
|---|---|
| con 6h | 0.014190205250772796 |
| gsk 10 6h | 0.011441466946601607 |
| gsk 20 6h | 0.011704361350247633 |
| gsk 40 6h | 0.012100518254005486 |0.03
0.02
0.01
 0
0.0005
0.0004
0.0003
0.0002
0.0001
 0
Prdm4
Ucp1
Ctrl
Ctrl
GSK2636771
GSK2636771 (PI3KCβ inhibitor)
B
Relative mRNA expression
### Chart
| Category | |
|---|---|
| con 6h | 0.015094205382358346 |
| ic87 10 6h | 0.012567858006463851 |
| ic87 20 6h | 0.014021044235893544 |
| ic87 40 6h | 0.013754573806566434 |
### Chart
| Category | |
|---|---|
| con 6h | 8.313343118011137e-05 |
| ic87 10 6h | 5.6241428176862604e-05 |
| ic87 20 6h | 4.879217422850638e-05 |
| ic87 40 6h | 5.6860685332212266e-05 |0.03
0.02
0.01
 0
0.0005
0.0004
0.0003
0.0002
0.0001
 0
Ucp1
Prdm4
Ctrl
Ctrl
C
IC-87114 (PI3KCδ inhibitor)
IC-87114
Relative mRNA expression
### Chart
| Category | |
|---|---|
| con 6h | 0.015094205382358346 |
| AS25 10 6h | 0.014929920950472323 |
| AS25 20 6h | 0.016527016893020045 |
| AS25 40 6h | 0.018910805697735705 |
### Chart
| Category | |
|---|---|
| con 6h | 8.313343118011137e-05 |
| AS25 10 6h | 7.984410928540863e-05 |
| AS25 20 6h | 7.695838379161968e-05 |
| AS25 40 6h | 0.0001010022925960258 |0.03
0.02
0.01
 0
0.0005
0.0004
0.0003
0.0002
0.0001
 0
Prdm4
Ucp1
Ctrl
Ctrl
AS-252424
AS-252424 (PI3KCγ inhibitor)
Fig. S19

## Slide 39
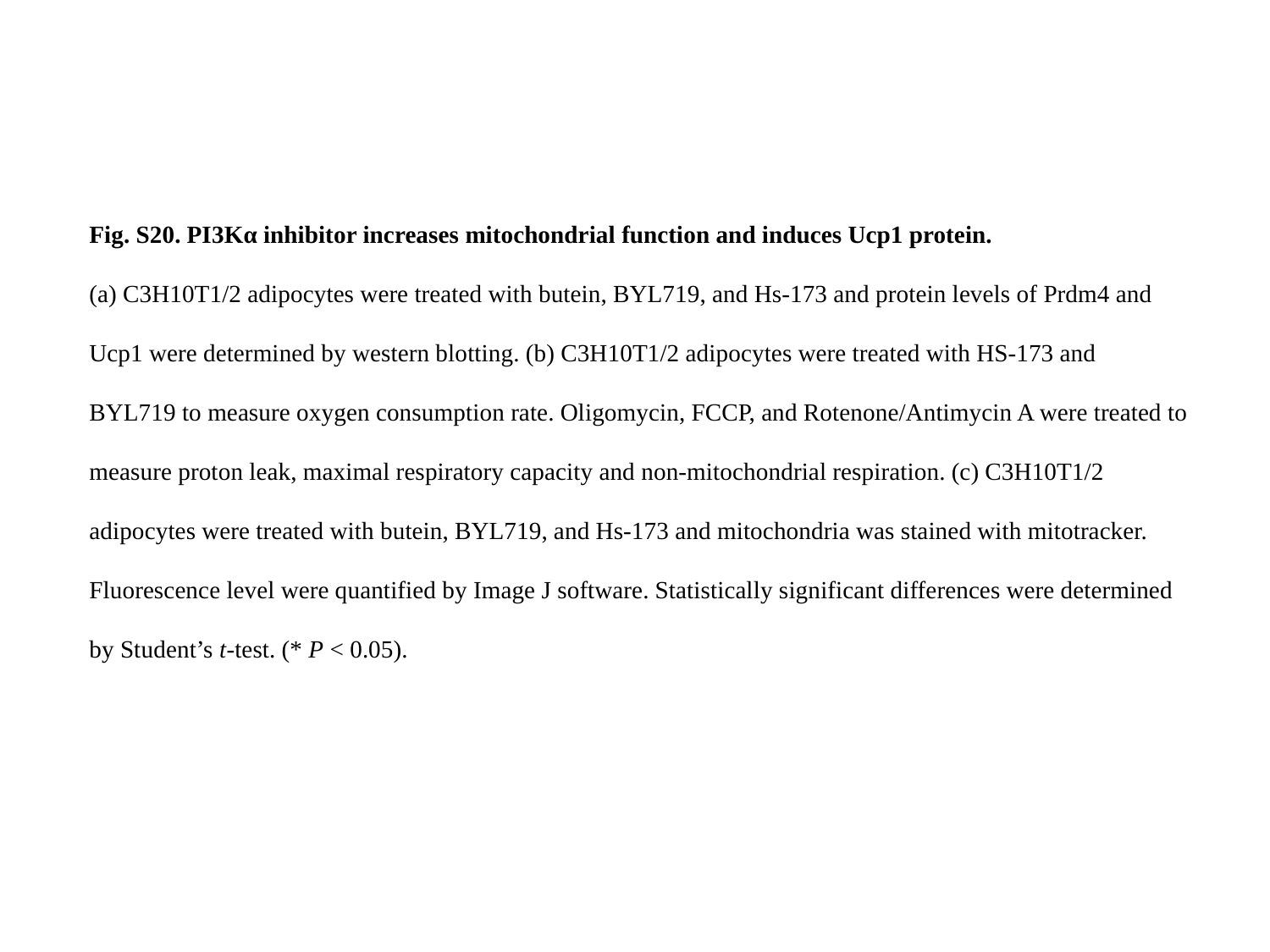

Fig. S20. PI3Kα inhibitor increases mitochondrial function and induces Ucp1 protein.
(a) C3H10T1/2 adipocytes were treated with butein, BYL719, and Hs-173 and protein levels of Prdm4 and Ucp1 were determined by western blotting. (b) C3H10T1/2 adipocytes were treated with HS-173 and BYL719 to measure oxygen consumption rate. Oligomycin, FCCP, and Rotenone/Antimycin A were treated to measure proton leak, maximal respiratory capacity and non-mitochondrial respiration. (c) C3H10T1/2 adipocytes were treated with butein, BYL719, and Hs-173 and mitochondria was stained with mitotracker. Fluorescence level were quantified by Image J software. Statistically significant differences were determined by Student’s t-test. (* P < 0.05).

## Slide 40
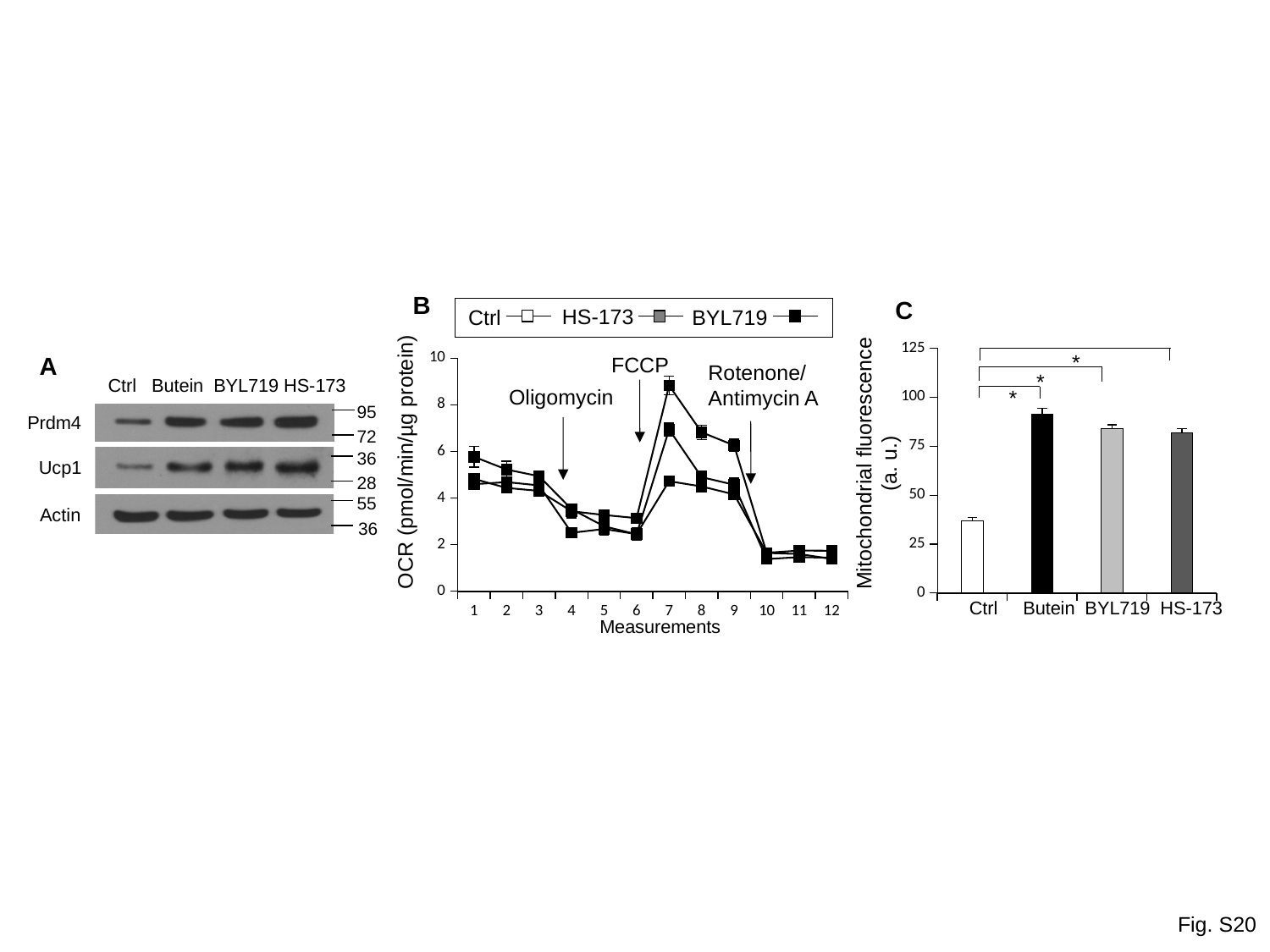

B
C
HS-173
BYL719
Ctrl
Mitochondrial fluorescence
(a. u.)
OCR (pmol/min/µg protein)
### Chart
| Category | |
|---|---|
| con | 36.889 |
| con + But | 91.33453333333334 |
| con + BYL | 83.97393333333333 |
| con + HS | 81.62353333333336 |*
A
### Chart
| Category | | | |
|---|---|---|---|FCCP
Rotenone/
Antimycin A
*
Ctrl Butein BYL719 HS-173
Oligomycin
*
95
Prdm4
72
36
Ucp1
28
55
Actin
36
Ctrl Butein BYL719 HS-173
Measurements
Fig. S20

## Slide 41
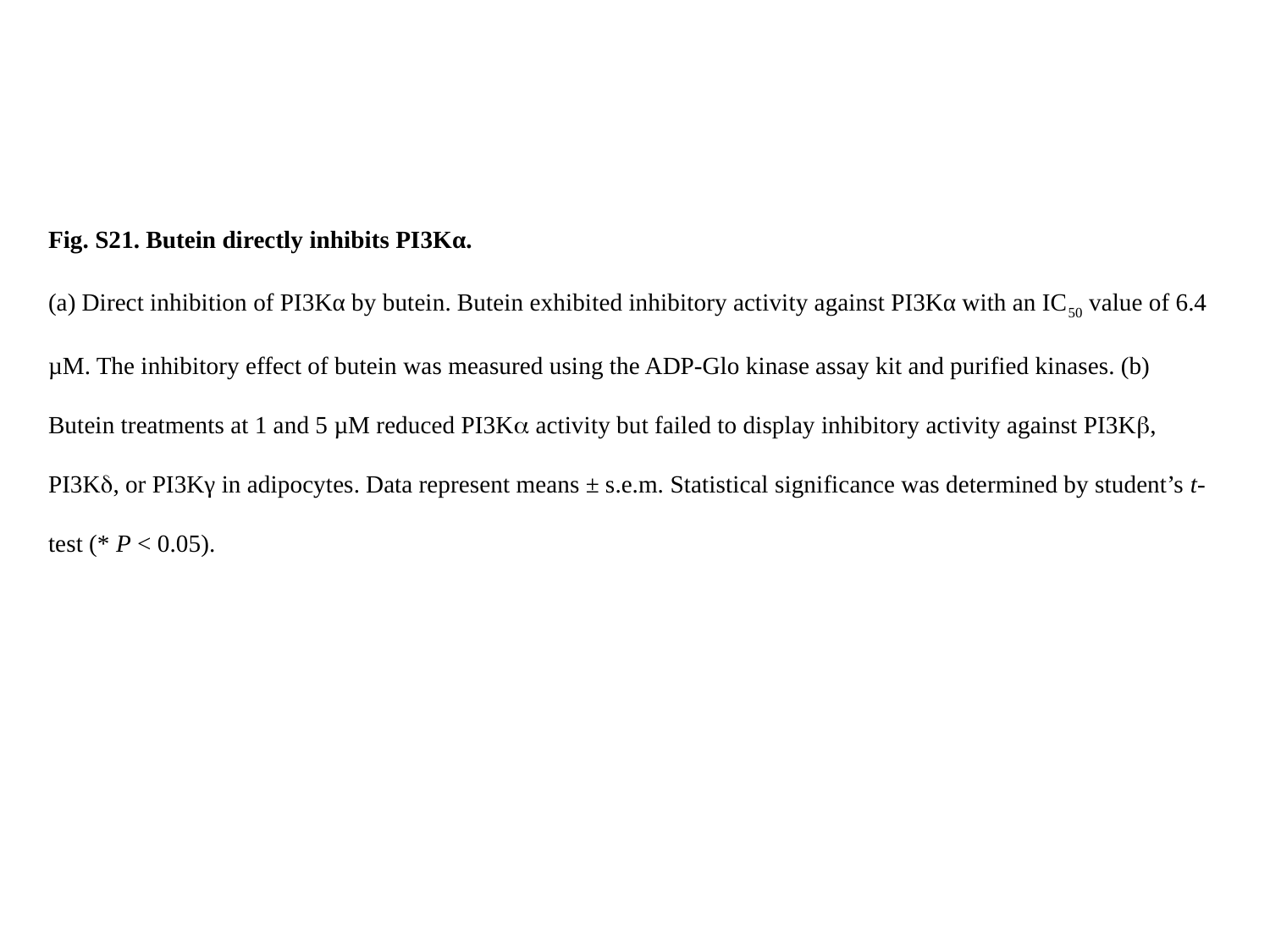

Fig. S21. Butein directly inhibits PI3Kα.
(a) Direct inhibition of PI3Kα by butein. Butein exhibited inhibitory activity against PI3Kα with an IC50 value of 6.4 µM. The inhibitory effect of butein was measured using the ADP-Glo kinase assay kit and purified kinases. (b) Butein treatments at 1 and 5 µM reduced PI3Kα activity but failed to display inhibitory activity against PI3Kβ, PI3Kδ, or PI3Kγ in adipocytes. Data represent means ± s.e.m. Statistical significance was determined by student’s t-test (* P < 0.05).

## Slide 42
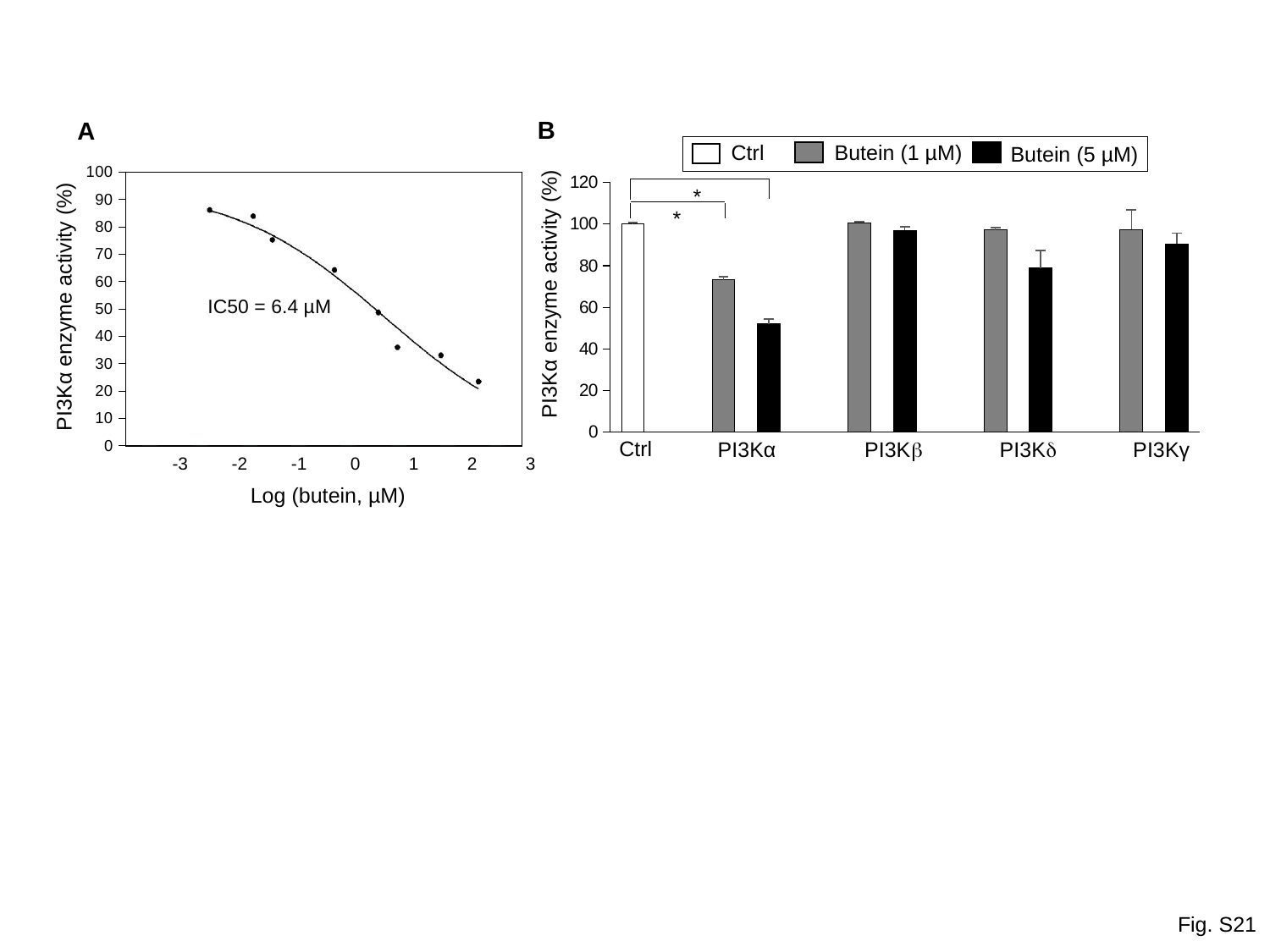

B
A
Butein (1 µM)
Ctrl
Butein (5 µM)
### Chart
| Category | |
|---|---|
| -2 | 0.0 |
| -1.3009999999999999 | 0.0 |
| -1 | 0.0 |
| 0 | 0.0 |
| 0.69899999999999995 | 0.0 |
| 1 | 0.0 |
| 1.6990000000000001 | 0.0 |
| 2.3010000000000002 | 0.0 |PI3Kα enzyme activity (%)
### Chart
| Category | |
|---|---|
| con | 100.0 |
| | None |
| b 1 | 73.27992238721893 |
| b 5 | 52.0005633185723 |
| | None |
| b 1 | 100.63202247191012 |
| b 5 | 96.96629213483146 |
| | None |
| b 1 | 97.15066599648154 |
| b 5 | 78.78706961548127 |
| | None |
| b 1 | 97.30889519237763 |
| b 5 | 90.47930758600626 |PI3Kα enzyme activity (%)
*
*
IC50 = 6.4 µM
Ctrl
PI3Kα PI3Kβ PI3Kδ PI3Kγ
-3 -2 -1 0 1 2 3
Log (butein, µM)
Fig. S21

## Slide 43
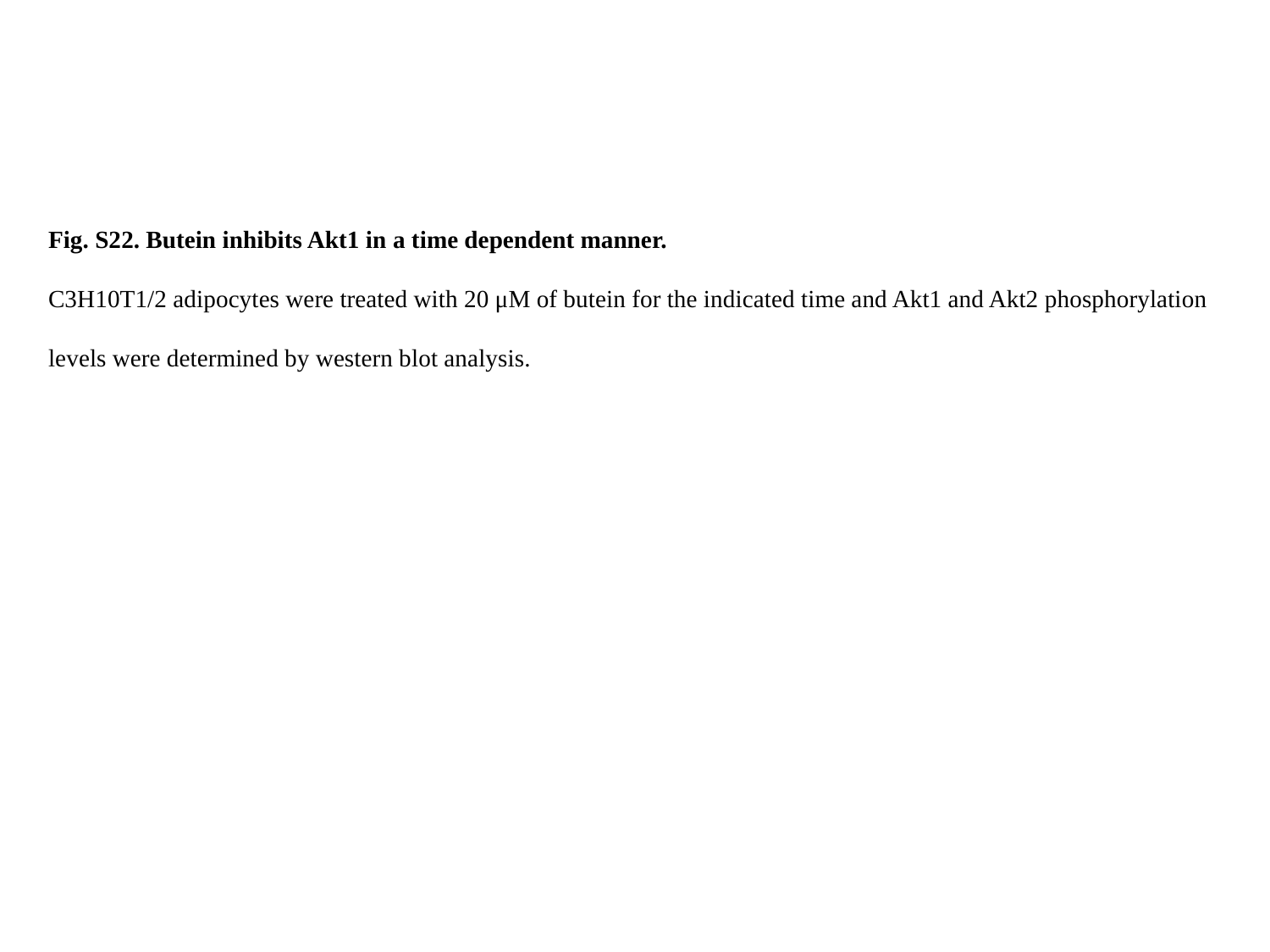

Fig. S22. Butein inhibits Akt1 in a time dependent manner.
C3H10T1/2 adipocytes were treated with 20 μM of butein for the indicated time and Akt1 and Akt2 phosphorylation levels were determined by western blot analysis.

## Slide 44
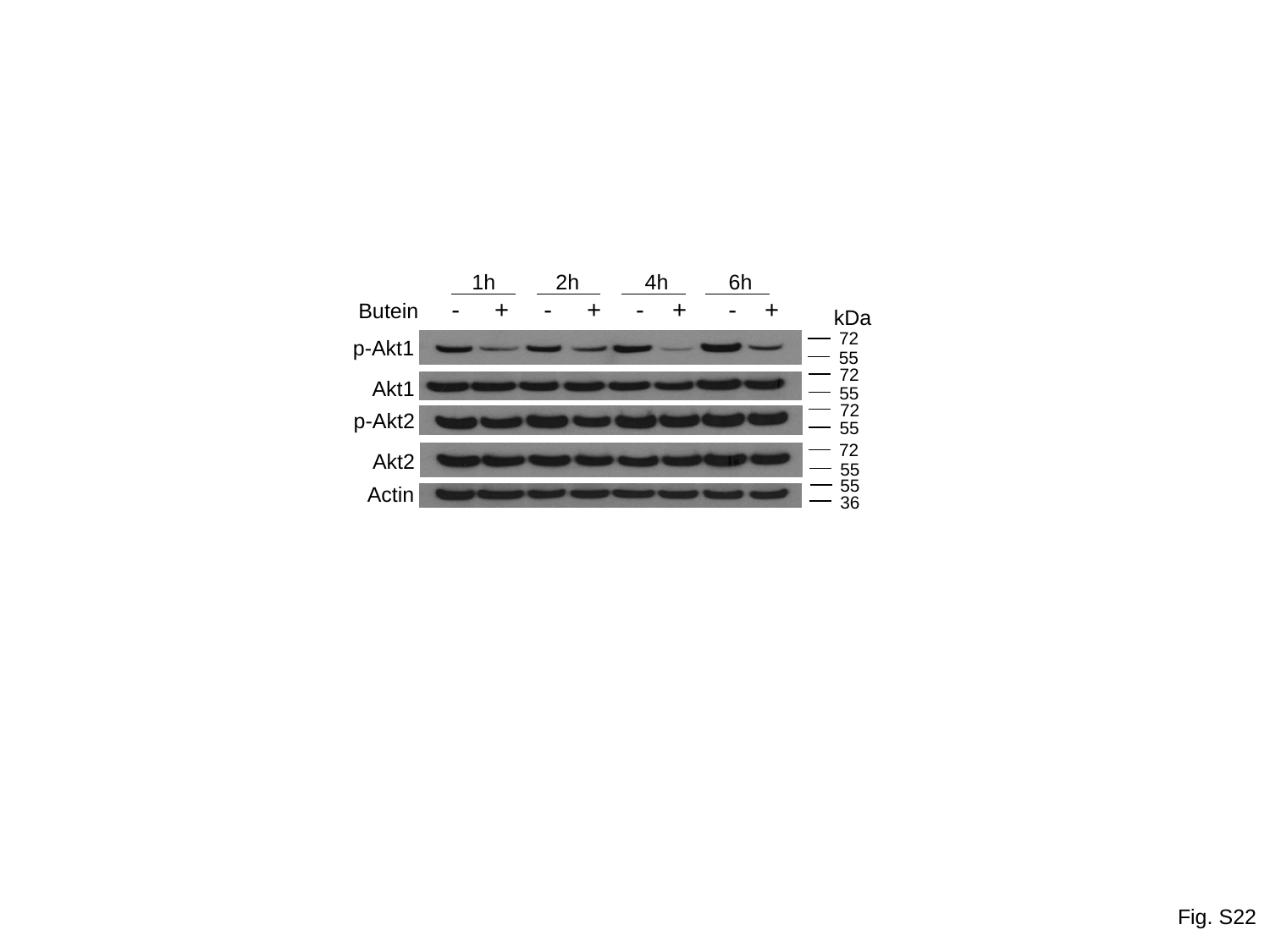

1h
2h
4h
6h
Butein - + - + - + - +
kDa
72
p-Akt1
55
72
Akt1
55
72
p-Akt2
55
72
Akt2
55
55
Actin
36
Fig. S22

## Slide 45
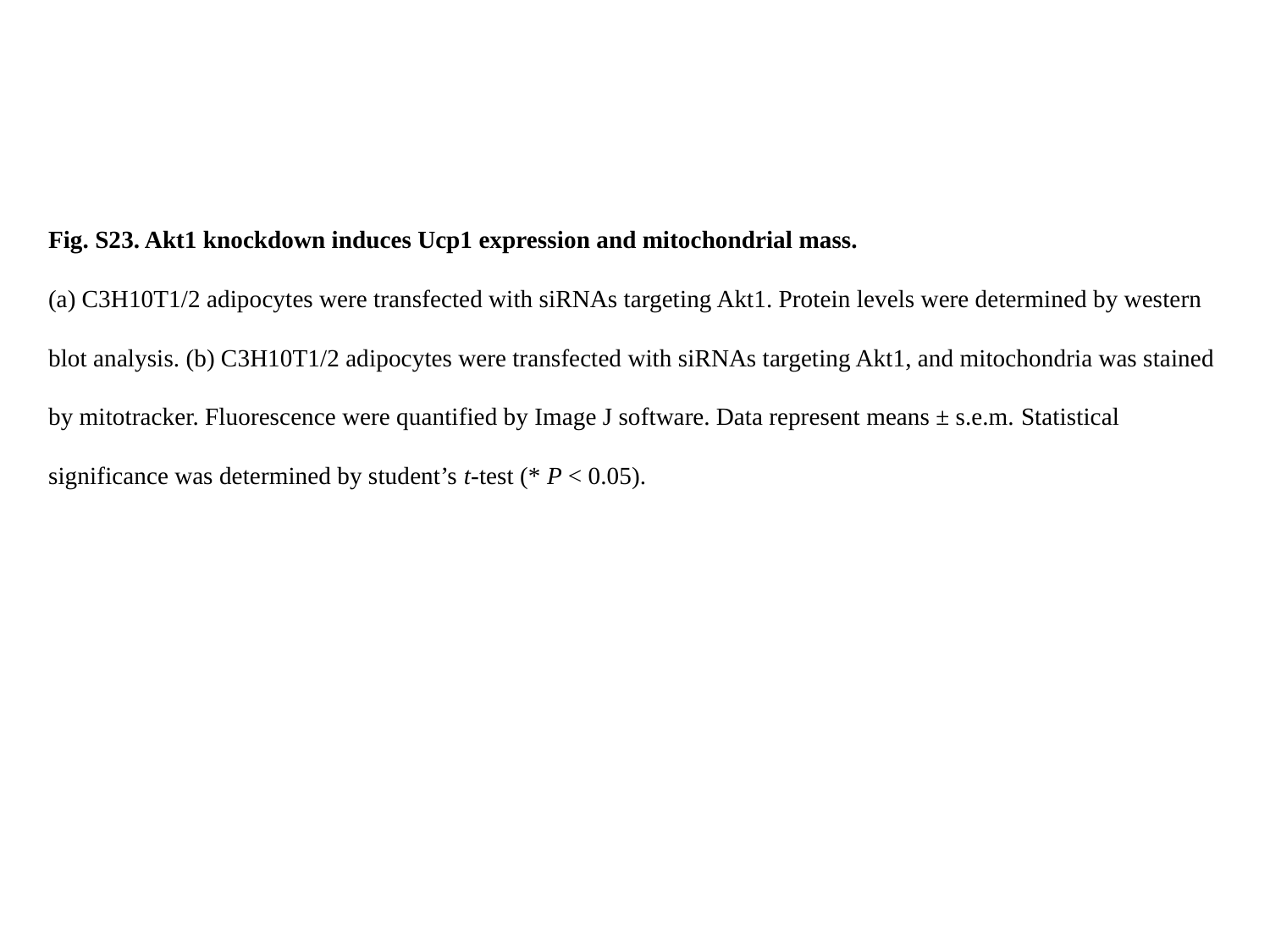

Fig. S23. Akt1 knockdown induces Ucp1 expression and mitochondrial mass.
(a) C3H10T1/2 adipocytes were transfected with siRNAs targeting Akt1. Protein levels were determined by western blot analysis. (b) C3H10T1/2 adipocytes were transfected with siRNAs targeting Akt1, and mitochondria was stained by mitotracker. Fluorescence were quantified by Image J software. Data represent means ± s.e.m. Statistical significance was determined by student’s t-test (* P < 0.05).

## Slide 46
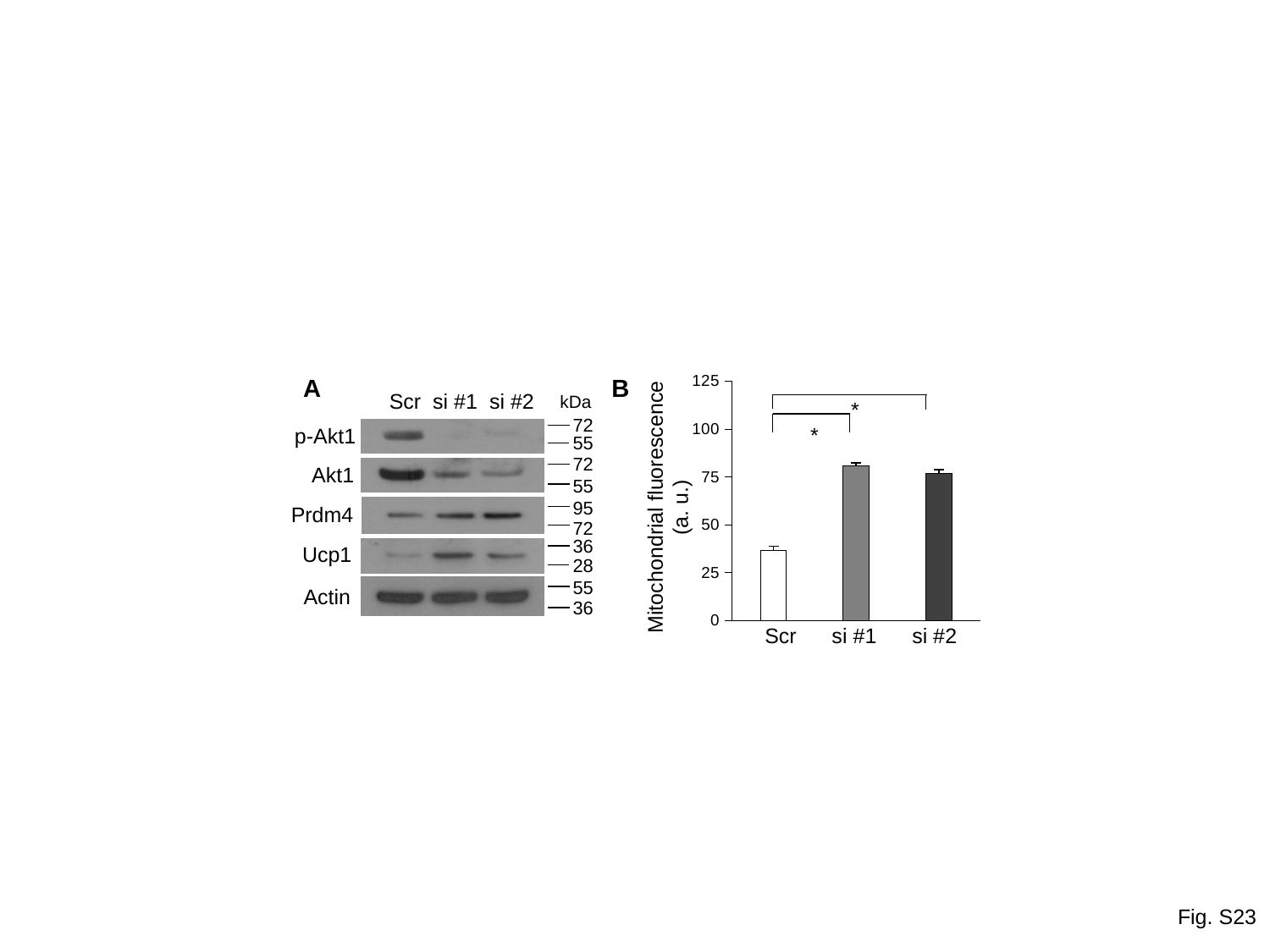

B
A
### Chart
| Category | |
|---|---|
| scr | 36.701 |
| si1 | 80.90333333333332 |
| si2 | 76.65499999999999 |Mitochondrial fluorescence
(a. u.)
Scr si #1 si #2
kDa
*
72
*
p-Akt1
55
72
Akt1
55
95
Prdm4
72
36
Ucp1
28
55
Actin
36
Scr si #1 si #2
Fig. S23
