# Supplementary material for: PI3Ka-Akt1-mediated Prdm4 induction in adipose tissue increases energy expenditure, inhibits weight gain, and improves insulin resistance in diet-induced obese mice
Source: Cell Death Dis. 2018 Aug 29;9(9):876. doi: 10.1038/s41419-018-0904-3 (PMC6115456; doi:10.1038/s41419-018-0904-3)
Supplement: Supplementary file 2 — Supplementary Figure Legends [file 41419_2018_904_MOESM2_ESM.docx]

**Supplementary Figures**

**Fig. S1. Butein treatment selectively increases thermogenic markers but decreases white adipocyte markers in adipose tissue of lean mice**.

Wild type lean mice were intraperitoneally injected with a control vehicle or butein (15mg/kg per day) for 2 weeks. Expression of general adipocyte markers (*Pparγ* and aP2), white adipocyte selective markers (*Retn* and *Nnmt*), and thermogenic adipocyte markers (*Ucp1*, *Prdm16*, *Cox8b*, and *Cidea*) in (a) inguinal adipose tissue (iWAT), (b) epididymal adipose tissue (eWAT), and (c) brown adipocyte tissues (BAT) was measured by real time PCR. Data represent means ± s.e.m. Statistically significant differences in gene expression between the control (n=6) and butein-treated mice (n=6) were determined by Student’s *t*-test (* *P* < 0.05; ** *P* < 0.005).

**Fig. S2.** **Effects of butein in HFD-fed obese mice on serum levels of cholesterol, LDL, TG, FFA, ALT, and AST.**

Plasma concentrations of total cholesterol, low density lipoprotein (LDL), triacylglycerol (TG), free fatty acids (FFA), alanine aminotransferase (ALT), and aspartate transaminase (AST) in C57BL/6 mice fed with HFD and administered a daily dose of butein at 5 mg/kg or 15 mg/kg. HFD (N = 5), HFD+ butein (HFD + butein 5 mg/kg, n=5), HFD + butein (15 mg/kg, n=5). Data represent means ± s.e.m. Statistical significance was determined by student *t*-test. (* *P* < 0.05).

**Fig. S3. Butein improves glucose and insulin tolerance in obese mice.**

(a) Glucose tolerance test and (b) insulin tolerance test in control and butein-treated groups (n=7 per group). Mice were fasted for 6 hours before the intraperitoneal injection of glucose or insulin for GTT and ITT. Tail blood samples were collected at different time points for measurement of blood glucose. Data r­­epresent means ± s.e.m. Statistically significant differences in the control and butein-treated mice were determined by two-way ANOVA (* *P* < 0.05).

**Fig. S4. Butein treatment increases thermogenic adipocyte genes and concomitantly decreases white adipocyte selective genes in adipose tissue.**

Male C57BL/6J mice were fed with high fat diet (HFD, 60% fat) and intraperitoneally injected with a control vehicle or butein (5mg/kg and 15mg/kg per day, n=7 per group) for 8 weeks. (a) Expression of general (*Pparγ* and *aP2*), white selective (*Retn* and *Nnmt*), and thermogenic selective genes (*Ucp1*, *Prdm16*, *Cox8b*, *Pgc-1a*, *Cidea*, *Cox7a*, and *Dio2*) in inguinal adipose tissue (iWAT) was measured by real time PCR. (b) Expression of general, white selective, and thermogenic selective genes in epididymal adipose tissue (eWAT). (c) Expression of general, white selective, and thermogenic selective genes in brown adipose tissue (BAT). Data represent means ± s.e.m. Statistical significance was determined by student *t*-test (* *P* < 0.05).

**Fig. S5. Daily food intake in butein or control treated HFD fed obese mice.**

(a) Corrected daily food intake was measured twice per week for 8 weeks. (b) Averages of daily food intake of control or butein treated groups (n=7 per group). (c) Corrected daily food intake was measured twice per week for 8weeks. (d) Average of corrected daily food intke of control or butein treated groups (n=7 per group). Data represent means ± s.e.m. No differences between groups are considered significant with P>0.05 using student *t*-test.

**Fig. S6. The effects of butein in mouse fed with low fat diet.**

(a) Control vehicle or butein treated mouse were fed with low fat diet (LFD) for 7 weeks and weighed twice per week. (b) Corrected food intake was measured twice per week for 7 weeks. Statistically significant differences between control vehicle treated and butein treated mouse were determined by Student’s *t*-test (* *P* < 0.05)

**Fig. S7. Butein prevents weight gains and improves glucose and insulin tolerance in HFD fed obese mice under thermoneutral conditions.**

Male C57BL/6J mice were fed with high fat diet (HFD, 60% fat) and intraperitoneally injected with a control vehicle or butein (15mg/kg per day, n=7 per group) for 8 weeks. All mice were housed in 30 °C to address effects of butein in thermoneutral condition. (a) Control vehicle and butein treated high fat diet fed mouse were housed for 8 weeks and body weight were measured twice a week. (b) Organ weights of eWAT, liver, kidney, and spleen were measured. (c), (d) Glucose tolerance test and insulin tolerance test of control vehicle or butein treated mouse fed with high fat diet. Mouse were fasted for 16 hours and 2g/kg glucose or 0.4U insulin were injected. Tail blood samples were collected at different time points to measure blood glucose levels. (e) Corrected food intake for whole study were measured every week for 7 weeks. Data represent mean ± s.e.m. and statistically significant differences were determined by student’s *t*-test for body weight and organ weight. Two-way ANOVA were used to determine statistical significance of GTT and ITT. (* *P* < 0.05; ** *P* < 0.005; *** *P* < 0.0005)

**Fig. S8. Butein selectively induces *Prdm4* expression in adipose tissue.**

Butein increases the expression of *Prdm4* in adipose tissues. Wild type lean mice were intraperitoneally injected with a control vehicle or butein (15mg/kg per day) for 3 weeks and expression of *Prdm4* was measured in various tissues. Data represent means ± s.e.m. (n=4). Statistical significance was determined by student *t*-test.

**Fig. S9. Transgenic expression of Prdm4 in non-adipose tissues.**

The fat-specific aP2 promoter was used to express Prdm4 in fat depots. Expression of *Prdm4* mRNA was measured in non-adipose tissues from non-transgenic (NonTg) and aP2-Prdm4 transgenic male mice (aP2-Prdm4 Tg).

**Fig. S10. Second Prdm4 transgenic line (aP2-Prdm4 Tg#2) are lean in HFD feeding.**

Body weight gain of nontransgenic (NonTg) and Prdm4 transgenic #2 (aP2-Prdm4 Tg#2) mice. (a) Male nontransgenic and transgenic mice were fed with a low fat diet (LFD, 10% fat) or high fat diet (HFD, 60% fat) for 8 weeks and were weighed once per week. (b) female nontransgenic (NonTg) and transgenic (aP2-Prdm4 Tg#2) mice were fed with LFD or HFD for 8 weeks and their body weights were measured. Data represent means ± s.e.m.

(c-d) Food intake was measured twice per week for 7 weeks in male. Statistically significant differences between NonTg and Prdm4Tg mice were determined by Student’s *t*-test (* *P* < 0.05)

**Fig. S11. Hematoxylin and eosin staining of paraffin-embedded liver and adipose fat pads after low fat diets (LFD) and high fat diet (HFD) feeding for 8 weeks.**

Representative Hematoxylin and Eosin (H&E) sections of liver, epididymal (eWAT), inguinal (iWAT), and brown adipose tissue (BAT) from LFD-fed and HFD-fed transgenic (aP2-Prdm4 Tg#1) and nontransgenic (NonTg) male mice. Scale bar, 100 µm.

**Fig. S12. Prdm4 transgenic female (aP2-Prdm4 Tg#1) mice are lean in HFD feeding.**

Body weight gain of nontransgenic (NonTg) and Prdm4 transgenic #1 female (aP2-Prdm4Tg#1) mice. (a) female nontransgenic (NonTg) and transgenic (aP2-Prdm4 Tg#2) mice were fed with LFD or HFD for 8 weeks and their body weights were measured. Data represent means ± s.e.m. (b) Differences in epididymal fat (eWAT) and inguinal fat (iWAT) weight gain in NonTg and Prdm4 Tg #1 female (aP2- Prdm4 Tg #1) mice fed with LFD or HFD. (c) Body composition of mice after 8 weeks of HFD fed NonTg and Prdm4Tg (aP2- Prdm4 Tg #1) female mice. Data represent mean ± s.e.m. and statistically significant differences between control NonTg and Prdm4 Tg mice were determined by Student’s *t*-test (* *P* < 0.05; ** *P* < 0.005).

**Fig. S13.** **aP2-Prdm4 Tg female mice increase energy expenditure in HFD feeding.**

(a-b) Energy expenditure was evaluated by measurement of oxygen consumption and carbon dioxide production. O_2_ consumption (a) and CO_2_ production (b) rates of littermate control NonTg and Prdm4 Tg female mice (ap2-Prdm4 Tg#1) were measured by indirect calorimetry using CLAMS after 6 weeks on HFD (n=6 per group). Bar graph (right panel) represents the average of O_2_ consumption or CO_2_ production in each group. (c) Food intake was measured twice per week for 7 weeks (n=6 per group). (d-e) Total physical activities (d) and ambulatory activities (e) of NonTg and Prdm4 Tg HFD fed mice (n=6 per group). Data represent mean ± s.e.m. and statistically significant differences between control NonTg and Prdm4 Tg mice were determined by Student’s *t*-test (* *P* < 0.05).

**Fig. S14. aP2-Prdm4 female mice improve glucose homeostasis.** (a) Glucose tolerance test and (b) insulin tolerance test in control NonTg female (n = 6) and aP2-Prdm4 Tg female mice (n = 6). Mice were fed with HFD for 6 and 7 weeks before measurement of glucose levels for GTT and ITT experiments, respectively. Mice were fasted for 6 h before intraperitoneal injection of glucose (1 g/kg) or insulin (0.75U/kg). Tail blood samples were collected at different time points to measure blood glucose levels. Data represent mean ± s.e.m. and statistically significant differences were determined by two-way ANOVA (* *P* < 0.05). (c) Rectal temperature of NonTg and aP2-Prdm4 Tg mice during cold exposure (4 °C) (n=6 per group). Data represent mean ± s.e.m. and statistically significant differences between control NonTg and aP2-Prdm4 Tg mice were determined by two-way ANOVA (* *P* < 0.05; ** *P* < 0.005).

**Fig. S15. Hematoxylin and eosin staining of paraffin-embedded liver and adipose fat pads after low fat diets (LFD) and high fat diet (HFD) feeding for 8 weeks.**

Representative Hematoxylin and Eosin (H&E) sections of liver, epididymal (eWAT), inguinal (iWAT), and brown adipose tissue (BAT) from LFD-fed and HFD-fed transgenic (aP2-Prdm4 Tg#1) and nontransgenic (NonTg) female mice. Scale bar, 100 µm.

**Fig. S16. Selective inhibition on PI3Kα kinase by butein.**

The effects of butein on kinase inhibition was examined on a kinase panel of 51 different kinases. Percentage inhibition of protein kinases following treatment with butein at 20 μM was shown. Values are means of duplicate assays performed using recombinant kinases and substrates.

**Fig. S17.** **Inhibition of PKA, PKC, and MAPK activities by specific inhibitors.**

C3H10T1/2 adipocytes were treated with 20 μM of H89 (PKA inhibitor), Go6839 (PKC inhibitor), and U0126 (ERK kinase inhibitor) for 6 - 24 hours and phosphorylated forms of PKA, PKC, and ERK were determined by western blotting.

**Fig. S18.** **Butein induces Ucp1 expression in human adipocytes through PI3K signaling pathway.**

(a) Inhibitory effects of butein on Akt activity. Human adipocytes were treated with butein and PI3K mediated Akt phosphorylation was determined by western blotting. Representative data from two independent experiments were shown. (b) Induction of *Ucp1* mRNA expression in human adipocytes by butein (20 μM). (c) Effects of butein on *Prdm4* mRNA expression. Data represent means ± s.e.m. of three independent experiments. Data indicate means ± s.e.m. Statistical significance was determined by student’s *t*-test (* *P* < 0.05).

**Fig. S19.** **Inhibitory effects of butein on PI3K subfamily**.

Inhibition of PI3K by β (PI3Kβ), δ (PI3Kδ), and γ (PI3Kγ)-selective inhibitors did not affect Ucp1 and Prdm4 expression in C3H10T1/2 adipocytes. (a) PI3Kβ-selective inhibitor GSK2636771 (10, 20, and 40µM) did not affect Prdm4 and Ucp1 expression. (b) PI3Kδ-selective inhibitor IC-87114 (10, 20, and 40µM) did not change Prdm4 and Ucp1 expression. (c) PI3Kγ-selective inhibitor AS-252424 (10, 20, and 40µM) marginally affected Prdm4 and Ucp1 expression. Statistical significance was determined by student’s *t*-test.

**Fig. S20. PI3Kα inhibitor increases mitochondrial function and induces Ucp1 protein.**

(a) C3H10T1/2 adipocytes were treated with butein, BYL719, and Hs-173 and protein levels of Prdm4 and Ucp1 were determined by western blotting. (b) C3H10T1/2 adipocytes were treated with HS-173 and BYL719 to measure oxygen consumption rate. Oligomycin, FCCP, and Rotenone/Antimycin A were treated to measure proton leak, maximal respiratory capacity and non-mitochondrial respiration. (c) C3H10T1/2 adipocytes were treated with butein, BYL719, and Hs-173 and mitochondria was stained with mitotracker. Fluorescence level were quantified by Image J software. Statistically significant differences were determined by Student’s *t*-test. (* *P* < 0.05).

**Fig. S21. Butein directly inhibits PI3Kα.**

(a) Direct inhibition of PI3Kα by butein. Butein exhibited inhibitory activity against PI3Kα with an IC_50_ value of 6.4 µM. The inhibitory effect of butein was measured using the ADP-Glo kinase assay kit and purified kinases. (b) Butein treatments at 1 and 5 µM reduced PI3Kα activity but failed to display inhibitory activity against PI3Kβ, PI3Kγ, or PI3Kδ in adipocytes. Data represent means ± s.e.m. Statistical significance was determined by student’s *t*-test (* *P* < 0.05).

**Fig. S22. Butein inhibits Akt1 in a time dependent manner.**

C3H10T1/2 adipocytes were treated with 20 μM of butein for the indicated time and Akt1 and Akt2 phosphorylation levels were determined by western blot analysis.

**Fig. S23. Akt1 knockdown induces Ucp1 expression and mitochondrial mass.**

(a) C3H10T1/2 adipocytes were transfected with siRNAs targeting Akt1. Protein levels were determined by western blot analysis. (b) C3H10T1/2 adipocytes were transfected with siRNAs targeting Akt1, and mitochondria was stained by mitotracker. Fluorescence were quantified by Image J software. Data represent means ± s.e.m. Statistical significance was determined by student’s *t*-test (* *P* < 0.05).
